# Divergent Regulatory Effects of Jasmonic Acid on Tomato Lycopene Biosynthesis Under Light and Dark Conditions

**DOI:** 10.1002/advs.202513249

**Published:** 2026-03-02

**Authors:** Jiayi Xu, Lingfeng He, Jialong Zhang, Hao Cui, Hongxin Li, Haijun Zhang, Jiaojiao Zhang, Lun Liu, Kunpeng Xu, Yang‐Dong Guo, Na Zhang

**Affiliations:** ^1^ College of Horticulture China Agricultural University Beijing China; ^2^ College of Food Science and Engineering Ningbo University Ningbo China; ^3^ Supervision, Inspection and Test Center of Vegetable Seed Quality of Ministry of Agriculture and Rural Affairs State Key Laboratory of Vegetable Biobreeding Beijing Vegetable Research Center (BVRC) Beijing Academy of Agriculture and Forestry Science (BAAFS) Beijing China; ^4^ Hebei Key Laboratory of Horticultural Germplasm Excavation and Innovative Utilization, College of Horticulture Science and Technology Hebei Normal University of Science and Technology Qinhuangdao China; ^5^ College of Horticulture Anhui Agricultural University Hefei China; ^6^ College of Agriculture Fujian Agriculture and Forestry University Fuzhou China

**Keywords:** carotenoids, fruit coloration, jasmonate, light signal, phytochrome interacting factor

## Abstract

Jasmonates influence carotenoids biosynthesis, the pigments responsible for tomato fruit coloration, but their effect on carotenoids synthesis remains controversial. Lycopene is the predominant carotenoid in ripe tomato fruits, accounting for more than 90% of the total carotenoid content in the fruit. Our study clarified this paradox by demonstrating that methyl jasmonate (MeJA) affects post‐harvest tomato lycopene accumulation differently depending on light conditions and we identified SlPIF1a as the central regulatory factor mediating this light‐JA crosstalk. In light, MeJA enhances lycopene synthesis by directly activating the expression of the *SlPSY1* gene through the SlMYC2 transcription factor. SlMYC2 also inhibits the expression of *SlPIF1a*, encoding a negative regulator of light signal that degrades in light and accumulates in darkness. In dark conditions, the accumulated SlPIF1a interacts with SlMYC2 inhibiting its activation on *SlPSY1* expression. Additionally, the MeJA‐induced acetyltransferase SlNATA1 interacts with and acetylates SlPIF1a, enhancing its repression on *SlPSY1*. Our research uncovers a new mechanism for the dual regulation of lycopene synthesis by jasmonic acid under different light conditions.

## Graphical abstract

This study reveals the mechanism by which jasmonic acid (JA) regulates lycopene synthesis under light and dark conditions. In light, JA activates SlMYC2, which suppresses *SlPIF1a* and promotes *SlPSY1* expression. In darkness, JA induces the acetyltransferase SlNATA1, which acetylates the dark‐accumulated SlPIF1a, thereby repressing *SlPSY1* expression.

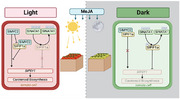

## 1 Introduction

Tomato fruits are widely consumed worldwide due to their appealing flavor and nutritional benefits. Carotenoids, the primary pigments and essential nutritional components in tomatoes, play crucial roles in maintaining human health and preventing various diseases [1, 2, 3]. Moreover, carotenoid content serves as a key indicator for evaluating the ripening process of tomato fruits [4].

In higher plants, the precursors of carotenoid biosynthesis are isopentyl diphosphate (IPP) and its isomer, dimethylallyl diphosphate (DMAPP), synthesized via the plastid‐localized 2‐C‐methyl‐D‐erythritol 4‐phosphate (MEP) pathway. The enzyme 1‐deoxy‐D‐xylulose 5‐phosphate synthase (SlDXS1) is the rate‐limiting step in this pathway [5]. IPP and DMAPP condense under the catalysis of geranylgeranyl pyrophosphate synthase (GGPS) to form geranylgeranyl pyrophosphate (GGPP) [6]. Two GGPP molecules are polymerized by phytoene synthase (PSY) to generate phytoene, the first carotenoid in the biosynthetic pathway [7]. In tomato fruits, phytoene undergoes dehydrogenation and isomerization catalyzed by phytoene desaturase (PDS), ζ‐carotene desaturase (ZDS), carotene isomerase (CRTISO), and ζ‐carotene isomerase (Z‐ISO), ultimately forming all‐trans‐lycopene. Linear lycopene molecules are then cyclized by lycopene β/ε‐cyclases (LCYB or LCYE) to yield α‐ and β‐carotene [8]. Subsequently, α‐ and β‐carotene undergo oxidative modifications to produce lutein, zeaxanthin, violaxanthin, and neoxanthin [9, 10, 11, 12].

PSY functions as a major rate‐limiting enzyme in carotenoid biosynthesis [12]. In tomato, three homologous *PSY* genes have been identified. Overexpression of *SlPSY1* markedly increases both the biosynthetic rate and total carotenoid content. [13] Recent studies revealed that *SlPSY1* primarily influences fruit coloration, *SlPSY2* regulates carotenoid accumulation in leaves, and *SlPSY3* controls carotenoid synthesis in roots [14].

Light is a vital environmental signal influencing carotenoid biosynthesis in tomato fruits. Alba et al. demonstrated that phytochromes (phys) regulate carotenoid biosynthesis in tomatoes through a mechanism independent of the ethylene signaling pathway, which primarily governs fruit ripening [15]. Shading and UV‐B irradiation also affect carotenoid accumulation [16, 17]. Red and blue light treatments significantly enhance carotenoid accumulation in harvested tomato fruits, with red light being more effective [18]. Similarly, red light increases carotenoid content in pepper [19]. Plants exhibit distinct developmental patterns under light and dark conditions, known respectively as photomorphogenesis and skotomorphogenesis. These differences depend on the reversible interconversion of phytochromes between two forms: the biologically inactive Pr and the active Pfr. Upon light exposure, Pr converts to Pfr, which translocates into the nucleus to regulate gene expression [20, 21]. In darkness, phytochrome‐interacting factors (PIFs) accumulate in the nucleus, promoting skotomorphogenesis characterized by rapid hypocotyl elongation and closed cotyledons. Under light, active phytochrome phyB interacts with PIFs, inducing their ubiquitination and degradation. This activates elongated hypocotyl 5 (HY5), triggering photomorphogenesis, which is marked by inhibited hypocotyl elongation, expanded photosynthetic leaves, and anthocyanin accumulation [22, 23]. Previous studies have shown that the transcription factor SlPIF1a suppresses lycopene biosynthesis in tomato fruits, whereas SlHY5 overexpression enhances lycopene accumulation [24, 25, 26].

Jasmonates (JAs) are naturally occurring plant hormones known to regulate various physiological processes, including carotenoid biosynthesis [27, 28, 29, 30, 31, 32]. In plants, the bioactive jasmonoyl‐L‐isoleucine (JA‐Ile) binds to its receptor CORONATINE INSENSITIVE 1 (COI1), leading to the degradation of JASMONATE ZIM‐DOMAIN (JAZ) repressor proteins and activation of downstream transcription factors such as MYC2 [33]. In tomato fruits, foliar spraying with methyl jasmonate (MeJA) increases carotenoid accumulation and upregulates the expression of carotenoid biosynthetic genes such as *SlPSY1* and *SlPDS* [27]. However, contrasting results were reported when MeJA was applied in lanolin to the fruit surface, significantly inhibiting lycopene synthesis [29]. In *Citrus sinensis*, CsMYC2 activates carotenoid biosynthetic genes including cleavage dioxygenase 4b (*CsCCD4b*), *CsPSY*, lycopene β‐cyclase (*CsLCYb*), and β‐carotene hydroxylase (*CsBCH*), whereas CsMPK6 negatively regulates carotenoid accumulation by phosphorylating CsMYC2 and reducing its stability [28].

Recent findings indicate that light and JA signals interact to modulate secondary metabolites such as anthocyanins. In *Artemisia annua*, JA promotes anthocyanin synthesis under light but has no effect in darkness [34]. The transcription factor AaMYB108 accumulates under light and mediates this JA‐induced pathway [35]. In *Solanum melongena*, SmMYB5 may integrate light and JA signaling to co‐regulate anthocyanin accumulation in fruits [36]. Similarly, in *Arabidopsis*, UV‐B light activates *LOX2*, increasing JA levels and enhancing anthocyanin synthesis for photoprotection [37]. In grape berries, MeJA vapor treatment stimulates anthocyanin accumulation under light but not under continuous darkness [38].

Although numerous studies have demonstrated that JA influences fruit ripening, cold tolerance, and disease resistance, its specific regulatory mechanisms in fruit maturation remain controversial. These inconsistencies have limited the practical use of JA in agricultural production. Drawing parallels with JA‐mediated regulation of anthocyanin synthesis, we hypothesized that the conflicting reports on JA's effects on carotenoid content may be associated with differences in light conditions. To elucidate the mechanism underlying JA's light‐dependent regulation of lycopene accumulation in postharvest tomato fruits, we first examined the phenotypic responses and confirmed that JA enhances lycopene biosynthesis under light but inhibits it in darkness. RT–qPCR analysis of key lycopene biosynthetic genes revealed that JA differentially regulates transcription—upregulating *SlPSY1* under light while repressing it in darkness, with *SlPSY1* showing the greatest response. Based on this expression pattern, we identified upstream regulators and characterized SlMYC2 from the JA pathway and SlPIF1a from the light signaling pathway as key transcription factors modulating *SlPSY1* expression. Integrating molecular evidence, we demonstrate that under light conditions, JA primarily activates SlMYC2 to enhance lycopene biosynthesis, whereas in darkness, repression is mediated mainly through SlPIF1a acetylation. Our study reveals a novel mechanism by which JA bidirectionally regulates lycopene biosynthesis in tomatoes depending on light conditions. This discovery provides an important theoretical framework for optimizing postharvest tomato handling through JA treatment. Specifically, JA‐treated fruits showed delayed ripening when stored in darkness during transportation but exhibited accelerated color development upon light exposure at retail. This approach offers a practical alternative to the combined use of ethylene inhibitors and ethylene induction cycles, thereby lowering production costs and simplifying logistics. Furthermore, as a defensive phytohormone, JA simultaneously enhances tomato resistance to gray mold and chilling injury during storage.

## 2 Results

### 2.1 MeJA Showed Opposite Effects on Carotenoid Accumulation in Tomato Fruits Under Light and Dark Conditions Independent of the Ethylene Pathway

**FIGURE 1:**
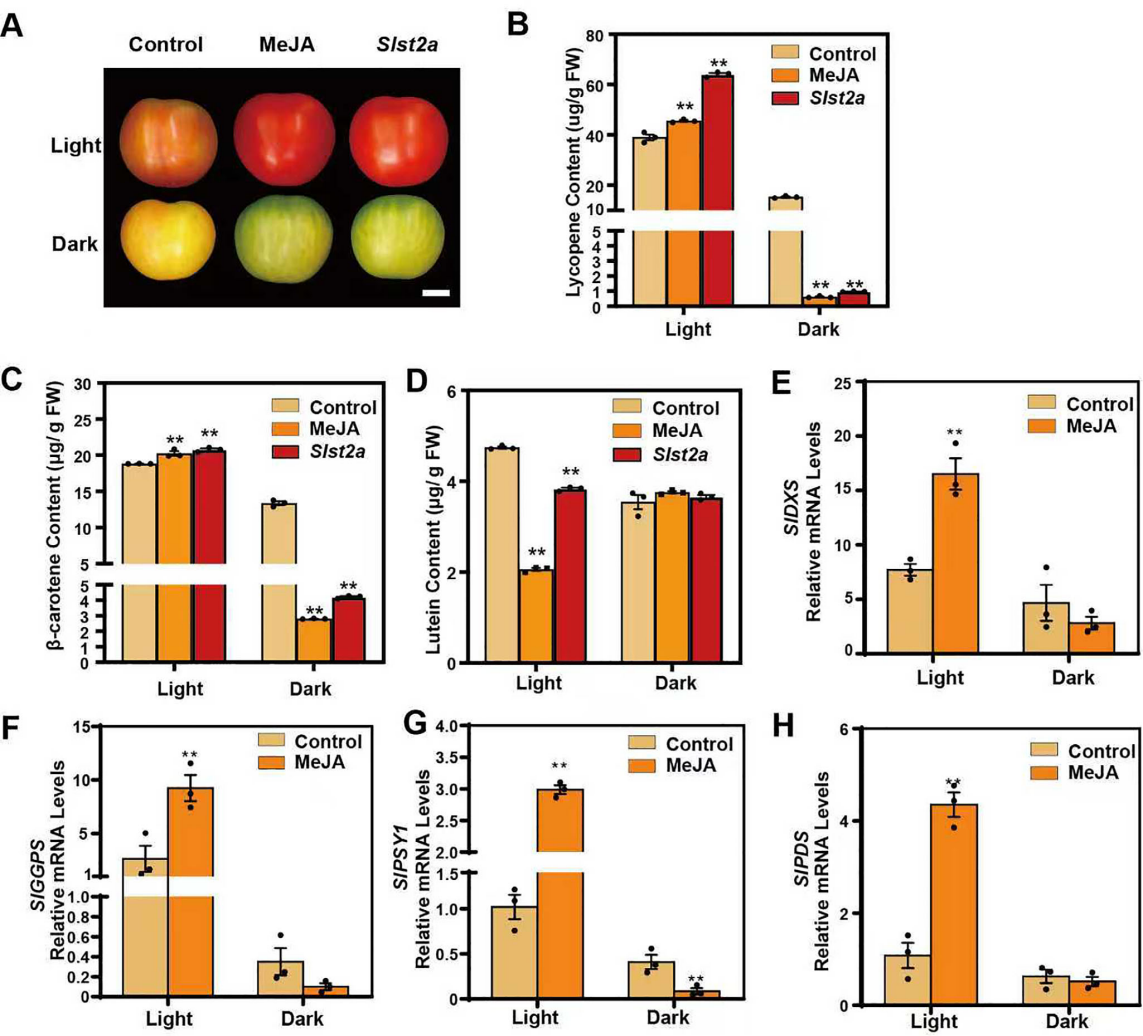
Jasmonate acid bidirectionally regulates carotenoid biosynthesis in tomato fruits. (A) Tomato cultivar Ailsa Craig (WT) fruits and *SlST2a* knockout line (*Slst2a*) were harvested at mature green stage (MG) and WT fruits were treated with 200 µM MeJA for 5 days. Fruits treated with aqueous solution of 0.2% ethanol were used as control (Control). Light groups were stored at 16 h light at 24°C and 8 h dark at 18°C. Dark groups were stored at 16 h at 24°C and 8 h at 18°C under dark conditions. Scale bar = 1 cm. Detailed analysis of lycopene (B), β‐carotene (C) and lutein (D) concentrations in fruit pericarps under light and dark conditions. The source data and statistical analysis is detailed in Supplement Data Set 1. The mRNA levels of *SlDXS* (E), *SlGGPS* (F), *SlPSY1* (G), *SlPDS* (H) were determined in Control and MeJA groups. Tomato housekeeping gene *SlUBQ* (Solyc01g056940) and *SlActin2* (Solyc11g005330) were used as internal control. Each sample contained pericarps from three different fruits as a biological replicate. Three replicates were performed per experiment. Values represent means ± SE. Asterisks indicate statistically significant differences analyzed by two‐way ANOVA (^*^ *p*≤0.05, ^**^ *p*≤0.01, Šídák's multiple comparisons test). Source data and statistical summary can be found in the Supplement Data Set 1.

Application of MeJA to postharvest tomato fruits at the MG stage revealed that JA influences carotenoid biosynthesis and fruit coloration differently depending on light exposure. Under light conditions, JA promoted carotenoid synthesis, whereas in darkness, the effect was reversed (Figure 1A). Detailed carotenoid profiling showed that MeJA‐treated fruits accumulated significantly higher levels of lycopene and β‐carotene but lower lutein under light; in contrast, lycopene and β‐carotene contents decreased in the dark with no change in lutein (Figure 1B–D). To further confirm this phenotype, we examined the carotenoid contents in *Slst2a* mutant fruits, in which JA was highly accumulated [39]. The results showed that, under both light and dark conditions, the changes in the levels of various carotenoids in *Slst2a* fruits were fully consistent with those observed upon exogenous MeJA treatment (Figure 1B–D). These findings indicate that JA enhances the biosynthesis of lycopene and β‐carotene under light but suppresses their formation in darkness, while its inhibitory effect on lutein occurs mainly under light conditions. RT‐qPCR analysis demonstrated that MeJA significantly upregulated the expression of key carotenoid biosynthetic genes under light, whereas under dark conditions, MeJA treatment led to an overall decline in the expression of these genes, with SlPSY1 showing the strongest repression (Figure 1E–H; Figure S1). Because plastids are the main organelles for carotenoid storage in tomato fruits, we also examined the expression of SlOR, the only currently identified key regulator of plastid development [40, 41]. JA consistently upregulated SlOR expression under both light and dark conditions (Figure S1). These results suggest that JA‐mediated regulation of carotenoid content is light‐dependent and may act through transcriptional modulation of carotenoid biosynthetic genes.

Ethylene is a key regulator of carotenoid biosynthesis in tomato fruits. To determine whether JA's effects involve ethylene signaling, we analyzed the expression of ethylene biosynthetic genes after MeJA application under both light and dark conditions. Consistent with Li et al. [42], *SlACS2* and *SlACO1* were markedly upregulated following MeJA treatment, irrespective of light exposure, with no change in regulatory direction (Figure S2). This indicates that MeJA's bidirectional regulation of carotenoid biosynthesis functions independently of the ethylene pathway, consistent with the findings of Liu et al. [27] that JA‐mediated carotenoid regulation is ethylene‐independent.

### 2.2 SlPIF1a is Crucial for the Reduction of Lycopene Content in the Dark

Since the effect of MeJA on carotenoid accumulation depends on light, and previous work by Alba et al. [15] showed that light regulates carotenoid biosynthesis independently of ethylene signaling, the bidirectional regulation by MeJA may arise from crosstalk between JA and light signaling pathways. *SlPIF1a* has been reported to negatively regulate carotenoid biosynthesis by repressing *SlPSY1* expression in a light‐dependent manner [24]. The PIF protein family generally acts as a negative regulator of light signaling by accumulating under low‐light conditions and suppressing the expression of downstream light‐responsive genes [43, 44]. To elucidate the role of SlPIF1a in carotenoid regulation, two *SlPIF1a* mutant lines (*Slpif1a‐1* and *Slpif1a‐2*) were generated using the CRISPR/Cas9 system. DNA sequencing confirmed early translation termination due to nucleotide deletions (Figure S3A–C), and off‐target analysis verified specific mutagenesis (Figure S3D). In parallel, two SlPIF1a‐FLAG overexpression lines (OE‐SlPIF1a‐1 and OE‐SlPIF1a‐2) were created under the control of the 35S promoter (Figure S3E). RT‐qPCR confirmed that *SlPIF1a* transcript levels were four‐ to five‐fold higher in these lines compared with the wild type (WT) (Figure S3F).

**FIGURE 2:**
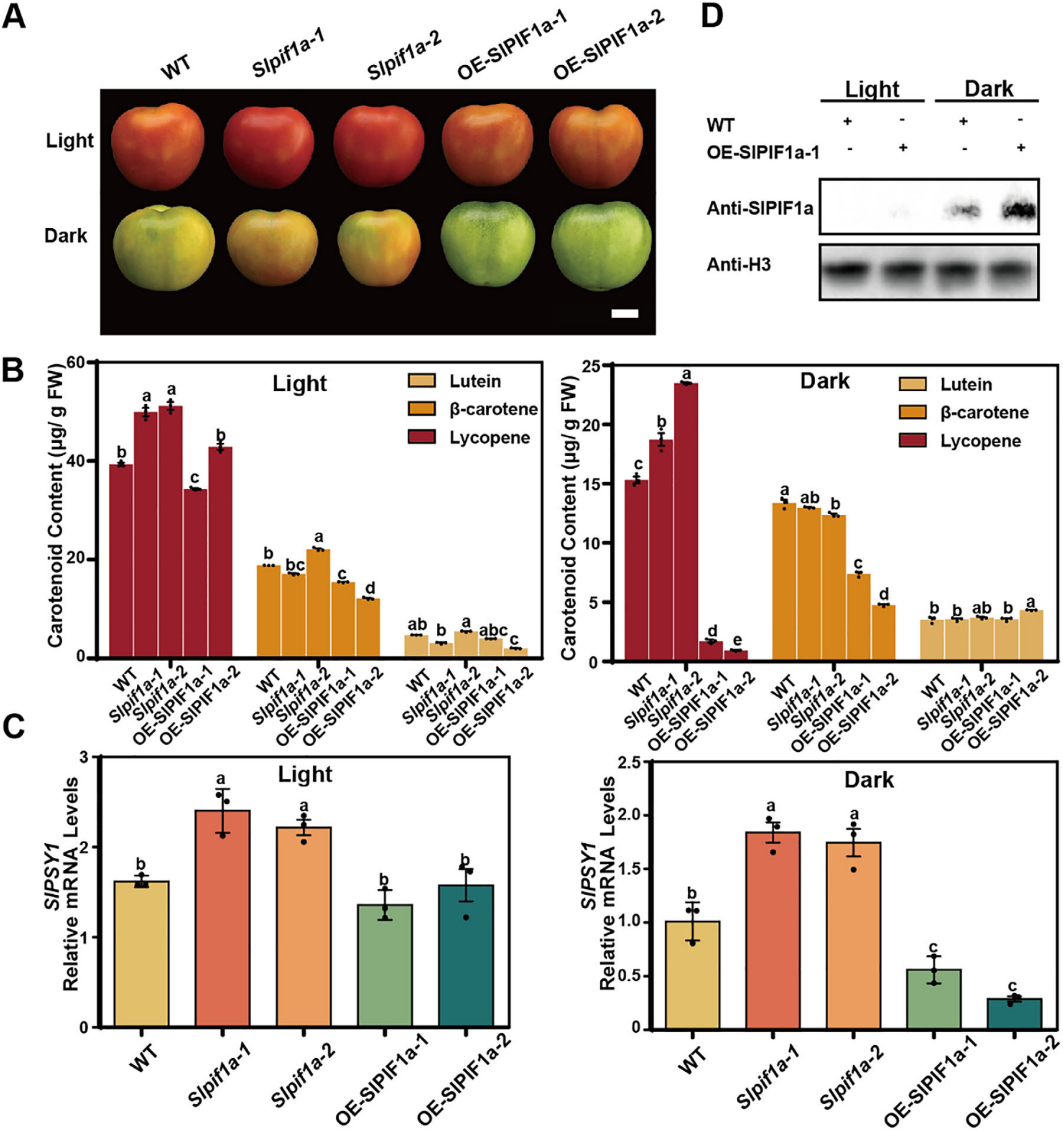
SlPIF1a negatively regulates carotenoid biosynthesis in tomato fruits. (A) The fruits of WT (Ailsa Craig), *SlPIF1a* knockout lines (*Slpif1a‐1*, *Slpif1a‐2*) and overexpression lines (OE‐SlPIF1a‐1, OE‐SlPIF1a‐2) were harvested at mature green stage (MG) and treated with aqueous solution of 0.2% ethanol for 5 days. Light treatment was consistent with Figure 1A. Scale bar = 1 cm. (B) Carotenoid concentrations in fruit pericarps of WT, *Slpif1a‐1, ‐2* and OE‐SlPIF1a‐1, ‐2 were measured. Each sample contained pericarps from three different fruits as a biological replicate. Three replicates were performed per experiment. Values represent means ± SE. Different lowercase letters were used to indicate statistically significance difference by two‐way ANOVA (*P*≤0.05, Tukey's multiple comparisons test). Source data and statistical summary can be found in the Supplement Data Set 1. (C) The mRNA levels of *SlPSY1* were determined with a method consistent with Figure 1G. Each sample contained pericarps from three different fruits as a biological replicate. Three replicates were performed per experiment. Values represent means ± SE. Different lowercase letters were used to indicate statistically significance difference (*p* ≤ 0.05) by one‐way ANOVA, Tukey's multiple comparisons test. Source data and statistical summary can be found in the Supplement Data Set 1. (D) The protein accumulation levels of SlPIF1a detected in the fruit pericarps of WT and OE‐SlPIF1a‐1 lines under light and dark conditions.

Lycopene, β‐carotene, and lutein contents in the fruits of *SlPIF1a* transgenic and WT lines were analyzed under both light and dark conditions. Under light, *Slpif1a* mutant lines exhibited significantly higher lycopene levels than the WT, whereas the overexpression lines showed no difference from the WT despite a fivefold increase in *SlPIF1a* transcript levels (Figure 2A,B; Figure S3F). Under dark conditions, lycopene levels remained higher in the *Slpif1a* mutants but were markedly lower in the overexpression lines compared with the WT (Figure 2A,B). These results suggest that light‐induced degradation of SlPIF1a may play a key regulatory role. To verify this, we measured SlPIF1a protein accumulation in WT and OE‐SlPIF1a‐1 lines under both light and dark conditions. The results confirmed that SlPIF1a protein levels were higher in OE‐SlPIF1a‐1 lines under darkness, while no detectable SlPIF1a protein was observed under light in either line (Figure 2D). The β‐carotene and lutein contents in the *SlPIF1a* transgenic lines were also quantified. Under both light and dark conditions, β‐carotene content in the overexpression lines was significantly lower than that in the WT, whereas no significant difference was detected between the *Slpif1a* mutants and WT. Furthermore, lutein content changes did not correlate with *SlPIF1a* expression levels (Figure 2B). Together, these results indicate that *SlPIF1a* primarily regulates lycopene accumulation in tomato fruits.

RT–qPCR analysis revealed that *SlPSY1* expression was higher in *Slpif1a* mutant lines than in the WT under both light and dark conditions, while it was significantly lower in overexpression lines only under darkness (Figure 2C). These findings indicate that SlPIF1a plays an important role in regulating lycopene accumulation under both light and dark conditions. To further explore the regulatory range of SlPIF1a, we examined the expression of other key carotenoid biosynthetic genes under dark conditions. The results showed that SlPIF1a negatively regulated *SlPDS* expression but had no significant effect on *SlDXS* or *SlGGPS* (Figure S4A–C). Additionally, the expression of the plastid formation gene *SlOR* was unaffected by SlPIF1a (Figure S4D). Collectively, these results demonstrate that the regulatory effect of SlPIF1a on carotenoid biosynthesis in tomato fruits mainly occurs at the initial stage of the carotenoid pathway—specifically, during the conversion of geranylgeranyl diphosphate (GGPP) to lycopene—rather than through modulation of the MEP pathway.

### 2.3 JA Regulated Lycopene Content Depending on SlPIF1a

**FIGURE 3:**
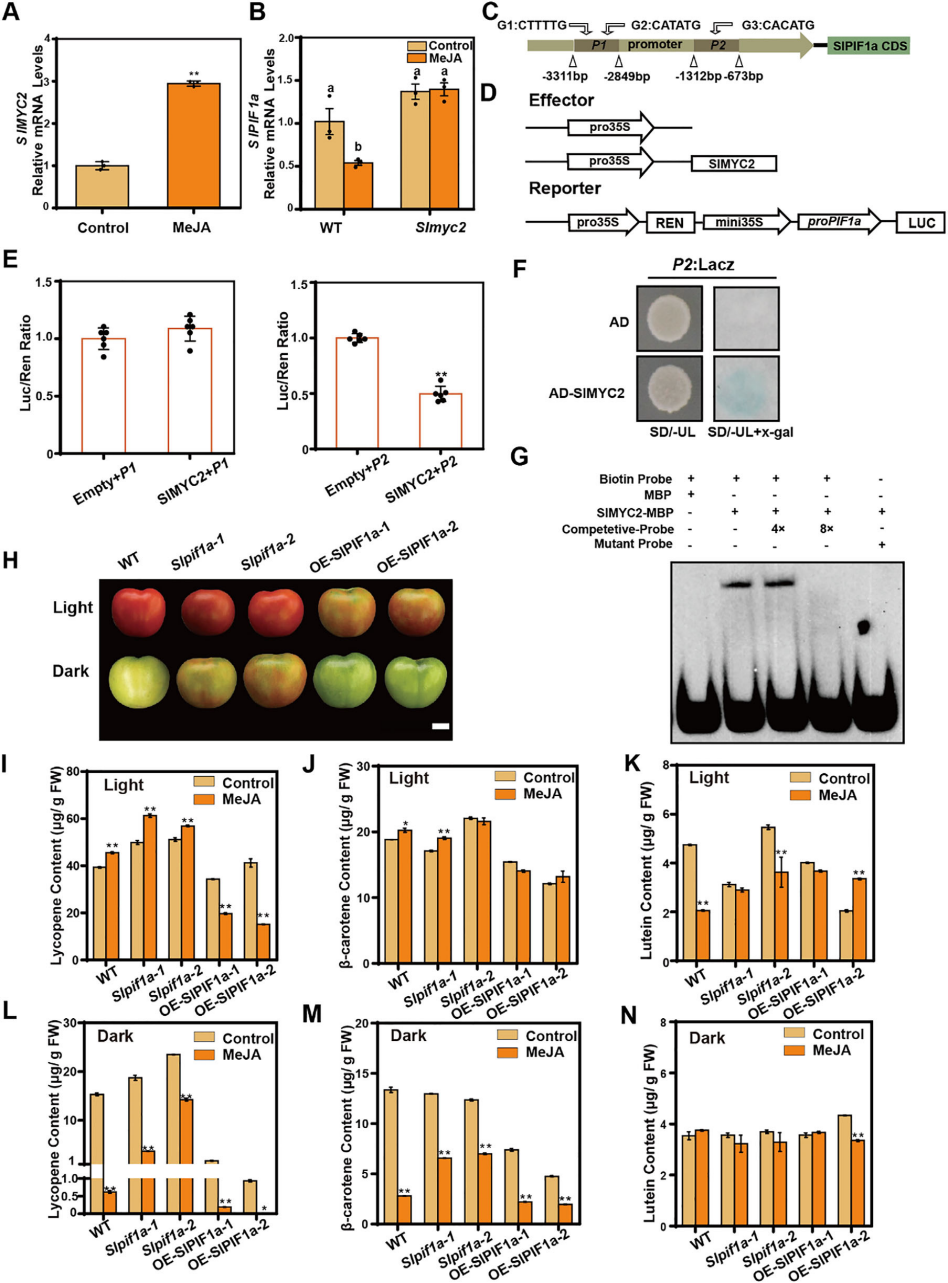
JA regulated the carotenoid contents mediated by SlPIF1a. The fruits of WT and *SlMYC2* knockout lines (*Slmyc2*) were harvested at MG stage and treated with JA for 3 h. The mRNA levels of *SlMYC2* in fruit pericarps of WT were determined (A). The mRNA levels of *SlPIF1a* in fruit pericarps of WT and *Slmyc2* lines were determined (B). Tomato housekeeping gene *SlUBQ* (Solyc01g056940) and *SlActin2* (Solyc11g005330) were used as internal control. Each sample contained pericarps from three different fruits as a biological replicate. Three replicates were performed per experiment. Asterisks indicate statistically significant differences analyzed by unpaired student's *t*‐test, two‐tailed (^**^ *p* ≤ 0.01). Different lowercase letters were used to indicate statistically significance difference (*p* ≤ 0.05) by two‐way ANOVA, Tukey's multiple comparisons test. Source data and statistical summary can be found in the Supplement Data Set 1. (C‐E) Transcriptional inhibition of SlMYC2 on the *SlPIF1a* promoter by Dual‐LUC system. The *SlMYC2* CDS was cloned into the effector vector (pGreen II 62‐SK) and *P1* and *P2* fragments were inserted into the reporter vector (pGreen II 0800 miniLUC). Vectors of effectors and reporters were co‐infiltrated into *Nicotiana benthamiana* leaves to analyze the activity ratio of LUC (firefly luciferase)/REN (renilla luciferase). Values represent means ± SE, *n* = 6. Unpaired student's *t*‐test, two‐tailed (^**^ *p* ≤ 0.01). Source data and statistical summary can be found in the Supplement Data Set 1. (F) Y1H assay showing that SlMYC2 bound to the promoter of *SlPIF1a* (*P2*). SD/‐UL, SD medium lacking Ura and Leu. X‐gal, 5‐Bromo‐4‐chloro‐3‐indolyl β‐D‐galactopyranoside. Blue plaques indicate interaction between protein and *SlPIF1a* promoter. (G) EMSA showing that SlMYC2 could directly bind to the promoter of *SlPIF1a*. EMSA experiments were conducted with a biotin‐labeled *SlPIF1a* promoter fragment containing a G‐box. An unlabeled version of the same *SlPIF1a* promoter fragment was used as a competitor at a 4‐fold and 8‐fold greater concentration than the labeled probe. MBP‐tagged SlMYC2 protein (2 µg) were purified. Purified MBP (2 µg) was used as a negative control. The mutant probe indicates the sequence of G‐box mutated to random sequence. (H) The fruits of WT, *SlPIF1a* knockout lines (*Slpif1a‐1*, *Slpif1a‐2*) and overexpression lines (OE‐SlPIF1a‐1, OE‐SlPIF1a‐2) were harvested at MG stage and treated with MeJA for 5 days. Storage condition for light and dark groups were the same as in Figure 2A. Scale bar = 1 cm. (I‐N) Lycopene, β‐carotene and lutein concentrations in fruit pericarps of WT, *Slpif1a‐1*, *‐2* and OE‐SlPIF1a‐1, ‐2 were measured with MeJA treatment under light (I‐K) and dark (L‐N). Each sample contained pericarps from three different fruits as a biological replicate. Three replicates were performed per experiment. The data were presented together with the results of Figure 2B. Values represent means ± SE. Asterisks indicate statistically significant differences analyzed by two‐way ANOVA (^*^ *p*≤0.05, ^**^ *p*≤0.01, Šídák's multiple comparisons test). Source data and statistical summary can be found in the Supplement Data Set 1.

To determine whether SlPIF1a mediates JA's bidirectional regulation of carotenoid biosynthesis, we first confirmed that *SlMYC2* expression in tomato fruits was upregulated by MeJA (Figure 3A). In contrast, *SlPIF1a* expression was significantly suppressed by MeJA in the WT but remained unchanged in the *Slmyc2* mutant line, previously identified as *SlMYC2‐KO‐3* by Liu et al. [45] (Figure 3B). Similar results were observed in the *jai1‐1* mutant, which carries a mutation in the JA receptor (Figure S5A). In *jai1‐1*, *SlPIF1a* transcript levels were notably higher than in the WT, and MeJA‐induced suppression was markedly reduced (Figure S5). Carotenoid analysis in *Slmyc2* fruits under different light conditions showed that, under light, all carotenoid levels were significantly lower than in the WT. Under dark conditions, lycopene content did not differ significantly, whereas β‐carotene and lutein were slightly lower (Figure S6A,B). These results suggest that JA's light‐dependent regulation of carotenoids involves the SlMYC2–SlPIF1a module.

MYC2 is a core transcription factor in the JA signaling pathway known to activate *PSY1* expression by binding to its promoter in orange [28]. To elucidate how MeJA influences *SlPIF1a*, we analyzed previously published ChIP‐seq data [46] and identified *SlPIF1a* as a direct target of SlMYC2. Three G‐boxes were detected within the 3.5 kb promoter region of *SlPIF1a*. These were divided into two fragments (P1 and P2) and inserted into the pGreenII 0800‐miniLUC vector, while the *SlMYC2* CDS was cloned into the pGreenII 62‐SK vector (Figure 3C). Dual‐luciferase reporter assays revealed that SlMYC2 suppressed LUC expression driven by the P2 promoter but not by P1, relative to the control (Figure 3D,E). Yeast one‐hybrid (Y1H) assays confirmed direct binding of SlMYC2 to the *SlPIF1a* promoter. The G‐box (CACATG) fragment from the *SlPIF1a* promoter was inserted into the pLACZi vector, and the *SlMYC2* CDS was cloned into pGAD424. Co‐expression in the YM4271 yeast strain demonstrated binding of SlMYC2 to the *SlPIF1a* promoter (Figure 3F), which was further verified by electrophoretic mobility shift assay (EMSA) (Figure 3G). Together, these data show that JA represses *SlPIF1a* transcription through direct targeting by SlMYC2.

To test whether SlPIF1a mediates JA's bidirectional regulation of lycopene biosynthesis, *SlPIF1a* transgenic and WT tomato fruits were treated with MeJA under light and dark conditions, and lycopene, β‐carotene, and lutein contents were measured. Under light, lycopene increased in both *Slpif1a* mutant and WT fruits but decreased markedly in *SlPIF1a* overexpression lines after MeJA treatment (Figure 2A and Figure 3H–I). In darkness, WT fruits showed a decline, while overexpression lines exhibited patterns similar to those under light (Figure 2B and Figure 3L). In *Slpif1a* mutants, lycopene levels also decreased after JA treatment, but the reduction was significantly weaker than in the WT (Figure 2B and Figure 3L), suggesting functional redundancy with other PIF proteins. β‐Carotene levels increased significantly under light in both WT and *Slpif1a‐1* fruits, whereas no significant changes were observed in other genotypes (Figure 3J,K). Under darkness, JA exhibited a general suppressive trend on β‐carotene levels across all lines. Lutein content showed no consistent pattern among genotypes (Figure 3M,N). Overall, these results indicate that SlPIF1a accumulation modulates JA's regulatory effects on lycopene biosynthesis.

### 2.4 SlPIF1a Repressed *SlPSY1* Expression Depending on SlMYC2

A previous study reported that *SlPIF1a* binds to the PBE‐box in the *SlPSY1* promoter, but direct repression of *SlPSY1* expression has not been demonstrated [24]. To test this, we first confirmed that SlPIF1a localizes to the nucleus (Figure S7A). Dual‐luciferase assays were then performed using *Nicotiana benthamiana* leaves to investigate the regulatory mechanism. The effector construct contained the *SlPIF1a* CDS driven by the 35S promoter, and the reporter construct carried the *SlPSY1* promoter fragment containing the PBE‐box fused to the *LUC* gene. Unexpectedly, LUC activity driven by the *SlPSY1* promoter showed no difference when co‐expressed with SlPIF1a compared to the control (Figure S7B). To exclude the possibility that SlPIF1a degradation affected the result, western blot analysis confirmed successful expression and accumulation of SlPIF1a in *N. benthamiana* leaves (Figure S7C). These findings contrasted with our RT‐qPCR data (Figure 2C), where *SlPSY1* expression was higher in *Slpif1a* mutants than in the WT, suggesting that another protein may cooperate with SlPIF1a to repress carotenoid biosynthesis.

**FIGURE 4:**
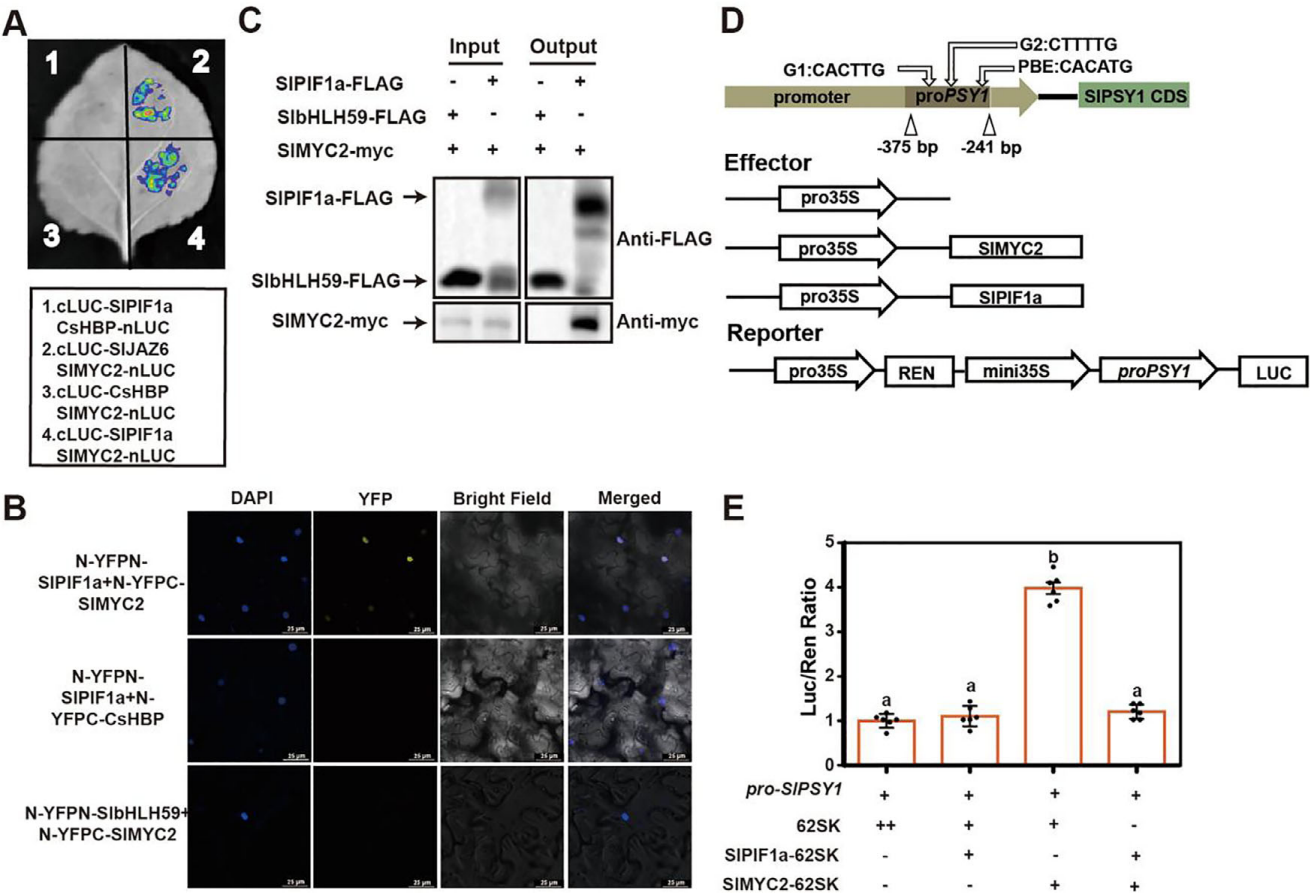
SlPIF1a interacted with SlMYC2 and interfered with its activation on *SlPSY1* promoter. (A) LCI assay showing the interaction between SlPIF1a and SlMYC2. Experimental group is Group 4 with Group 1 and Group 3 acting as negative controls and Group 2 acting as positive control. (B) BiFC assay showing that SlPIF1a interacts with SlMYC2, revealed as fluorescence in the nucleus. N‐YFPC‐CsHBP + N‐YFPN‐SlPIF1a and N‐YFPC‐SlMYC2 + N‐YFPN‐SlbHLH59 combinations were used as negative controls. Scale bar = 25 µm. (C) Co‐IP assay demonstrating the interaction between SlPIF1a and SlMYC2. myc‐tagged SlMYC2 and FLAG‐tagged SlPIF1a were co‐overexpressed in *N.benthamiana* leaves. Anti‐GFP magnetic beads were used for immunoprecipitation. Anti‐FLAG and anti‐myc antibodies were used for immunoblotting analysis. Co‐overexpression of SlbHLH59‐FLAG and SlMYC2‐myc was used as a negative control. The band detected by the anti‐myc antibody in the IP samples indicates an interaction between SlPIF1a and SlMYC2. ‘+’ and ‘−’ respectively represent the presence or absence of corresponding plasmids in experimental *N.benthamiana* leaves. (D‐E) Dual‐LUC assay showing the effect of SlPIF1a and SlMYC2 on the promoter activity of *SlPSY1*. The promoter activity of *SlPSY1* was indicated by the activity ratio of LUC/REN. ‘+’ and ‘−’ respectively represent the presence or absence of corresponding plasmids in experimental *N.benthamiana leaves*. Data are presented as means ± SE, *n* = 6. Different lowercase letters were used to indicate statistically significance difference (*p* ≤ 0.05) by one‐way ANOVA, Tukey's multiple comparisons test. Source data and statistical summary can be found in the Supplement Data Set 1.

Since PIF4 interacts with MYC2 in *Arabidopsis* [47], we investigated whether SlMYC2 interacts with SlPIF1a in regulating carotenoid biosynthesis. Luciferase complementation imaging (LCI) assays confirmed their interaction (Figure 4A), which was further validated by bimolecular fluorescence complementation (BiFC) and co‐immunoprecipitation (Co‐IP) assays (Figure 4B,C).

To further assess how SlPIF1a and SlMYC2 affect *SlPSY1* expression, a dual‐LUC assay was conducted by co‐expressing SlPIF1a, SlMYC2, and the *SlPSY1* promoter in tobacco leaves. The results showed that SlMYC2 strongly activated *SlPSY1* expression, whereas *SlPIF1a* abolished this activation. However, neither *SlPIF1a* alone nor co‐expression of *SlMYC2* and *SlPIF1a* reduced LUC activity compared with the control (Figure 4D,E). These findings indicate that SlPIF1a represses *SlPSY1* expression by interacting with SlMYC2 and interfering with its transcriptional activation of *SlPSY1*.

### 2.5 N‐Terminal Domain of SlPIF1a Interacted With SlMYC2 and Played the Leading Role in Interfering With the Activation of *SlPSY1*

**FIGURE 5:**
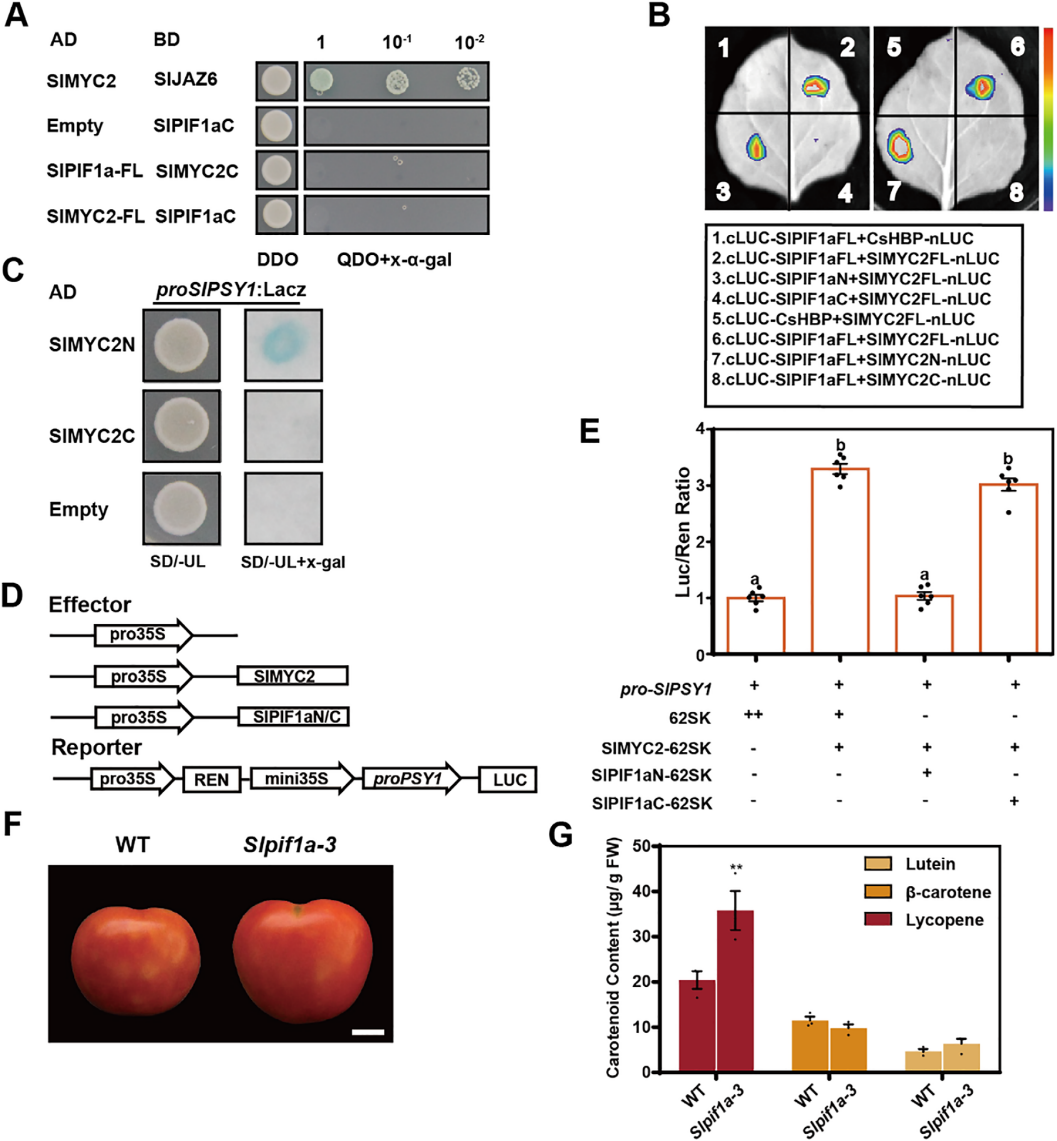
N‐terminal side of SlPIF1a interacted with SlMYC2 and interfered with the activation of *SlPSY1*. Y2H assay showing the interaction fragments between SlPIF1a and SlMYC2. Experimental group is SlPIF1a‐AD + SlMYC2C‐BD and SlMYC2‐AD + SlPIF1aC‐BD with AD + SlPIF1aC‐BD acting as negative controls and SlMYC2‐AD + SlJAZ6‐BD acting as positive control. SlMYC2C means C‐terminal of SlMYC2 and SlPIF1aC means C‐terminal of SlPIF1a. DDO, SD medium lacking Trp and Leu; QDO, SD medium lacking Trp, Leu, His, and Ade; X‐α‐gal, 5‐Bromo‐4‐chloro‐3‐indoxyl‐α‐D‐galactopyranoside. Blue plaques indicate interaction between two proteins. (B) LCI assay showing the interaction fragments between SlPIF1a and SlMYC2. Experimental group is Group 3,4,7 and 8 with Group 1 and 5 acting as negative controls and Group 2 and 6 acting as positive controls. SlMYC2N means N‐terminal of SlMYC2 and SlPIF1aN means N‐terminal of SlPIF1a. (C) Y1H assay showing that N‐terminal of SlMYC2 bound to the promoter of *SlPSY1*. SD/‐UL, SD medium lacking Ura and Leu. X‐gal, 5‐Bromo‐4‐chloro‐3‐indolyl β‐D‐galactopyranoside. Blue plaques indicate interaction between protein and *SlPSY1* promoter. (D‐E) Dual‐LUC assay showing the effect of interaction between SlPIF1a fragments and SlMYC2 on the promoter activity of *SlPSY1*. The promoter activity of *SlPSY1* was indicated by the activity ratio of LUC /REN. ‘+’ and ‘−’ respectively represent the presence or absence of corresponding plasmids in experimental *N. benthamiana* leaves. Data are presented as means ± SE, *n* = 6. Different lowercase letters were used to indicate statistically significance difference (*p* ≤ 0.05) by one‐way ANOVA, Tukey's multiple comparisons test. Source data and statistical summary can be found in the Supplement Data Set 1. (F) The fruits of WT and *SlPIF1aN* knockout lines (*Slpif1a‐3*) were harvested at mature green stage and treated with water for 5 days stored at 24°C under 16 h light and 8 h dark conditions. Scale bar = 1 cm. (G) Lycopene, β‐carotene and lutein concentrations in fruit pericarps of WT and *Slpif1a‐3* were measured. Each sample contained pericarps from three different fruits as a biological replicate. Three replicates were performed per experiment. Values represent means ± SE. Asterisks indicate statistically significant differences analyzed by two‐way ANOVA (^*^ *p*≤0.05, Šídák's multiple comparisons test). Source data and statistical summary can be found in the Supplement Data Set 1.

To elucidate how SlPIF1a interferes with the activation of SlMYC2 on *SlPSY1*, we hypothesized that the region of SlMYC2 interacting with SlPIF1a corresponds to the same domain responsible for activating *SlPSY1* expression, suggesting a competitive interaction between these elements. To identify the specific interacting regions, both SlPIF1a and SlMYC2 were divided into N‐terminal and C‐terminal fragments, which were individually cloned into the pGBKT7 vector and tested for self‐activation. The N‐terminal regions of both proteins exhibited transcriptional activation activity (Figure S8). Yeast two‐hybrid (Y2H) assays were performed to test interactions between the C‐terminal region of SlPIF1a (SlPIF1aC) and full‐length SlMYC2, as well as between the C‐terminal region of SlMYC2 (SlMYC2C) and full‐length SlPIF1a. Neither SlPIF1aC interacted with SlMYC2 nor SlMYC2C with SlPIF1a, indicating that the interaction sites between these proteins are located in their N‐terminal regions (Figure 5A). An LCI experiment confirmed this finding: fluorescence signals were detected only when SlPIF1aN (the N‐terminal region of SlPIF1a) was co‐expressed with SlMYC2, or when SlMYC2N (the N‐terminal region of SlMYC2) was co‐expressed with SlPIF1a, in tobacco leaves (Figure 5B).

The Y1H analysis was used to determine whether SlMYC2N binds to the *SlPSY1* promoter. As expected, SlMYC2N bound directly to the *SlPSY1* promoter, while SlMYC2C did not (Figure 5C). These results suggest that SlPIF1aN interacts with the binding domain of SlMYC2, which normally associates with the *SlPSY1* promoter, thereby repressing SlMYC2 binding. A dual‐luciferase (Dual‐LUC) assay was conducted by co‐expressing SlMYC2 and the *SlPSY1* promoter with either SlPIF1aN or SlPIF1aC in tobacco leaves. The results showed that SlPIF1aN repressed SlMYC2‐mediated activation of *SlPSY1*, whereas SlPIF1aC did not (Figure 5D,E).

Among the *Slpif1a* mutants obtained, a unique line (*Slpif1a‐3*) contained an N‐terminal mutation involving a seven‐nucleotide deletion at position 46 and a one‐nucleotide insertion at position 175, resulting in a frameshift that abolished SlPIF1aN function (Figure S9A–C). Off‐target analyses confirmed the specificity and reliability of the CRISPR/Cas9‐induced mutation (Figure S9D). LCI assays showed that the truncated N‐terminal region of SlPIF1a in *Slpif1a‐3* could no longer interact with SlMYC2 (Figure S10C). Fruits from *Slpif1a‐3* lines contained significantly higher lycopene levels than the WT, whereas β‐carotene and lutein contents were unchanged (Figure 5G). This phenotype supports the hypothesis that the interaction between SlPIF1aN and SlMYC2 interferes with SlMYC2‐mediated activation of *SlPSY1* transcription.

These results clarify the regulatory mechanism of JA on lycopene biosynthesis through SlPIF1a under light conditions: in MeJA‐treated tomato fruits, activated SlMYC2 suppresses *SlPIF1a* expression, thereby preventing interference with *SlPSY1* activation.

### 2.6 Co‐Accumulation of SlPIF1a and SlNATA1 Dramatically Repressed Lycopene Biosynthesis

Interestingly, after MeJA treatment, lycopene content in *Slpif1a‐3* fruits decreased significantly, consistent with the reduction observed in WT fruits treated with MeJA in the dark (Figure S10A,B). To examine this effect, we analyzed the protein structure of *Slpif1a‐3* and found that the APB domain—responsible for SlPIF1a ubiquitination and degradation through interaction with phyB—was disrupted in the *Slpif1a‐3* protein [48, 49]. A recent study demonstrated that light induces a conformational change in the phytochrome‐specific (PHY) domain of phyB, converting a β‐sheet to an α‐helix and thereby promoting its interaction with the APB domain of PIF6 [50]. We hypothesized that the APB domain mutation caused SlPIF1a overaccumulation in *Slpif1a‐3* lines, altering MeJA‐induced inhibition of lycopene biosynthesis. To verify this hypothesis, fruits from WT, *Slpif1a‐1*, and *Slpif1a‐3* lines were stored under light and dark conditions, and SlPIF1a protein levels were measured. In the WT, SlPIF1a protein accumulated markedly in darkness compared with light. In *Slpif1a‐1*, SlPIF1a was undetectable under both conditions, whereas in *Slpif1a‐3*, SlPIF1a accumulated regardless of light exposure (Figure S10D). These findings indicate that the *Slpif1a‐3* form of SlPIF1a cannot be ubiquitinated under light, supporting our hypothesis that SlPIF1a accumulation alters JA‐mediated regulation of lycopene biosynthesis.

**FIGURE 6:**
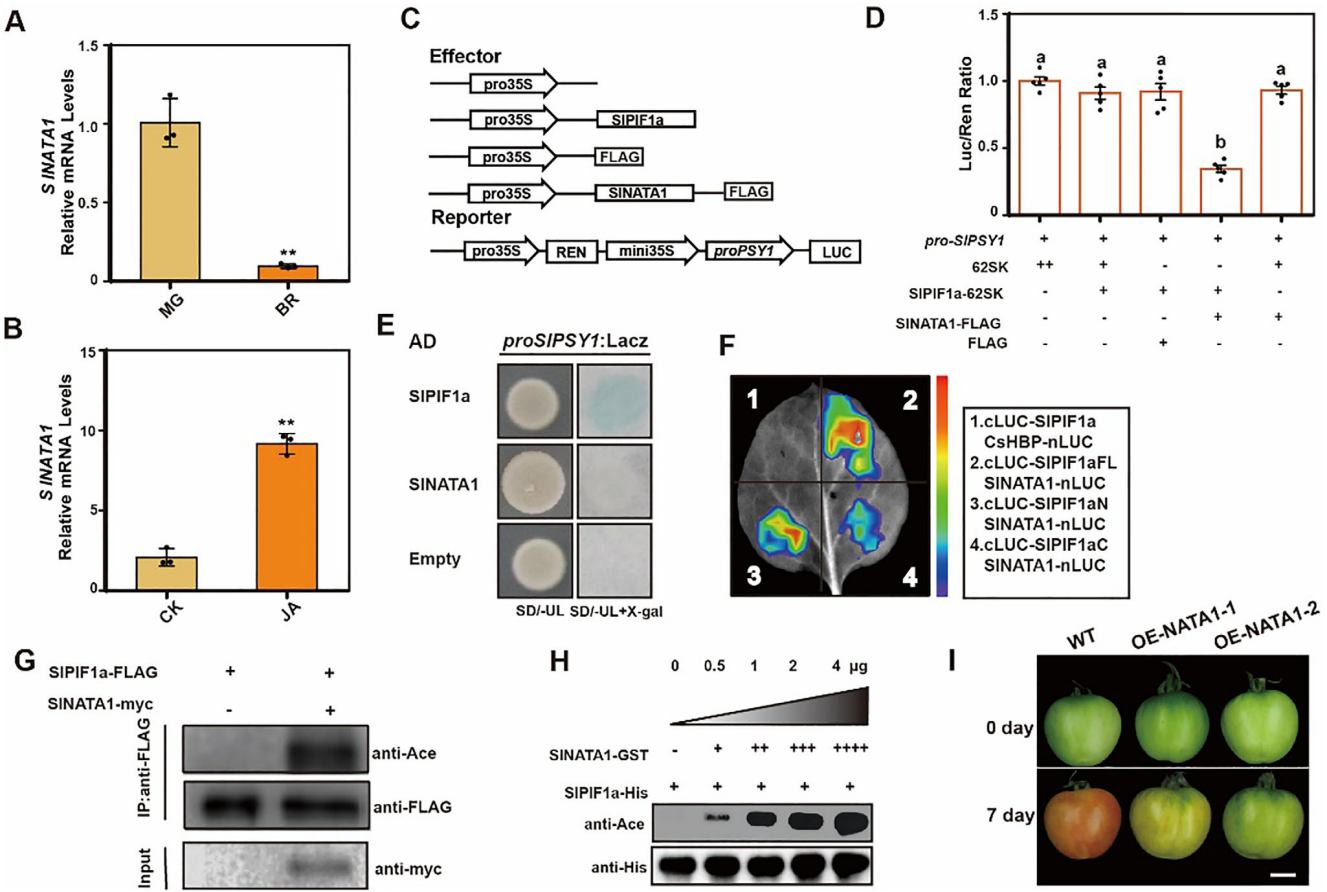
SlNATA1 repressed the carotenoid biosynthesis by interacting with and acetylating SlPIF1a. (A) The mRNA levels of *SlNATA1* in MG stage and BR (the breaker stage) fruit pericarps were determined. Each sample contained pericarps from three different fruits as a biological replicate. Three replicates were performed per experiment. Values represent means ± SE. Unpaired student's *t*‐test, two‐tailed (^**^ *p* ≤ 0.01). (B) The fruits of WT lines were harvested at MG and treated with MeJA for 3 h. The mRNA levels of *SlNATA1* in fruit pericarps were determined. Tomato housekeeping gene *SlUBQ* (Solyc01g056940) and *SlActin2* (Solyc11g005330) were used as internal control. Each sample contained pericarps from three different fruits as a biological replicate. Three replicates were performed per experiment. Values represent means ± SE. Unpaired student's *t*‐test, two‐tailed (^**^ *p* ≤ 0.01). (C‐D) Dual‐LUC assay showing the effect of SlPIF1a and SlNATA1 on the promoter activity of *SlPSY1*. The promoter activity of *SlPSY1* was indicated by the activity ratio of LUC /REN. ‘+’ and ‘−’ respectively represent the presence or absence of corresponding plasmids in experimental *N. benthamiana* leaves. Data are presented as means ± SE, n = 6. Different lowercase letters were used to indicate statistically significance difference (*P* ≤ 0.05) by one‐way ANOVA, Tukey's multiple comparisons test. Source data and statistical summary can be found in the Supplement Data Set 1. (E) Y1H assay showing that SlPIF1a bound to the promoter of *SlPSY1* but SlNATA1 did not. SD/‐UL, SD medium lacking Ura and Leu. X‐gal, 5‐Bromo‐4‐chloro‐3‐indolyl β‐D‐galactopyranoside. Blue plaques indicate interaction between protein and *SlPSY1* promoter. (F) LCI assay showing the interaction fragments between SlPIF1a and SlNATA1. Experimental group is Group 2,3,4 and with Group 1 acting as a negative control. (G) In vivo acetylation of SlPIF1a by SlNATA1. SlPIF1a‐FLAG and SlNATA1‐myc were co‐overexpressed in *N. benthamiana* leaves. Anti‐FLAG beads were used to immunoprecipitate the SlPIF1a protein to detect acetylation levels. Overexpression of *SlPIF1a* alone was used as a negative control. Anti‐FLAG and anti‐myc antibodies were used to indicate the protein loading of SlPIF1a‐FLAG and SlNATA1‐myc. (H) The acetylation levels of *SlPIF1a* increased with a rise in SlNATA1 protein concentration. SlPIF1a‐His protein purified from *E. coli* were mixed with increasing concentrations of SlNATA1‐GST protein purified from *E. coli*, incubated at 30°C for 60 min. (I) The fruits of WT and *SlNATA1* overexpression lines (OE‐NATA1‐1 and OE‐NATA1‐2) were harvested at mature green stage and stored at 24°C under dark conditions for 7 days. Scale bar = 1 cm.

To further investigate the negative regulation of JA on SlPIF1a accumulation, we performed Y2H screening to identify proteins interacting with SlPIF1a. Among 38 identified candidates, one encoded an acetyltransferase previously reported to respond to MeJA and confer resistance to clubroot disease in *Arabidopsis* [51]. RT‐qPCR analysis showed that *SlNATA1* expression declined sharply during tomato fruit ripening (Figure 6A) but was strongly induced by MeJA treatment (Figure 6B). Dual‐LUC assays revealed that co‐expression of SlPIF1a, SlNATA1, and the *SlPSY1* promoter in *N. benthamiana* leaves significantly repressed *SlPSY1* expression, whereas co‐expression of SlNATA1 and the *SlPSY1* promoter without SlPIF1a showed no repression (Figure 6C,D). Y1H assays further demonstrated that SlPIF1a bound directly to the *SlPSY1* promoter, while SlNATA1 did not (Figure 6E). These results indicate that the SlPIF1a–SlNATA1 module mediates the negative regulation of MeJA on lycopene biosynthesis.

### 2.7 SlNATA1 Negatively Regulates Lycopene Biosynthesis by Acetylating SlPIF1a

The LCI assays confirmed the physical interaction between SlPIF1a and SlNATA1, showing that both SlPIF1aN and SlPIF1aC could interact with SlNATA1 (Figure 6E,F). Given that SlNATA1 functions as an acetyltransferase, we hypothesized that it represses *SlPSY1* expression by acetylating SlPIF1a.

To test this, SlPIF1a tagged with FLAG (SlPIF1a‐FLAG) and SlNATA1 tagged with MYC (SlNATA1‐MYC) were co‐expressed in N. benthamiana leaves, with SlPIF1a‐FLAG alone serving as a control. Co‐expression with SlNATA1 markedly increased the acetylation level of SlPIF1a compared with the control, while total SlPIF1a protein remained constant (Figure 6G). The in vitro acetylation assays supported these findings. SlPIF1a‐His and SlNATA1‐GST fusion proteins were purified from a prokaryotic expression system. Increasing concentrations of SlNATA1‐GST led to elevated acetylation levels of SlPIF1a‐His, confirming that SlNATA1 acetylates SlPIF1a (Figure 6H). In addition, under both light and dark conditions, no significant effect of MeJA on SlPIF1a protein abundance was detected in tomato fruits, further indicating that MeJA likely regulates SlPIF1a function through acetylation rather than by modulating its protein accumulation (Figure S11A). To determine the role of SlNATA1 in lycopene biosynthesis, overexpression lines (OE‐NATA1‐1 and OE‐NATA1‐2) were generated under the 35S promoter (Figure S11A). RT‐qPCR confirmed that SlNATA1 expression was 13‐fold and 45‐fold higher in these lines compared with the WT (Figure S11B). Western blot analysis verified successful accumulation of SlNATA1‐FLAG protein in both overexpression lines (Figure S11C). We first examined the changes in SlNATA1 protein levels in OE‐NATA1‐1 plants before and after MeJA treatment. The results showed that MeJA promoted the accumulation of SlNATA1 protein in tomato plants under both light and dark conditions (Figure S11B). Fruit pigmentation in SlNATA1 overexpression lines progressed significantly slower than in WT fruits (Figure 6I), indicating that SlNATA1 negatively regulates lycopene biosynthesis in tomato fruits under dark conditions. Moreover, fruit ripening in the overexpression lines was markedly delayed relative to the WT under natural conditions (Figure S12). To further clarify the hierarchical relationship between the SlPIF1a‐SlNATA1‐SlPSY1 pathway and the SlMYC2‐SlPSY1 pathway, we examined changes in *SlPSY1* promoter activity when the three proteins were co‐expressed with *SlPSY1* promoter in a tobacco transient expression system. The results showed that, when all three proteins were present simultaneously, *SlPSY1* promoter activity was significantly reduced, which was consistent with the effect observed in the SlPIF1a and SlNATA1 only group. These results further suggest that, under dark conditions, MeJA preferentially activates the SlPIF1a‐SlNATA1‐mediated repressive pathway regulating *SlPSY1* expression (Figure S14).

**FIGURE 7:**
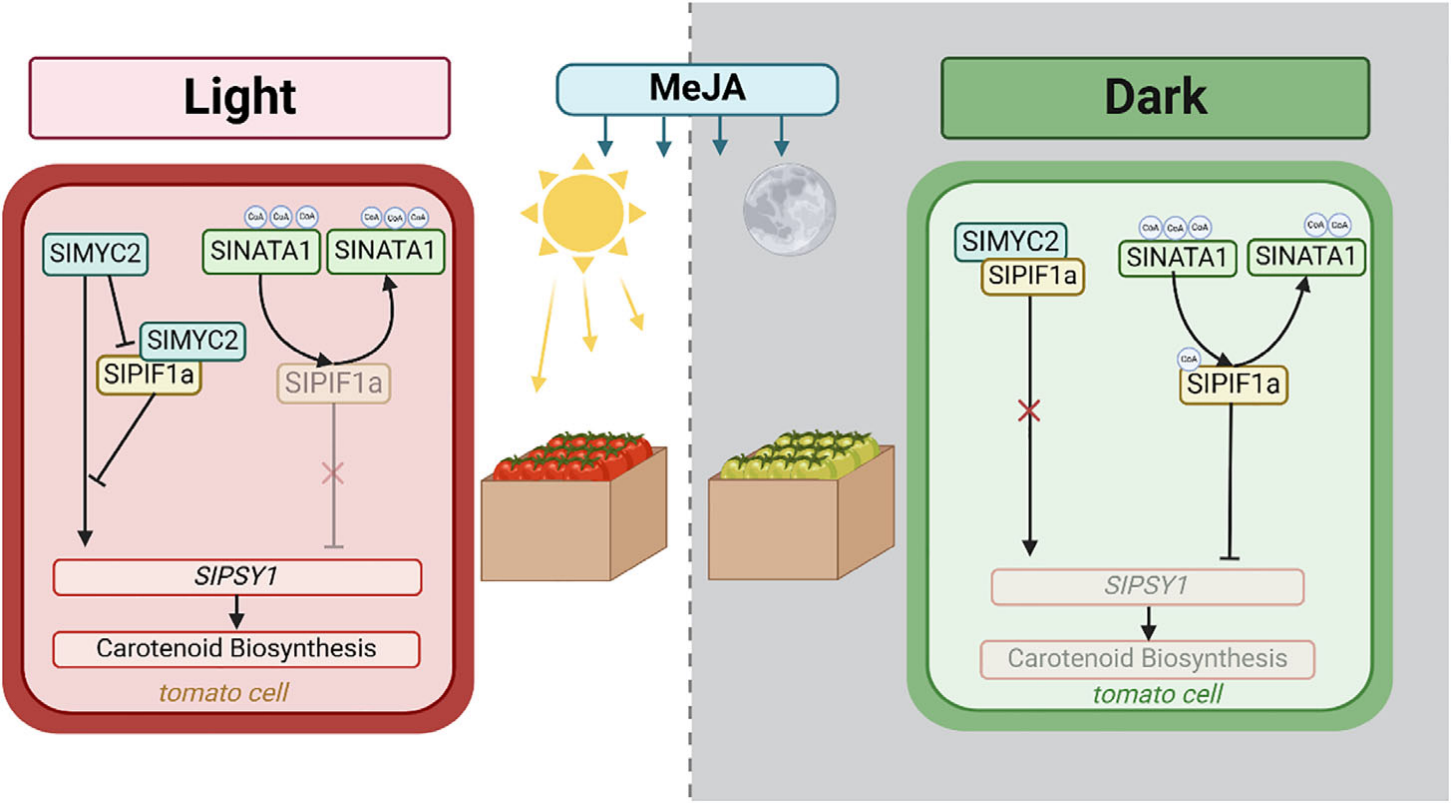
Mechanism model of JA's dual regulation on lycopene biosynthesis under different light conditions. In light, JA activated SlMYC2 to inhibit the expression of *SlPIF1a* and activate *SlPSY1* expression. In dark condition, exogenous JA induced the expression of acetylase *SlNATA1*, which repressed *SlPSY1* expression by acetylating the dark induced accumulated SlPIF1a.

Collectively, our study demonstrates that JA regulates lycopene biosynthesis through SlPIF1a in a light‐dependent manner. Under light conditions, JA activates SlMYC2, which represses *SlPIF1a* expression and promotes *SlPSY1* activation. Under dark conditions, exogenous JA induces the expression of the acetyltransferase *SlNATA1*, which represses *SlPSY1* by acetylating dark‐induced SlPIF1a accumulation (Figure 7). These findings highlight that light exposure should be carefully considered when applying JA as a postharvest ripening agent.

## 3 Discussion

Carotenoids are essential pigments and vital nutrients in plant fruits [52, 53]. Numerous studies have explored the regulatory role of JA in fruit coloration. Unlike previous findings, our results demonstrate that JA regulates lycopene biosynthesis in a bidirectional, light‐dependent manner. Under light conditions, JA promotes carotenoid biosynthesis by directly activating *SlPSY1* expression through the SlMYC2 transcription factor and inhibiting *SlPIF1a* expression, thereby reducing the interference of SlPIF1a with SlMYC2. In darkness, SlPIF1a protein accumulates significantly. MeJA‐induced acetyltransferase SlNATA1 acetylates SlPIF1a, altering its transcriptional activity and strongly repressing *SlPSY1* expression, ultimately suppressing carotenoid synthesis in tomato fruits.

### 3.1 The Regulatory Role of JA in Lycopene Synthesis is Bidirectional

JA is an important plant signaling molecule known to regulate carotenoid biosynthesis. Early studies reported that postharvest fumigation of tomato fruits with MeJA markedly enhanced lycopene synthesis [30]. Similarly, exogenous JA spraying on green‐mature fruits significantly increased lycopene accumulation compared with controls, whereas JA‐deficient mutants (*spr2* and *def1*) exhibited almost 40% lower lycopene content than the WT [27]. Our study indicates that JA's regulation of lycopene synthesis is bidirectional and influenced by light. Under light, JA promotes lycopene biosynthesis, while under darkness, JA strongly inhibits it (Figure 1A–D). RT–qPCR analysis revealed that among the carotenoid biosynthetic genes, *SlPSY1* displayed the most pronounced opposite expression trends in response to JA under light and dark conditions (Figure 1G). Genes encoding enzymes responsible for converting geranylgeranyl diphosphate (GGPP) to lycopene showed similar behavior. This may explain why Saniewski and Czapski [29] observed reduced lycopene accumulation when MeJA was applied in lanolin—the lanolin likely reduced light permeability on the tomato surface, attenuating JA's promotive effect. Furthermore, expression of *SlOR*, a key gene in plastid biogenesis, was unaffected by JA under light or darkness, indicating that JA's light‐dependent regulation of carotenoids does not occur through *SlOR*. Overall, JA's promotive effect on carotenoid accumulation likely acts at multiple stages of carotenoid biosynthesis and storage. However, its light‐dependent bidirectional regulation primarily targets lycopene biosynthesis during the GGPP‐to‐lycopene conversion phase.

Auxin provides a useful analogy: it exhibits concentration‐dependent dual effects, promoting growth at low levels but inhibiting it at high levels through differential regulation of IAA repressors and ARF transcription factors [54, 55]. Similarly, our results reveal that JA exhibits condition‐dependent (light‐dependent) bidirectional regulation of carotenoid synthesis. This concept may help explain the frequent contradictions observed in hormonal response studies, suggesting that plant hormones may generally display context‐dependent bidirectional activity.

From an applied perspective, this discovery provides valuable guidance for postharvest fruit handling. As a natural, environmentally safe plant hormone, JA offers advantages for ripening regulation compared with ethylene. Our findings support a bidirectional regulatory strategy in which environmental light conditions can be used to modulate the effects of exogenous JA on lycopene biosynthesis. This approach could overcome challenges in conventional ethylene‐based ripening methods, such as over‐ripening or uneven coloration caused by uncontrolled exposure times or concentrations. Moreover, JA offers dual benefits: it can mitigate chilling injury during postharvest transport and enhance resistance to fungal infection [56], addressing the reduced disease resistance often associated with ethylene treatments due to antagonistic interactions between the ethylene and JA signaling pathways [57].

### 3.2 The Accumulation and Acetylation of SlPIF1a Protein Significantly Inhibit Lycopene Synthesis

Darkness and far‐red light inhibit carotenoid accumulation in developing tomato fruits, likely through increased *phyA* transcription and mechanisms independent of ethylene signaling [58, 59]. PIF transcription factors are negative regulators of light signaling and are ubiquitinated and degraded via phytochrome‐mediated pathways. Previous studies reported that SlPIF1a, a negative regulator of carotenoid biosynthesis, binds to the *SlPSY1* promoter to repress its expression in tomato fruits [24]. In *Arabidopsis* seedlings, PIF1 also directly binds to the *PSY* promoter, reducing carotenoid accumulation [60]. Our results confirm that in MG+5 stage tomato fruits, carotenoid content was significantly higher in *Slpif1a* knockout lines than in the WT (Figure 2A–C), consistent with elevated *SlPSY1* expression. This supports the role of SlPIF1a as a negative regulator of carotenoid biosynthesis via *SlPSY1*. However, transient overexpression of SlPIF1a in tobacco did not suppress LUC activity driven by the *SlPSY1* promoter, implying that additional cofactors are required for effective repression in tomato [24].

However, surprisingly, transient overexpression of *SlPIF1a* in the tobacco system did not inhibit LUC reporter activity driven by the *SlPSY1* promoter, suggesting that additional proteins may be required to co‐regulate *SlPSY1* expression in tomato. Experimental results indicated that SlPIF1a negatively regulates *SlPSY1* expression in tomato fruits through two distinct pathways. Under light conditions, SlPIF1a interacts with SlMYC2, thereby interfering with SlMYC2‐mediated activation of *SlPSY1* (Figure 4A–E). This interaction occurs through the N‐terminal region of SlPIF1a, as evidenced by the higher lycopene content observed in *SlPIF1a* N‐terminal mutants compared with WT plants (Figure 5), confirming the inhibitory function of SlPIF1a. When both the acetyltransferase SlNATA1 and SlPIF1a are abundant, SlNATA1 acetylates SlPIF1a, further suppressing *SlPSY1* expression (Figure 6D–H). This repression coincides with the enhanced lycopene levels detected in *Slpif1a* mutant fruits. Nonetheless, no significant difference in lycopene content was found between SlPIF1a‐overexpression and WT fruits under light conditions (Figure 2A,B). This result contradicts previous findings from transient SlPIF1a‐GFP overexpression in tomato fruits [24]. The inconsistency may be attributed to differences in SlPIF1a protein accumulation between transient and stable overexpression systems.

In tomato, *SlPIF1a*, *SlPIF1b*, *SlPIF3*, and *SlPIF4* exhibit high expression in fruit tissues [61]. During fruit ripening, *SlPIF1a* expression increases, whereas *SlPIF3* expression decreases after the breaker stage. Previous studies have shown that SlPIF3 regulates geranylgeranyl diphosphate reductase (*SlGGDR*) involved in tocopherol biosynthesis [62]. Tocopherols are mainly synthesized at the mature green stage of fruit development; after the breaker stage, *SlGGDR* expression is repressed, *SlPSY1* is activated, tocopherol biosynthesis slows down, and carotenoid biosynthesis is initiated. These developmental patterns are consistent with the expression dynamics of SlPIF1a and SlPIF3. SlPIF1a and SlPIF3 may act as major regulators that coordinately control the synthesis and accumulation of secondary metabolites in tomato fruits. In addition, SlPIF1b shares high sequence homology with SlPIF1a, but loss of the APB domain in SlPIF1b leads to light‐independent protein stability [24, 63]. By contrast, carotenoid content is significantly increased in SlPIF4‐silenced lines, accompanied by a marked upregulation of *SlPSY1* and *SlGGPS2* at 6 days after the breaker stage [64]. This suggests functional redundancy between SlPIF4 and SlPIF1a, with SlPIF4 potentially acting as a compensatory gene in SlPIF1a knockout backgrounds.

Our previous studies demonstrated that SlPIF1a promotes fruit development in darkness through endoreduplication [65]. In the present research, we further elucidate the SlPIF1a‐mediated regulatory mechanism underlying light‐dependent carotenoid biosynthesis. Previously, we reported that in the *Slpif1a‐1* knockout line, reduced cell ploidy resulted in smaller cells compared with the WT, whereas in the *Slpif1a‐3* line [designated *Slpif1a* (*–7 + 1*) in the original study], sustained SlPIF1a accumulation led to increased cell ploidy and larger cell size [65]. In the current study, we found that carotenoid contents in mature fruits of both *Slpif1a‐1* and *Slpif1a‐3* lines were significantly higher than those of the WT (Figures 2B and 5G). These findings indicate that altered carotenoid accumulation in SlPIF1a transgenic fruits is unlikely to result from changes in cell ploidy. Instead, our results reveal a dynamic regulatory mechanism in which light signals modulate SlPIF1a protein abundance, coordinating energy allocation between two independent pathways—fruit growth and secondary‐metabolite biosynthesis.

### 3.3 The Direction of JA Regulation Under Different Light Conditions Is Primarily Determined by SlPIF1a Accumulation and Acetylation

Several studies have demonstrated that JA signaling interacts closely with light signaling pathways [34, 35, 36, 37]. Our research revealed that JA negatively regulates *SlPIF1a*, a critical repressor of light‐mediated carotenoid biosynthesis. RT‐qPCR results showed that JA treatment significantly suppressed *SlPIF1a* expression in tomato fruits, whereas no such inhibition was detected in the *Slmyc2* mutant line (Figure 3B), indicating that SlMYC2 mediates JA‐dependent repression of *SlPIF1a*. Results from Dual‐LUC, Y1H, and EMSA assays confirmed that SlMYC2 binds directly to the *SlPIF1a* promoter and represses its transcription (Figure 3C–G). Therefore, under light conditions, JA activates SlMYC2, which suppresses *SlPIF1a* expression, reducing its interference with SlMYC2‐driven activation of *SlPSY1* and ultimately enhancing lycopene accumulation—consistent with previous findings [24, 28]. In contrast, under dark conditions, JA induces *SlNATA1* expression, resulting in acetylation of the accumulated SlPIF1a protein. The acetylated SlPIF1a then represses *SlPSY1* expression, leading to decreased carotenoid accumulation (Figure 6). The phenotypic observation that lycopene accumulation in *Slpif1a‐3* mutant fruits was strongly inhibited following JA treatment further supports the notion that SlPIF1a accumulation determines the directional effect of JA on lycopene biosynthesis. Recent studies have highlighted that light conditions alter plant responses to hormonal or environmental cues. For example, Liu et al. demonstrated that JA promotes *CYP71AV1* expression and artemisinin synthesis in *A. annua* only under light conditions [35]. Similarly, Zhang et al. reported that under light, the protein kinase PPK1 promotes photomorphogenesis by phosphorylating HY5, whereas in darkness, phosphorylated HY5 interacts with COP1 and BBX24, accelerating its ubiquitination and degradation, thereby inhibiting photomorphogenesis in *Arabidopsis* [66]. These examples emphasize that plants may employ distinct hormonal response mechanisms depending on light exposure, a concept consistent with our observations. However, this study provides no direct evidence regarding whether MeJA affects SlPIF1a protein stability. Furthermore, the potential ubiquity of the observed JA‐PIF1a‐mediated, light‐dependent regulation of lycopene in other tissues or fruit systems requires further investigation.

In recent years, increasing evidence has shown that post‐translational modifications (PTMs)—including phosphorylation, SUMOylation, and glycosylation—play pivotal roles in regulating plant protein function. For instance, SUMOylation of TaHsfA1 in wheat enhances its transcriptional activity and heat tolerance [67], while phosphorylation of TaCBF1 by TaPsIPK1 strengthens stripe‐rust resistance [68] In banana, MaMPK6‐3 phosphorylates the transcription factor MabZIP21 at T318 and S436, promoting fruit ripening [69]. In *Arabidopsis*, COP1 prevents BIN2‐mediated phosphorylation of HSFA1d under high temperatures, influencing its subcellular localization [70]. Comparable regulatory roles of PTMs are also observed in animals. Acetylation of the tumor suppressor p53 enhances its binding affinity to downstream target genes [71], and deacetylation of the transcription elongation factor FolTFIIS facilitates its nuclear translocation by FolIws1, promoting sporulation‐related gene expression and microconidia formation in *Fusarium oxysporum* [72]. Despite these insights, the role of acetylation in modulating plant transcription factors remains underexplored. Our findings demonstrate that the acetyltransferase SlNATA1 enhances the transcriptional repressive activity of SlPIF1a via acetylation—an uncommon modification for a protein typically functioning as a transcriptional activator. We propose that acetylation may alter the spatial conformation of SlPIF1a, exposing its inhibitory domain and converting it into a transcriptional repressor. This observation highlights acetylation as a potentially critical regulatory mechanism in plant PTMs [73]. One limitation of this study is the absence of phenotypic data on lycopene biosynthesis in *Slnata1* mutants. Furthermore, whether acetylation can alter the subcellular localization of SlPIF1a and consequently affect its other biological functions remains to be explored in future work.

## 4 Materials and Methods

### 4.1 Plant Materials and Growth Conditions

The tomato cultivar Ailsa Craig (AC) was used for wild type and the background plant for genetic transformation. The WT and all the transgenic plants were grown in a greenhouse in China Agricultural University (Beijing, 116.15891°E, 40.08948°N). Tomato seeds were germinated in a growth medium composed of a mixture of peat, perlite, and vermiculite (3:1:1, v/v) in trays in a growth chamber. The seedlings, aged 35 days, were transplanted into the soil of the greenhouse.

The tomato fruits used in treatment experiment were collected at 35 DAF (MG stage) treated with a 0.2% ethanol aqueous solution for the control group or 200 µM MeJA for the MeJA group, over a total of 120 h. MeJA was initially dissolved in 100% ethanol at a concentration of 100 mM, then diluted to 200 µM with water. Fruits in the light groups were stored at 24°C under 16 h light (250 µmol m^−2^ s^−1^) and 8 h dark cycle, while those in the dark groups were stored at 24°C under dark condition throughout the process. In each group, 15 fruits were divided into five sets. The pericarps from each set were carefully sampled with surgical blades, thoroughly mixed, and then rapidly frozen in liquid nitrogen before being stored at −80°C for analyses of carotenoids, gene expression, and protein extraction. Each set of pericarps served as one biological replicate.

### 4.2 Quantification of Carotenoids Content

The carotenoids content of the fruits was detected using high‐performance liquid chromatography (LC‐20AT; SHIMADZU, Japan) as described previously [27]. Pericarps (0.6 g) were homogenized with 30 mL of hexane/acetone/ ethanol (1:1:1 by vol.) solution and magnetically stirred for 30 min. The extracts were subjected to centrifugation at 3000 g and 4°C for 10 min, after which 15 mL of water was added. The upper layer was transferred to a round‐bottom flask, and 6 mL of it was evaporated to dryness using a rotary evaporator at 30°C. The remaining residue was dissolved in a solution of THF/acetonitrile/methanol (15:30:55 by volume) to achieve a final volume of 6 mL. The final solution was filtered through 0.45 µm membrane filters, and 20 µl was injected into the HPLC system detection at a wavelength of 450 nm. The standard of lycopene (Sigma‐Aldrich, SMB00706) was detected with a retention time of 34.6–35.2 s; the standard of β‐carotene (ZZSTADARDS, ZT‐30147) was detected with a retention time of 20.8 – 21.2 s; and the standard of lutein (phytolab, PHL89723) was detected with a retention time of 7.8–8.2 s. All procedures were carried out under dim light. Each set of pericarps served as one biological replicate.

### 4.3 Co‐Immunoprecipitation Assay (Co‐IP)

The SlPIF1a‐FLAG and SlMYC2‐N‐YFPC‐myc were transformed into *Agrobacterium tumefaciens* strain GV3101 and co‐infiltrated into *N. benthamiana* leaves. As a negative control, the 35S‐SlMYC2‐myc and 35S‐SlbHLH59‐FLAG vectors were also transformed into *Agrobacterium* and infiltrated into *N. benthamiana* leaves. Approximately 72 h post‐infiltration, total proteins were extracted using a lysis buffer consisting of 220 mM Tris‐HCl (pH 7.4), 250 mM sucrose, 1 mM MgCl_2_, 50 mM KCl, and 10 mM β‐Mercaptoethanol. Immunoprecipitation assays were conducted using about 20 µl of anti‐FLAG affinity beads (SA042001, Smart‐lifesciences) to purify the SlPIF1a and SlbHLH59 protein expressed in *N. benthamiana* leaves. Subsequently, a western blot analysis was performed on the samples, utilizing anti‐FLAG (1:5000, HT201‐01, TransGen Biotech) and anti‐myc antibodies (1:5000, HT101‐01, TransGen Biotech) for detection. The primers used for constructing vectors are listed in Supplemental Table S1.

### 4.4 In Vitro Acetylation Assay

The full‐length CDS of *SlPIF1a* was amplified and inserted into the pCOLD‐TF expression vector, while the full‐length CDS of *SlNATA1* was cloned into the pGEX‐4T‐1 vector. Protein expression was carried out in *Escherichia coli* Rosetta cells, induced with 0.2 mM IPTG, and incubated at 28°C for 16 h. Following incubation, the cells were collected and resuspended in a buffer (50 mM Tris‐HCl, pH = 7.8, 150 mM NaCl, and 1× protease inhibitor). The cells were disrupted using an ultrasonic homogenizer (200 W) for 30 min on ice to release the proteins. Recombinant His‐SlPIF1a and GST‐SlNATA1 proteins were purified using NiNTA affinity chromatography (Cytiva Sweden AB, Uppsala, Sweden, Lot. 17531806) and Glutathione Sepharose beads (Cytiva Sweden AB, Uppsala, Sweden, Lot. 10299550), respectively.

For the in vitro acetylation assay, each reaction mixture included purified recombinant His‐SlPIF1a and 0, 0.5, 1, 2, 4 µg GST‐SlNATA1 protein in a 50 µL assay buffer, which contained 50 mM Tris–HCl (pH 8.0), 1 mM acetyl‐CoA (Roche Diagnostics GmbH, Mannheim, Germany, Cat. No. 53056420), 50 mM NaCl, 5 mM MgCl_2_, 2 mM Nicotinamide (NAM), 1 mM sodium butyrate, and 5% glycerol. The reactions were incubated at 30°C, and then 12.5 µL of 5× SDS loading buffer was added to the samples, which were boiled for 5 min to terminate the enzyme activity. A western blot analysis was subsequently conducted on the samples [74]. The anti‐acetyllysine antibody (PTM BioLab, Inc., Hangzhou, China, Lot. PTM‐101, 1:1000) was used to determine the level of protein acetylation. The primers used for constructing vectors are listed in Supplemental Table S1.

### 4.5 In Vivo Acetylation Assay

The CDS of *SlNATA1* was cloned into the pCAMBIA1300‐35S‐N‐YFPN vector, creating SlNATA1‐N‐YFPN‐myc construct. This vector and SlPIF1a‐FLAG were then introduced into the *Agrobacterium tumefaciens* strain GV3101 and co‐infiltrated into the leaves of *N. benthamiana*. As a negative control, the 35S‐SlPIF1a‐FLAG vector alone was transformed into *Agrobacterium* and infiltrated into *N. benthamiana* leaves. Approximately 72 h post‐infiltration, total proteins were extracted using a lysis buffer consisting of 220 mM Tris‐HCl (pH 7.4), 250 mM sucrose, 1 mM MgCl_2_, 50 mM KCl, and 10 mM β‐Mercaptoethanol. Immunoprecipitation assays were conducted using about 20 µl of anti‐FLAG affinity beads (SA042001, Smart‐lifesciences) to purify the SlPIF1a‐FLAG protein expressed in *N. benthamiana* leaves. Subsequently, western blot analyses were performed on the samples, utilizing anti‐FLAG (1:5000, HT201‐01, TransGen Biotech) and anti‐ acetylcysteine antibodies (PTM BioLab, Inc., Hangzhou, China, Lot. PTM‐101, 1:1000) for detection. The primers used for construct vectors are listed in Supplemental Table S1.

### 4.6 Statistical Analysis

The data from RT‐qPCR and Dual‐Luc assays were normalized. The sample size (n) for each statistical analysis is indicated in the respective figure legends. Mean values and standard errors and statistical analyses were computed by using Graphpad Prism 8. Results are presented as mean ± SE. One‐way ANOVA and two‐way ANOVA were employed for multiple comparisons, and post hoc tests were conducted using Tukey's multiple comparisons test, Šídák's multiple comparisons test, and Fisher's LSD test. The specific test methods are specified in the corresponding figure legends. Two‐tailed Student's *t*‐test was utilized to assess statistical significance between two groups. A summary of these analyses can be found in the Supplemental Data Set 1.

### 4.7 Accession Numbers

Sequence data from this article can be found in the SGN (Sol Genomics Network) data under the following accession numbers: SlMYC2 (Solyc08g076930); SlPIF1a (Solyc09g063010); SlPSY1 (Solyc03g031860); SlNATA1 (Solyc08g068780); SlJAZ6,(Solyc01g005440); SlDXS (Solyc01g067890); SlGGPS (Solyc02g085700); SlPDS (Solyc03g123760); SlUBQ (Solyc01g056940); SlActin (Solyc11g005330); SlSTOP1 (Solyc11g017140); CsHBP (CsaV3_3G001310); SlDXR (Solyc03g114340); SlMCT (Solyc01g102820); SlCMK (Solyc01g009010); SlMDS (Solyc08g081570); SlZDS (Solyc01g097810); SlZISO (Solyc12g098710); SlCRTISO (Solyc10g081650); SlLCYB (Solyc04g040190); SlOR (Solyc03g093830); SlST2a (Solyc05g011890).

## Author Contributions

**Na Zhang**: conceived this project and designed the research. **Jiayi Xu**: performed most of the experiments. **Lingfeng He**: and **Jia‐long Zhang**: contributed to phenotypic analysis and protein interaction experiments. **Hao Cui** and **Hongxin Li**: contributed to sample preparation. **Haijun Zhang**: contributed to measurement of carotenoid content. **Jiao‐jiao Zhang**: and **Lun Liu**: contributed to the preparation of mutant lines. **Kunpeng Xu**: contributed to the illustration of the mechanism diagram. **Jiayi Xu**: **Yang‐Dong Guo**: and **Na Zhang**: analyzed the data. **Jiayi Xu**: and **Na Zhang**: wrote the article. All authors discussed the manuscript.

## Funding

National Natural Science Foundation of China (32573021), Beijing Rural Revitalization Agricultural Science and Technology Project (NY2401080000) and Beijing Agriculture Innovation Consortium (BAIC01‐2026)

## Conflicts of Interest

The authors declare no competing financial interests.

## Supporting information




**Supporting File**: advs74648‐sup‐0001‐SuppMat.pdf

## Data Availability

The data that support the findings of this study are available in the supplementary material of this article.
