# Supplementary material for: Divergent Regulatory Effects of Jasmonic Acid on Tomato Lycopene Biosynthesis Under Light and Dark Conditions
Source: Adv Sci (Weinh). 2026 Mar 2;13(26):e13249. doi: 10.1002/advs.202513249 (PMC13159117; doi:10.1002/advs.202513249)
Supplement: Supplementary file 1 — Supporting File: advs74648‐sup‐0001‐SuppMat.pdf [file ADVS-13-e13249-s001.pdf]

Supporting Information for

Divergent Regulatory Effects of Jasmonic Acid on Tomato Lycopene Biosynthesis under Light and Dark Conditions

Jiayi Xu[1,3], Lingfeng He[1], Jialong Zhang[1], Hao Cui[1], Hongxin Li[1], Haijun Zhang[2], Jiaojiao Zhang[4], Lun Liu[5], Kunpeng Xu[6], Yang-Dong Guo[1], Na Zhang[1*]

1. College of Horticulture, China Agricultural University, Beijing, 100193, China.
2. Supervision, Inspection and Test Center of Vegetable Seed Quality of Ministry of Agriculture and Rural Affairs, State Key Laboratory of Vegetable Biobreeding, Beijing Vegetable Research Center (BVRC), Beijing Academy of Agriculture and Forestry Science (BAAFS), Beijing 100097, China
3. College of Food Science and Engineering, Ningbo University, Ningbo, 315211, China
4. Hebei Key Laboratory of Horticultural Germplasm Excavation and Innovative Utilization, College of Horticulture Science and Technology, Hebei Normal University of Science and Technology, Qinhuangdao 066004, China
5. College of Horticulture, Anhui Agricultural University, Hefei, 230036, China
6. College of Agriculture, Fujian Agriculture and Forestry University, Fuzhou, 350002, China

* Author for correspondence: Na Zhang
Email: (zhangna@cau.edu.cn)

Supplemental Materials and Methods

Generation of transgenic lines

To generate *SlPIF1a*-overexpressing plants, the 1674 bp full-length CDS regions of *SlPIF1a* was PCR-amplified from the cDNA of tomato. The PCR product was inserted into the vector pCAMBIA1305-35S-FLAG to generate SlPIF1a-FLAG (*1*). The specific primers were designed to identify the transgenic plants overexpressing *SlPIF1a*. The target sites used for the CRISPR/Cas-9-mediated genome editing of *SlPIF1a* were selected by CCTop (https://crispr.cos.uni-heidelberg.de/) and the single guide RNA (sgRNA) containing the selected target sites was cloned into the binary vector pBSE402 (*2*). These recombinant vectors were introduced into *Agrobacterium tumefaciens* GV3101 and transferred into tomato cotyledons by transformation and regeneration (*3*). Deletions and insertions in *SlPIF1a* were identified by PCR and DNA sequencing. Four potential off-target sites were predicted using the CRISPR-P website (http://crispr.hzau.edu.cn/CRISPR2/). PCR and DNA sequencing experiments showed that the *SlPIF1a* strains did not exhibit off-target editing. The primers used were detailed in Supplemental table 1.

Total RNA isolation and reverse transcription quantitative PCR (RT-qPCR)

The total RNA was extracted from the pericarp of tomato fruits harvested 35 DAFB (MG stage) using an RNA extraction kit (TIANGEN, Shanghai, China). Total RNA (1 μg) was reverse transcribed using the qPCR RT Kit (AidLab Biotech, China). RT-qPCR analyses were performed on a QuantStudio 6 Real-Time PCR Detection System (Applied Biosystems, USA). The relative expression levels were normalized to the expression level of the tomato housekeeping gene *SlActin2* and *SlUBQ*. Each experiment was repeated with three independent samples. The primers used for RT-qPCR are listed in Supplemental Table 1.

Luciferase complementation Imaging (LCI)

The full-length, N-terminal (1-882 bp) and C-terminal (883-2070 bp) CDS regions of *SlMYC2*, *SlNATA1* was cloned and inserted into the vector pCAMBIA1300-nLUC. The full-length, N-terminal (1-750 bp), M-terminal (181-750 bp) and C-terminal (751-1674) CDS region of *SlPIF1a* was cloned and inserted into the vector pCAMBIA1300-cLUC. The resulting vectors were transformed into *Agrobacterium tumefaciens* strain GV3101. SlMYC2-nLUC, cLUC-SlPIF1a were co-infiltrated into *N. benthamiana* leaves and SlNATA1-nLUC, cLUC-SlPIF1a, cLUC-SlPIF1aN and cLUC-SlPIF1aM were co-infiltrated into *N. benthamiana* leaves respectively. The SlMYC2-nLUC and cLUC-SlJAZ6 were used as positive control. The protein CsHBP and one of the target proteins were co-expressed as negative control. Approximately 72 h after infiltration, the luciferin was sprayed on leaves within the infiltration region for 3 minutes and fluorescence was detected using the Luc 2019 imaging apparatus. The primers used for construct vectors are listed in Supplemental Table 1.

Bimolecular fluorescence complementation assays (BiFC)

The CDS regions of *SlPIF1a*, *SlBHLH59* and *SlMYC2* were inserted into the pCAMBIA1300-35S-N-YFPN, pCAMBIA1300-35S-N-YFPN and pCAMBIA1300-35S-N-YFPC vectors to generate SlPIF1a-N-

YFPN-HA, SlBHLH59-N-YFPN-HA and SlMYC2-N-YFPC-myc respectively. The resultant vectors were transformed into *Agrobacterium tumefaciens* strain GV3101 and co-infiltrated into *N. benthamiana* leaves. Approximately 72 h after infiltration, cell nuclei were stained with DAPI (Sigma-Aldrich, D9542) for 30 minutes. Fluorescence signal of YFP was analyzed at wavelength 514 nm for excitation and fluorescence signal of DAPI was analyzed at wavelength 460 nm for excitation using a confocal microscope (Leica SP8). The primers used for construct vectors are listed in Supplemental Table 1. A total of approximately 40 cells from sections taken from three different tobacco plants were observed.

Subcellular localization analysis

The CDS region of *SlPIF1a* was inserted into the pCAMBIA1300-super-N-GFP vector, The resultant vector was transformed into *Agrobacterium tumefaciens* strain GV3101 and infiltrated into *N. benthamiana* leaves. Approximately 72 h after infiltration, fluorescence signal of GFP was analyzed using a confocal microscope (Leica SP8). Cell nuclei were stained with DAPI (Sigma-Aldrich, D9542). Wavelength for GFP excitation was 488 nm and wavelength for DAPI excitation was 460 nm. The primers used for construct vectors are listed in Supplemental Table 1. A total of approximately 40 cells from sections taken from three different tobacco plants were observed.

Yeast two-hybrid assays (Y2H)

The full-length, N-terminal (1-882 bp), C-terminal (883-2070 bp) CDS regions of *SlMYC2* and the full-length, N-terminal (1-750 bp), C-terminal (751-1674) CDS regions of *SlPIF1a* were cloned and inserted into the vector pGBKT7. The full-length, C-terminal (883-2070 bp) CDS regions of *SlMYC2* and the full-length, C-terminal (751-1674) CDS of *SlPIF1a* were cloned and inserted into the vector pGADT7. Y2H assays were performed using the Matchmaker GAL4 Two-Hybrid System (Clontech, America). Vectors used to test interactions were transformed into yeast (*Saccharomyces cerevisiae*) strains Y2HGold. The empty pGADT7 vector and SlPIF1aC-BD were transformed in parallel as a negative control. Co-transformation of SlMYC2-AD and SlJAZ6-BD served as a positive control (*4*). The transformed yeast cells were plated on QDO medium containing 40 mg/L X-α-GAL for stringent detection of protein–protein interactions. The primers used for construct vectors are listed in Supplemental Table 1.

Yeast one-hybrid assays (Y1H)

The promoter sequence of *SlPIF1a* (*P2*) containing G-box and *SlPSY1* promoter containing both PBE-box and G-box were cloned into pLACZi. The *SlNATA1* was inserted into the pGADT7 vector. Experimental vectors were co-introduced into the yeast strains YM4271. The empty pGADT7 vector and experimental pLACZi vector were co-transformed in parallel as a negative control. The transformed yeast cells were plated on SD/-UL medium for 72 hours and transferred onto aseptic filter paper. Then the filter paper was put into liquid nitrogen to crack the yeast strains. Wet the filter paper using the cracking solution (8.517 g/L Na_2HPO_4, 4.7984 g/L NaH_2PO_4, 0.75 g/L KCl, 0.246 g/L $MgSO_4 \cdot 7H_2O$) with 300 mg/L X-Gal and stored at 30 ℃ for 0.5 – 6 hours. Blue plaques indicate interaction between DNA and protein. The primers used for construct vectors are listed in Supplemental Table 1.

Dual-luciferase reporter assay (Dual-LUC)

The *SlPIF1a* promoter fragments (*P1* and *P2*) containing G-box and *SlPSY1* promoter fragment containing PBE-box and G-box were cloned into pGreenII 0800-miniLUC vector as reporter constructs. The CDS of *SlMYC2*, full length, N-terminal, C-terminal of *SlPIF1a* and *SlNATA1* were inserted into the pGreenII 62-SK vector to generate effector constructs. Empty pGreenII 62-SK vector together with reporter constructs were used as negative controls. The resultant constructs were transformed into *Agrobacterium tumefaciens* strains GV3101 (p19) and transiently expressed in *N. benthamiana* leaves. 72 h after infiltration, the activity levels of LUC and Ren signals were determined using Dual Luciferase Reporter Assay Kit (DL101-01, Vazyme Biotech Co., Ltd.). The primers used for construct vectors are listed in Supplemental Table 1.

Electrophoretic mobility shift assay (EMSA)

EMSA was conducted following the protocol outlined by Liu et al. with modification (*4*). The full-length CDS of *SlMYC2* was amplified using gene-specific primers and cloned into the expression vector pMAL-p5x. The construct was then introduced into *Escherichia coli* BL21 (DE3). The transformed cells were cultured at 37°C, and the expression of the transgenes was initiated by adding IPTG to a final concentration of 2 mM when the optical density at 600 nm (OD_{600}) reached 0.6. The cultures were subsequently incubated at 28°C for 14 hours. The SlMYC2-MBP protein was purified using MBP-tag purification resin (E8021V, New England Biolabs) according to the manufacturer's guidelines.

Oligonucleotides (Supplemental Table 2) contained G-box were synthesized and biotinylated at the 3’ end. Standard reaction mixtures for EMSA were prepared in the following sequence: 2 µl of 10× binding buffer, 1 µl of 50% glycerol, 1 µl of 100 mM $MgCl_2$, 1 µl of 1 µg/µL Poly (dI•dC), 1 µl of 1% NP-40, 2 µg of protein, 2 µl of biotin-labeled probe (20 ng/µl), the necessary volume of unlabeled probe, and ddH_2O to reach a final volume of 20 µl. The reaction mixtures were incubated at 25°C for 60 minutes and then subjected to electrophoresis on 8% native polyacrylamide gels. Following separation, the components were transferred to an Amersham Hybond™ N^+ nylon membrane (GE Healthcare) in 0.5X TBE buffer at 380 mA at 4°C for 1 hour. After UV cross-linking, biotin-labeled DNA was detected using a LightShift Chemiluminescent EMSA kit (Thermo Scientific, Waltham, MA, USA). The primers used for construct vectors are listed in Supplemental Table 1.

Production of the SlPIF1a antibody

The polyclonal antibody raised against SlPIF1a was designed and generated by Genscript (Nanjing, China). The antibody was produced by immunizing rabbits with a synthetic RKRKGREMEDEGQN (1th – 14th aa) peptide. An extra "C" was added to the N terminus to facilitate conjugation.

Supplemental Figures

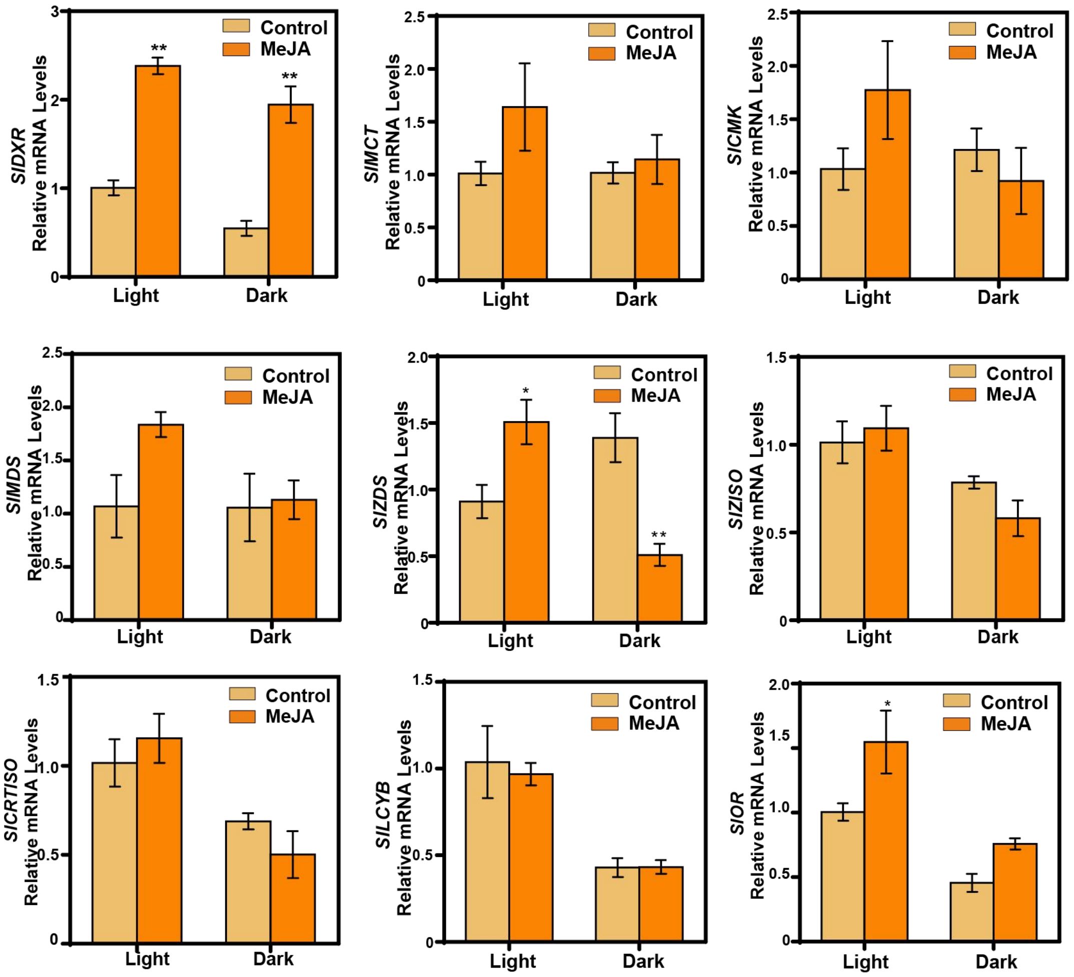

Fig. S1.

Jasmonic acid (JA) influences the expression levels of genes involved in carotenoid synthesis and regulation under light and dark conditions. WT fruits were harvested at mature green stage (MG) and experimental treatment was consistent with Figure 1. The mRNA levels of carotenoid synthesis and regulation genes were determined. Tomato housekeeping gene *SlUBQ* (Solyc01g056940) and *SlActin2* (Solyc11g005330) were used as internal control. Each sample contained pericarps from three different fruits as a biological replicate. Three replicates were performed per experiment. Values represent means ± SE. Asterisks indicate statistically significant differences analyzed by two-way ANOVA (* $P \leq 0.05$, ** $P \leq 0.01$, Šídák's multiple comparisons test). Source data and statistical summary can be found in the Supplement Data Set 1.

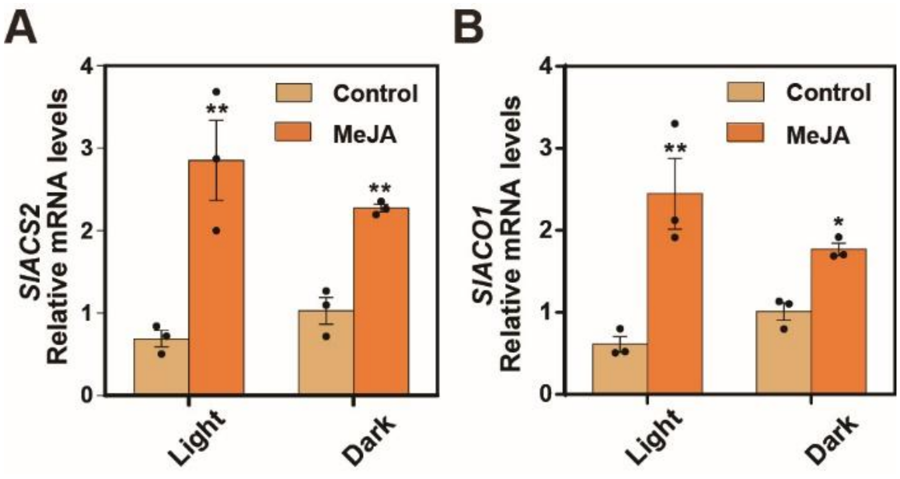

Fig. S2.

Jasmonic acid (JA) bidirectionally regulates carotenoid synthesis under different light conditions through an ethylene-independent pathway. WT fruits were harvested at mature green stage (MG) and experimental treatment was consistent with Figure 1. (A) The mRNA levels of *SlACS2* and (B) *SlACO1* were determined. Tomato housekeeping gene *SlUBQ* (Solyc01g056940) and *SlActin2* (Solyc11g005330) were used as internal control. Each sample contained pericarps from three different fruits as a biological replicate. Three replicates were performed per experiment. Values represent means ± SE. Asterisks indicate statistically significant differences analyzed by two-way ANOVA (* $P \leq 0.05$, ** $P \leq 0.01$, Uncorrected Fisher's LSD). Source data and statistical summary can be found in the Supplement Data Set 1.

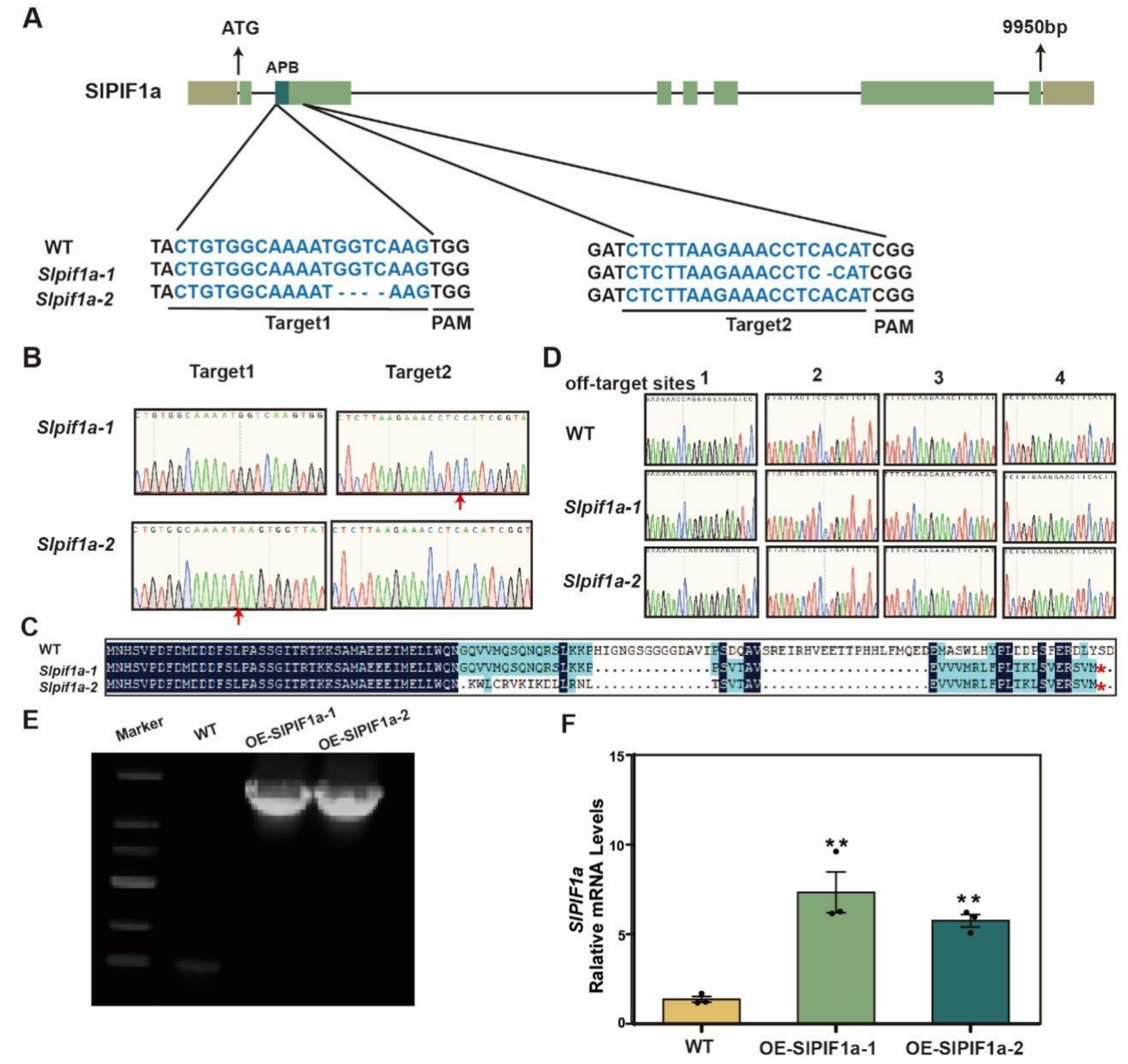

Fig. S3.

The characterization of *SlPIF1a* transgenic lines. (A) Brown squares represent the UTR region, aqua squares represent exons, and black solid lines represent introns. The bottle green square upon the second exon is the sequence encoding APB domain. The two sgRNA target sites of *SlPIF1a* with blue text are presented by Schematic illustration. The PAM motifs (NGG) are indicated by black underline. The red letters represent inserted bases, and the blue horizontal lines represent deleted bases. (B) The nucleotide sequencing results of the *SlPIF1a* homozygous mutant lines. The red arrows indicate editing sites. (C) The amino acid sequence of the *SlPIF1a* homozygous mutant lines. The results indicate that SlPIF1a protein translation in mutant lines of *Slpif1a-1*, *Slpif1a-2* are early terminated. (D) Detection of potential off-target sites of *Slpif1a-1*, *Slpif1a-2.* (E) The overexpression lines of *SlPIF1a* were characterized by PCR. (F) SlPIF1a transcript levels were determined in two overexpression lines (OE-SlPIF1a-1 and OE-SlPIF1a -2) by RT-qPCR. Tomato housekeeping gene *SlUBQ* (Solyc01g056940) and *SlActin2* (Solyc11g005330) were used as internal control. Data are presented as mean ± SE of three biological replicates. Unpaired student's *t*-test, two-tailed (***P* ≤ 0.01). Source data and statistical summary can be found in the Supplement Data Set 1.

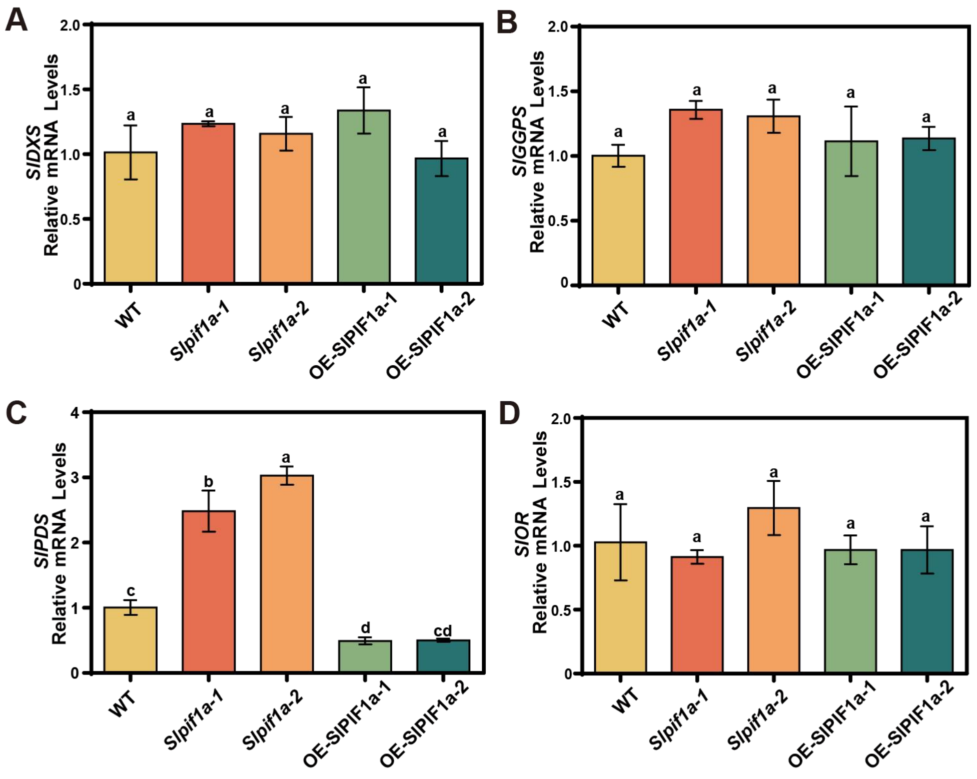

Fig. S4.

SlPIF1a's influence in genes involved in carotenoid synthesis and regulation. The mRNA levels of carotenoid synthesis and regulation genes were determined with a method consistent with Figure 1E. Samples were collected using the same method as in Figure 2C. Each sample contained pericarps from three different fruits as a biological replicate. Three replicates were performed per experiment. Values represent means ± SE. Different lowercase letters were used to indicate statistically significance difference ($P \leq 0.05$) by one-way ANOVA, Tukey's multiple comparisons test. Source data and statistical summary can be found in the Supplement Data Set 1.

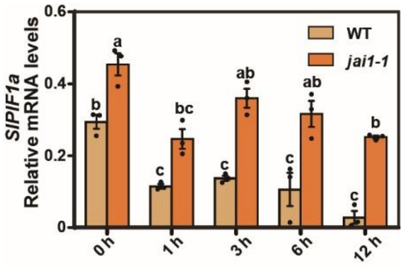

Fig. S5.

Analysis of *SlPIF1a* expression treated by MeJA. The mRNA level of *SlPIF1a* was determined using RT-qPCR in leaves of WT and *jai1-1* mutant lines treated by 200 μM MeJA. Data are presented as means ± SE of three biological replicates. Two -way ANOVA, $P \leq 0.05$, Tukey's multiple comparisons test. The *P*-value is detailed in Supplementary table 3.

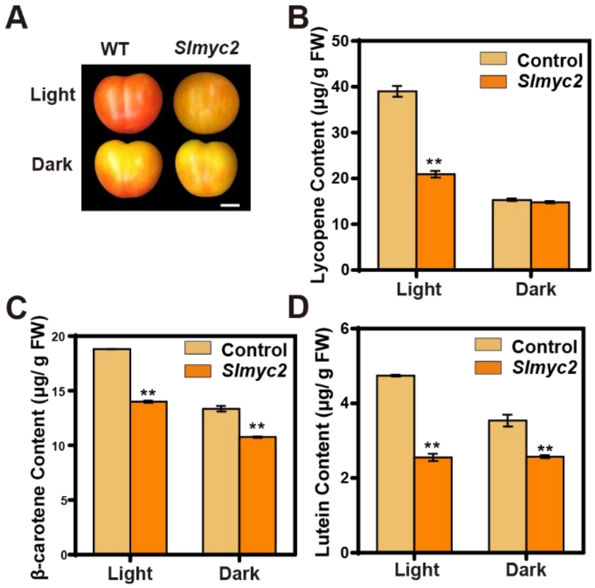

Fig. S6.

The difference of carotenoid content in WT and *Slmyc2* fruits under light and dark conditions. (A) The fruits of WT and *Slmyc2* were harvested at MG stage and stored for 5 days. Light treatment was consistent with Figure 1A. Scale bar = 1 cm. Detailed analysis of lycopene (B), β-carotene (C) and lutein (D) concentrations in fruit pericarps under light and dark conditions in WT and *Smyc2* were measured. Each sample contained pericarps from three different fruits as a biological replicate. Three replicates were performed per experiment. Asterisks indicate statistically significant differences analyzed by two-way ANOVA (* $P \leq 0.05$, Šídák's multiple comparisons test). Source data and statistical summary can be found in the Supplement Data Set 1.

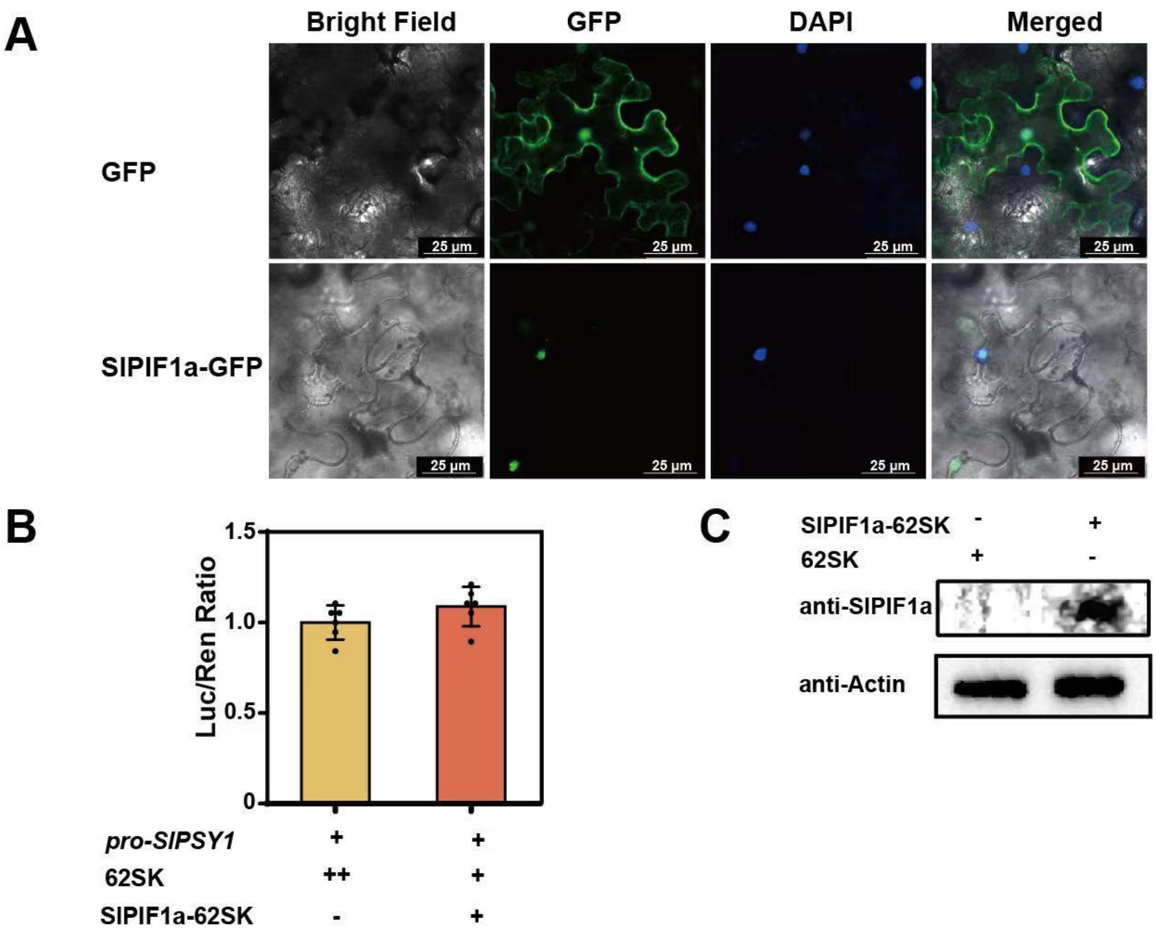

Fig. S7.

SlPIF1a could not repress *SlPSY1* expression alone. (A) The CDS of *SlPIF1a* was ligated downstream of the coding sequence of a GFP tag and transiently expressed using the super promoter in pSuper1300 in *N. benthamiana* leaves (GFP-SlPIF1a). DAPI was used for Cell nuclei staining overexpression of GFP alone (GFP) was used as a control. Scale bar = 25 µm. (B) Dual-LUC assay showing the effect of SlPIF1a on the promoter activity of *SlPSY1*. The promoter activity of *SlPSY1* was indicated by the activity ratio of LUC /REN. '+' and '−' respectively represent the presence or absence of corresponding plasmids in experimental *N. benthamiana* leaves. Data are presented as means ± SE, n=6. Unpaired student's *t*-test, two-tailed. Source data and statistical summary can be found in the Supplement Data Set 1. (C) Expression of *SlPIF1a* in *N.benthamiana* leaves was detected by Western blot.

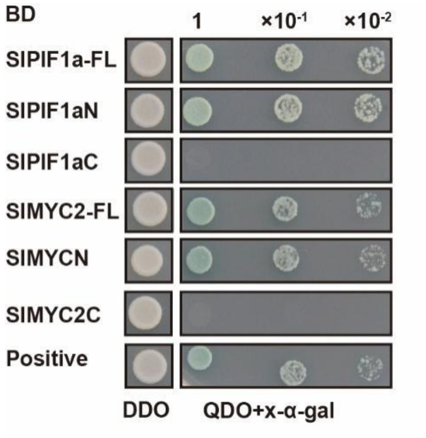

Fig. S8.

The N-terminal of SlPIF1a and SlMYC2 showed transcriptional activation function. The transcription activation functional analyses were detected in Y2H gold yeast strain. DDO, SD medium lacking Trp and Leu; QDO, SD medium lacking Trp, Leu, His, and Ade; X-α-gal, 5-Bromo-4-chloro-3-indoxyl-α-D-galactopyranoside. Blue plaques indicate the protein have transcriptional activation function. SlSTOP1-BD was used as positive control.

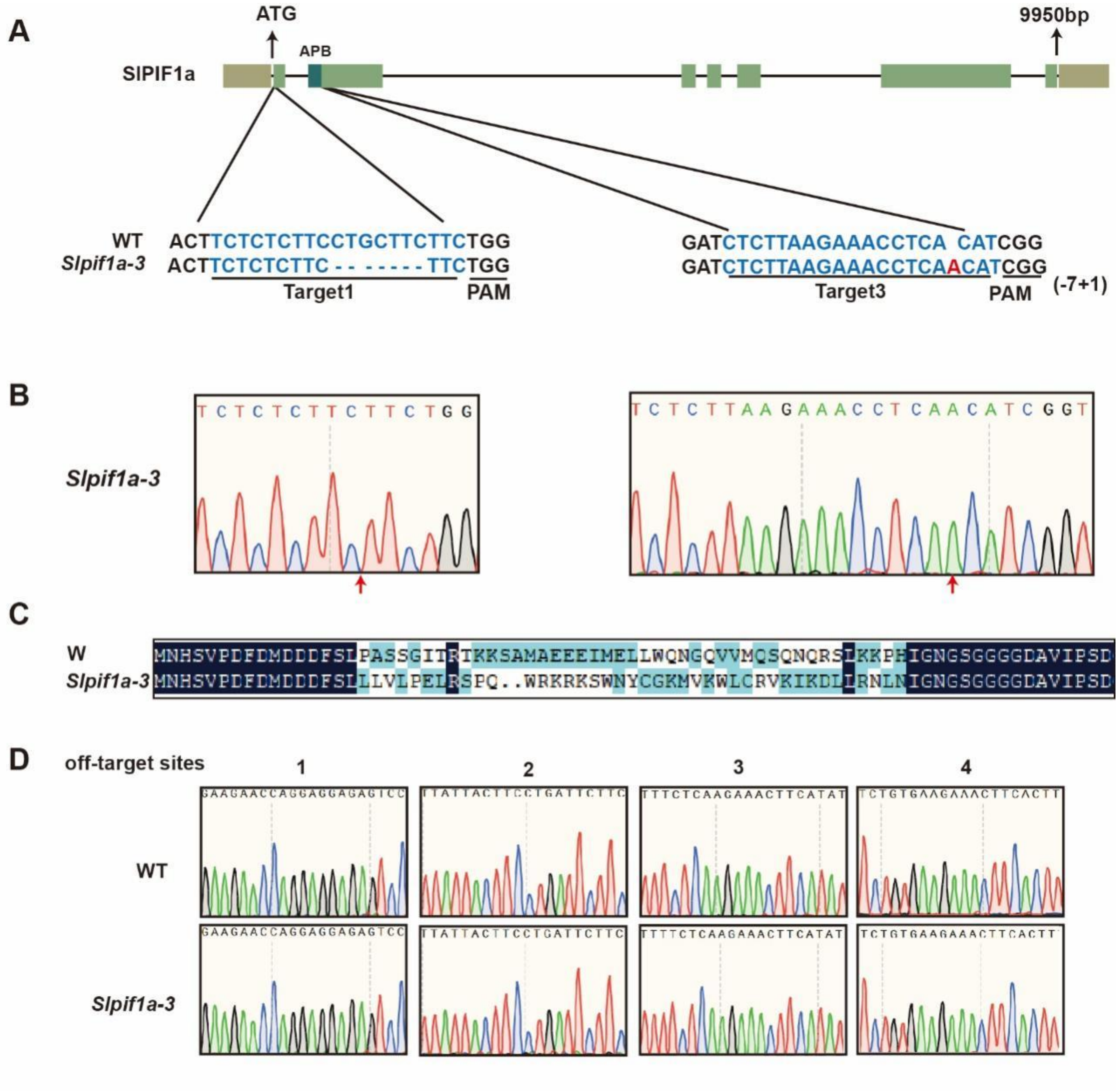

Fig. S9.

The characterization of *Slpif1a-3* line. (A) Brown squares represent the UTR region, aqua squares represent exons, and black solid lines represent introns. The bottle green square upon the second exon is the APB domain. The two sgRNA target sites of SlPIF1a with blue text are presented by Schematic illustration. The PAM motifs (NGG) are indicated by black underline. The red letters represent inserted bases, and the blue horizontal lines represent deleted bases. (B) The nucleotide sequencing results of the *SlPIF1a* homozygous mutant lines. The red arrows indicate editing sites. (C) The amino acid sequence of the *SlPIF1a* homozygous mutant lines. The results indicate that N terminal of SlPIF1a protein in *Slpif1a-3* is mutated with APB domain destroyed. (D) Detection of potential off-target sites of *Slpif1a-3*.

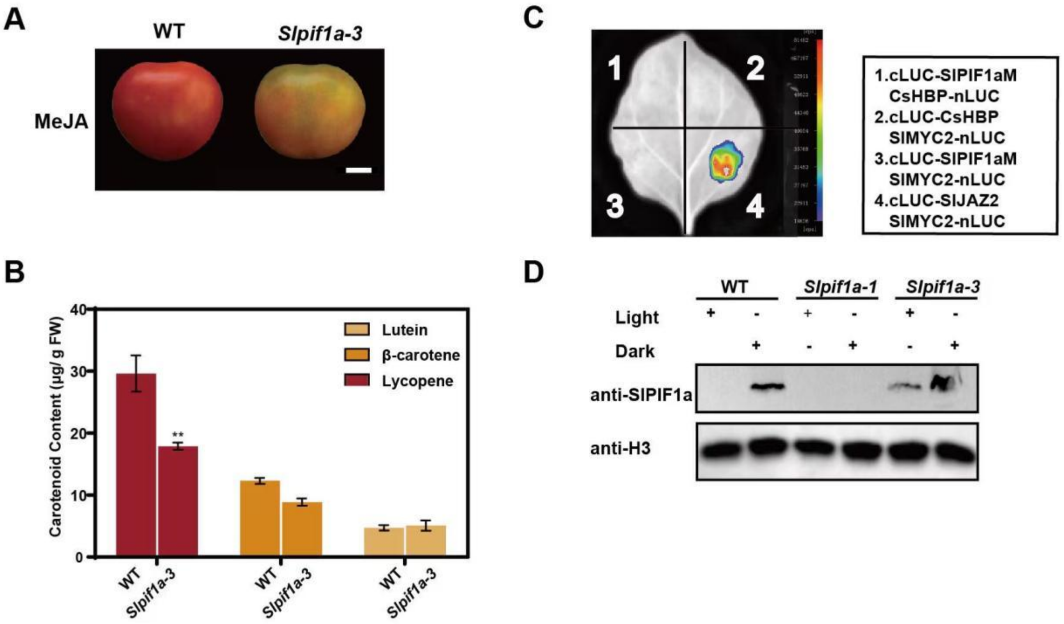

Fig. S10.

JA repressed carotenoid biosynthesis in the fruits of *Slpif1a-3* line under light conditions. (A) The fruits of WT and *Slpif1a-3* were harvested at MG stage and treated with MeJA for 5 days stored at 16 h light at 24 ℃ and 8 h dark at 18 ℃. Scale bar = 1 cm. (B) Carotenoid concentrations in fruit pericarps of WT and *Slpif1a-3* were measured. Each sample contained pericarps from three different fruits as a biological replicate. Three replicates were performed per experiment. Values represent means ± SE. Asterisks indicate statistically significant differences analyzed by two-way ANOVA (** $P \leq 0.01$, Šídák's multiple comparisons test). Source data and statistical summary can be found in the Supplement Data Set 1. (C) LCI assay showing the interaction fragments between SlPIF1aM and SlMYC2. SlPIF1aM represents the residual N-terminal region of SlPIF1a proteins in *Slpif1a-3*. Experimental group is Group 3 with Group 1,2 acting as negative controls and Group 4 acting as positive control. (D) The accumulation levels of SlPIF1a in fruit pericarps of WT, *Slpif1a-1* and *Slpif1a-3* lines under light and dark conditions. Treatment conditions for light and dark groups were the same as in Figure 2A.

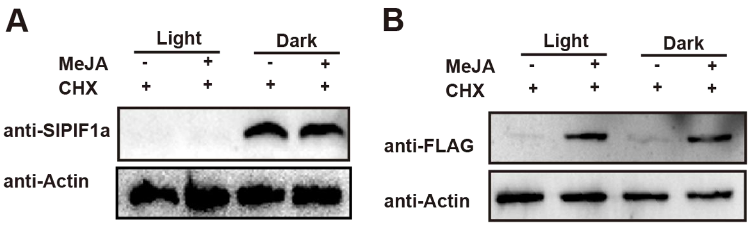

Fig. S11.

Influence of MeJA on SlPIF1a and SlNATA1 protein accumulation levels under light and dark . (A) The accumulation levels of SlPIF1a in WT fruit pericarps with and without MeJA treatment under light and dark conditions. (B) The accumulation levels of SlNATA1 in fruit pericarps of OE-NATA1-1 lines with and without MeJA treatment under light and dark conditions. CHX: Cycloheximide.

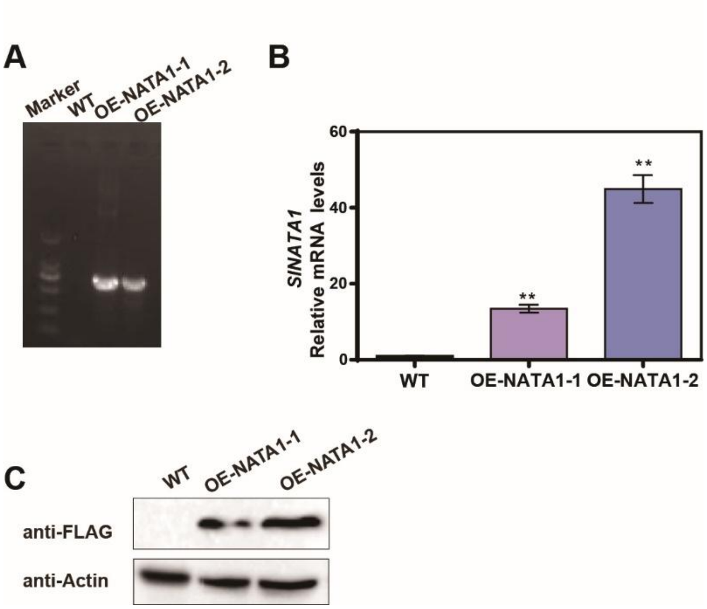

Fig. S12.

The characterization of *SlNATA1* overexpression lines. (A) The overexpression lines of *SlNATA1* were characterized by PCR. (B) SlNATA1 transcript levels were determined in two overexpression lines (OE-NATA1-1 and OE-NATA1 -2) by RT-qPCR. Tomato housekeeping gene *SlUBQ* (Solyc01g056940) and *SlActin2* (Solyc11g005330) were used as internal control. Data are presented as mean ± SE of three biological replicates. Unpaired student's *t*-test, two-tailed (***P* ≤ 0.01). (C) The protein accumulation of SlNATA1-Flag in the leaves of WT and *SlNATA1* overexpression lines.

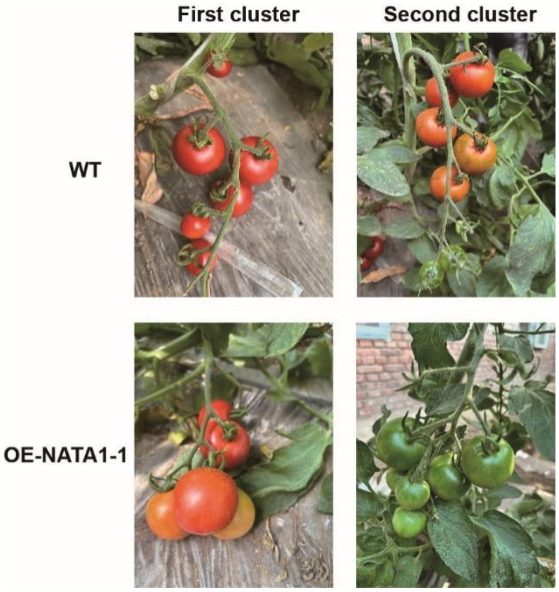

Fig. S13.

Fruit ripening progress of WT and *SlNATA1* overexpression line three months post-transplantation. Fruit ripening patterns of WT and *SlNATA1* overexpression line in first and second fruit clusters three months post-transplantation.

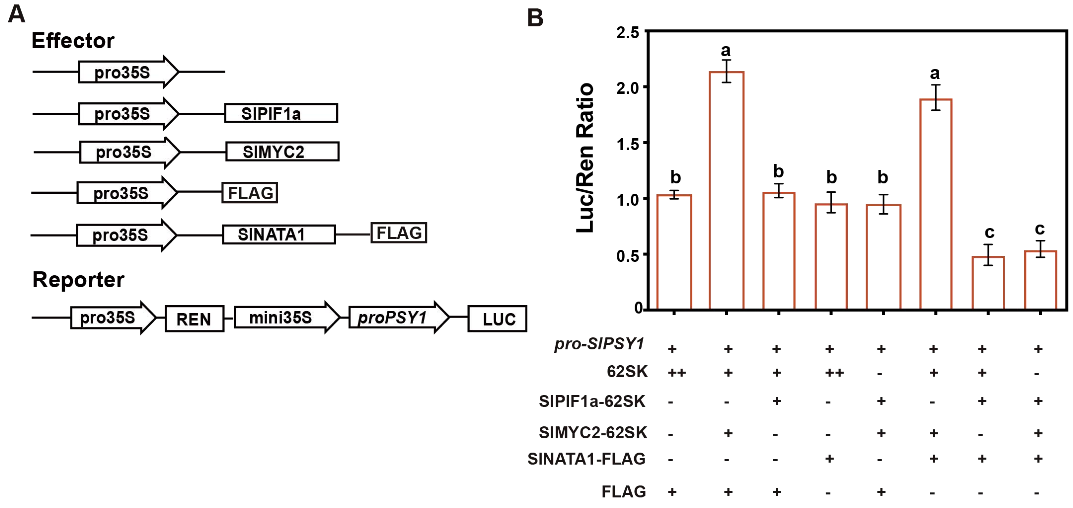

Fig. S14

Hierarchical regulation of the *SlPSY1* promoter by SlPIF1a, SlNATA1, and SlMYC2. The promoter activity of *SlPSY1* was indicated by the activity ratio of LUC /REN. '+' and '−' respectively represent the presence or absence of corresponding plasmids in experimental *N. benthamiana* leaves. Data are presented as means ± SE, n=5. Different lowercase letters were used to indicate statistically significance difference ($P \leq 0.05$) by one-way ANOVA, Tukey's multiple comparisons test. Source data and statistical summary can be found in the Supplement Data Set 1.

Supplementary Table 1. Primers used for PCR amplification.

Primer name	Primer sequence (5'→3')
CR-SlPIF1a	DT1-BsF: ATATATGGTCTCGATTGTCTCTCTTCCTGCTTCTTCGTT<br>DT1-F0: TGTCTCTCTTCCTGCTTCTTCGTTTTAGAGCTAGAAATAGC<br>DT2-R0: AACATGTGAGGTTTCTTAAGAGCAATCTCTTAGTCGACTCTAC<br>DT2-BsR: ATTATTGGTCTCGAAACATGTGAGGTTTCTTAAGAGCAA<br>DT3-BsF: ATATATGGTCTCGATTGCTGTGGCAAAATGGTCAAGGTT<br>DT3-F0: TGCTGTGGCAAAATGGTCAAGGTTTTAGAGCTAGAAATAGC
Off-target1-SlPIF1a	F: GGTCTACGAGACTGTTGAGAC<br>R: CTGGAATATTCATCGGGTCTCT
Off-target-2-SlPIF1a	F: ACAAAGGTTGCAAAATGATGTG<br>R: CCATACAACTCCAGAAGTTCAC
Off-target-3-SlPIF1a	F: TGGTCTCCGTCTTGGATAGTT<br>R: GAGGACTGGGACTATGCC
Off-target-4-SlPIF1a	F: GCGGTTCCTTCGGATTAAG<br>R: CCAACAACCATGGCAACAA
pCAMBIA-1305-SlPIF1a	F: acggaattcgagctcggtaccATGAATCATTCTGTTCCTGATTTTGA<br>R: caggtcgactctagaggatccACCAGATTGATGATTGCCTGG
cLUC-SlPIF1a	F: ccggggcggtacccgggatccaATGAATCATTCTGTTCCTGATTTTGA<br>R: acgaaagctctgcaggtcgacACCAGATTGATGATTGCCTGG
cLUC-SlPIF1aN	F: ccggggcggtacccgggatccaATGAATCATTCTGTTCCTGATTTTGA<br>R: acgaaagctctgcaggtcgacGGTGCTGCCGTTTACTGTACTA
cLUC-SlPIF1aC	F: ccggggcggtacccgggatccaGGAACTGCGACAGCGTCAA<br>R: acgaaagctctgcaggtcgacACCAGATTGATGATTGCCTGG
N-YFPN-SlPIF1a	F: gtctatatcatggccggtaccATGAATCATTCTGTTCCTGATTTTGA<br>R: tactcgaacctgcaggtcgacTTAACCAGATTGATGATTGCCTGG
miniLUC-P1-SlPIF1a	F: ttcctgcagcccgggggatccGTAATTACTTTAAATAGACGGACTCCAT<br>R: ttgtctagaactagtggatccCTTTTACTAACATGTTGATATGCGG
miniLUC-P2-SlPIF1a	F: ttcctgcagcccgggggatccCAAACCCTAGCTATTCAATTTAATTTAGG<br>R: ttgtctagaactagtggatccTGACGTAGTGATTGACCTCCTTC
pLACZi-proPIF1a	F: atgatgaattgaaaagcttCTATAAAAGTCAAACACATCAAAAATTG<br>R: gacagatccccgggtaccGAGAAAATGTGTAACACATACGTAATTA
62SK-SlPIF1a	F: cgctctagaactagtggatccATGAATCATTCTGTTCCTGATTTTGA<br>R: gataagcttgatatcgaattcTTAACCAGATTGATGATTGCCTGG
BD-SlPIF1a	F: tcagaggaggacctgcatatgATGAATCATTCTGTTCCTGATTTTGA<br>R: cgacggatccccgggaattcTTAACCAGATTGATGATTGCCTGG
BD-SlPIF1aN	F: tcagaggaggacctgcatatgATGAATCATTCTGTTCCTGATTTTGA<br>R: cgacggatccccgggaattcGGTGCTGCCGTTTACTGTACTA
BD-SlPIF1aC	F: tcagaggaggacctgcatatgATGGGAACTGCGACAGCGTCAA<br>R: cgacggatccccgggaattcACCAGATTGATGATTGCCTGG
AD-SlPIF1a	F: gtaccagattacgctcatatgATGAATCATTCTGTTCCTGATTTTGA<br>R: atgcccacccgggtggaattcTTAACCAGATTGATGATTGCCTGG
pet28-SlPIF1a	F: atgggtcgcggatccgaattcATGAATCATTCTGTTCCTGATTTTGA<br>R: tgcggccgcaagcttgtcgacACCAGATTGATGATTGCCTGG
62SK-SlMYC2	F: cgctctagaactagtggatccATGACTGAATACAGCTTGCCCA<br>R: gataagcttgatatcgaattcTTAGTGTGTTTCAGCAATTTTCG

pMAL-C2-SlMYC2	F: ttggccatggaggccggatccATGACTGAATACAGCTTGCCCACC<br>R: gcttgcctgcaggacgaattcTTAGTGTGTTTCAGCAATTTTCG
nLUC-SlMYC2	F: cgggggacgagctcggtaccATGACTGAATACAGCTTGCCCA<br>R: cgcgtacgagatctggtcgacTTAGTGTGTTTCAGCAATTTTCG
N-YFPC-SlMYC2	F: gacgagctgtacaagggtaccATGACTGAATACAGCTTGCCCA<br>R: tactcgaacctgcaggtcgacTTAGTGTGTTTCAGCAATTTTCG
AD-SlMYC2	F: gtaccagattacgctcatatgATGACTGAATACAGCTTGCCCA<br>R: atgcccacccgggtggaattcTTAGTGTGTTTCAGCAATTTTCG
AD-SlMYC2N	F: gtaccagattacgctcatatgATGACTGAATACAGCTTGCCCA<br>R: atgcccacccgggtggaattcTTACCAGAGCGCCGATGGGTC
AD-SlMYC2C	F: gtaccagattacgctcatatgATGCTCACTGATCCATCGTCCTCA<br>R: atgcccacccgggtggaattcTTAGTGTGTTTCAGCAATTTTCG
BD-SlMYC2	F: tcagaggaggacctgcatatgATGACTGAATACAGCTTGCCCA<br>R: cgacggatccccgggaattcTTAGTGTGTTTCAGCAATTTTCG
BD-SlMYC2N	F: tcagaggaggacctgcatatgATGACTGAATACAGCTTGCCCA<br>R: cgacggatccccgggaattcTTACCAGAGCGCCGATGGGTC
BD-SlMYC2C	F: tcagaggaggacctgcatatgATGCTCACTGATCCATCGTCCTCA<br>R: cgacggatccccgggaattcTTAGTGTGTTTCAGCAATTTTCG
AD-SlNATA1	F: gtaccagattacgctcatatgATGGCTTCATCTTTATCTGAAACA<br>R: atgcccacccgggtggaattcTCAGTTTCCTCCATCATTTTTCTC
pCAMBIA 1305-SlNATA1	F: acggaattcgagctcggtaccATGGCTTCATCTTTATCTGAAACA<br>R: caggtcgactctagaggatccGTTTCCTCCATCATTTTTCTC
nLUC-SlNATA1	F: cgggggacgagctcggtaccATGGCTTCATCTTTATCTGAAACA<br>R: cgcgtacgagatctggtcgacTCAGTTTCCTCCATCATTTTTCTC
miniLUC-pSlPSY1	F: ttcctgcagcccgggggatccTAGGAATTGAGCACTTGGCT<br>R: ttgtctagaactagtggatccGCAAGAACACTGAGTGGATT
jai1-1	GAAAATGTTTCCACACCGTGTTCT
jai1-2	GACAGATGAAGAAACCATTTCTT
jai1-3	CAAGAACCAGAACAAATAGACTGA
QPCR-SlUBQ	F: TCGTAAGGAGTGCCCTAATGCTGA<br>R: CAATCGCCTCCAGCCTTGTTGTAA
QPCR-SlActin	F: TTGCTGACCGTATGAGCAAG<br>R: GGACAATGGATGGACCAGAC
QPCR-SlPIF1a	F: GACTTCCGAATTTTAGTGATCC<br>R: GATTTACTATTACTGGGCTGTTCT
QPCR-SlPSY1	F: AGAATCAATAGAGGTGGTGGAA<br>R: TCTCGGGAGTCATTAGCATAGT
QPCR-SlDXS	F: AGCTTCCGGCTGGAAACAAA<br>R: CTAGCACAATAGCAGCATCC
QPCR-SlGGPS	F: GTACCTCGCTACCGCTACA<br>R: TAATCCCACATTAGGGTTACC
QPCR-SlPDS	F: TGGGTGGTTTGTCTACAGCAAA<br>R: ATCCCTTGCCTCCAGCAGTA
Q-SlACS2	F: GAAAGAGTTGTTATGGCTGGTG<br>R: GCTGGGTAGTATGGTGAAGGT

Q-SlACO1	F: CCTCCCATGCGCCACTCTAT
	R: CGTGTCCCGTCTGTTTGTGC
Q-SlDXR	F: GGCTCTATAGGAACTCAGACAC
	R: CCTGATCAGCAAGAAGAGTCAC
Q-SlMCT	F: CACCACTACATCTGACTCCTTG
	R: CAATGGTCCTCATAAAGCCTCA
Q-SlCMK	F: CAATGGTCCTCATAAAGCCTCA
	R: GGCAGGAGGTTCTAAATCATTG
Q-SlMDS	F: TAGAGCCAACTTGTGTAAGCTG
	R: ATACTTCTGTTCTCACCGAGAC
Q-SlZDS	F: TTGGACAATCTCCTCTATACGC
	R: TTGATAGAGGCATGTAAGGGTC
Q-SlZISO	F: CCTTCTTCTTCCTATACCCGTC
	R: TGCCTGGTAATCCTCATAATCC
Q-SlCRTISO	F: GGACTCTCTCCGAAAGACTATG
	R: GTTCCCACCTCCTTAAAGAGAA
Q-SlLCYB	F: TAGTGTCCTTGCCACCATATAAC
	R: GTGGTCCACTTCCAGTATTACC
Q-SlOR	F: GAGTGCTGAGCTTGGGATAATA
	R: CCGGCAATGAGGGATATACAA

Supplementary Table 2. EMSA probes in this work.

Probe name	Probe sequence
Probe-SlPIF1a	F: GAAAAGATGTTAATACCAATCGACACATGAAATTTGTATGATCACAACCTAAT
	R: ATTAGGTTGTGATCATACAAATTTCATGTGTCGATTGGTATTAACATCTTTTC
Mu-Probe-SlPIF1a	F: GAAAAGATGTTAATACCAATCGATCAAGTAAATTTGTATGATCACAACCTAAT
	R: ATTAGGTTGTGATCATACAAATTTACTTGATCGATTGGTATTAACATCTTTTC

Supplementary Data set1. Source data and statistical summary.

Fig. 1B-1D										
		lycopene			β-carotene			lutein		
	Light	37.012	39.023	41.01	18.812	18.807	18.864	4.723	4.794	4.71
Control	Dark	15.257	15.835	14.877	13.429	13.782	12.883	3.658	3.736	3.228
	Light	45.468	44.976	46.274	19.911	20.859	19.971	1.997	2.087	2.095
MeJA	Dark	0.577	0.582	0.697	2.797	2.836	2.781	3.746	3.806	3.71
	Light	64.918	64.378	62.934	21.072	20.788	20.466	3.846	3.869	3.776
Slst2a	Dark	1.006	0.952	0.963	4.26	4.292	4.102	3.732	3.585	3.641

Fig. 1B-light

Test details	Mean 1	Mean 2	Mean Diff.	SE of diff.	95.00% CI of diff.	Significant?	Summary
Control vs. MeJA	39.02	45.57	-6.558	0.7969	-9.061 to -4.054	Yes	****
Control vs. Slst2a	39.02	64.08	-25.06	0.7969	-27.56 to -22.56	Yes	****
MeJA vs. Slst2a	45.57	64.08	-18.5	0.7969	-21.01 to -16.00	Yes	****

Fig. 1B-Dark

Test details	Mean 1	Mean 2	Mean Diff.	SE of diff.	95.00% CI of diff.	Significant?	Summary
Control vs. MeJA	15.32	0.6187	14.7	0.7969	12.20 to 17.21	Yes	****
Control vs. Slst2a	15.32	0.9737	14.35	0.7969	11.85 to 16.85	Yes	****
MeJA vs. Slst2a	0.6187	0.9737	-0.355	0.7969	-2.858 to 2.148	No	ns

Fig. 1C-light

Test details	Mean 1	Mean 2	Mean Diff.	SE of diff.	95.00% CI of diff.	Significant?	Summary
Control vs. MeJA	18.83	20.25	-1.419	0.2563	-2.224 to -0.6144	Yes	***
Control vs. Slst2a	18.83	20.78	-1.948	0.2563	-2.753 to -1.143	Yes	****
MeJA vs. Slst2a	20.25	20.78	-0.5283	0.2563	-1.333 to 0.2766	No	ns

Fig. 1C-Dark

Test details	Mean 1	Mean 2	Mean Diff.	SE of diff.	95.00% CI of diff.	Significant?	Summary
Control vs. MeJA	13.36	2.805	10.56	0.2563	9.755 to 11.36	Yes	****
Control vs. Slst2a	13.36	4.218	9.147	0.2563	8.342 to 9.952	Yes	****
MeJA vs. Slst2a	2.805	4.218	-1.413	0.2563	-2.218 to -0.6084	Yes	***

Fig. 1D-light							
Test details	Mean 1	Mean 2	Mean Diff.	SE of diff.	95.00% CI of diff.	Significant ?	Summar y
Control vs. MeJA	4.742	2.06	2.683	0.1	2.368 to 2.997	Yes	****
Control vs.Slst2a	4.742	3.83	0.912	0.1	0.5978 to 1.226	Yes	****
MeJA vs. Slst2a	2.06	3.83	-1.771	0.1	-2.085 to -1.456	Yes	****

Fig. 1D-Dark							
Test details	Mean 1	Mean 2	Mean Diff.	SE of diff.	95.00% CI of diff.	Significant ?	Summar y
Control vs.MeJA	3.541	3.754	-0.2133	0.1	-0.5275 to 0.1009	No	ns
Control vs.Slst2a	3.541	3.653	-0.112	0.1	-0.4262 to 0.2022	No	ns
MeJA vs. Slst2a	3.754	3.653	0.1013	0.1	-0.2129 to 0.4155	No	ns

Fig. 1E-1H							
		Control			MeJA		
SlDXS	Light	7.635905	6.821006	8.706991	19.3412	15.56049	14.64832
	Dark	3.636363	7.945322	2.478871	3.966418	2.039074	2.477727
SlGGPS	Light	1.692073	5.052175	1.206745	8.727307	7.437037	11.57198
	Dark	0.187526	0.616975	0.245631	0.157932	0.042831	0.100197
SlPSY1	Light	1.2123	0.7585	1.0874	2.8626	2.9922	3.1038
	Dark	0.3776	0.5587	0.2932	0.104	0.0472	0.0607
SlPDS	Light	1.517395	1.155431	0.57037	4.439935	3.856892	4.76559
	Dark	0.814617	0.726398	0.345407	0.466461	0.373944	0.710554

Fig. 1E-1H							
		Mean 1	Mean 2	Mean Diff.	SE of diff.	95% CI of diff.	Significant?
SlDXS	Light	7.721	16.52	-8.795	1.654	-13.33 to -4.259	Yes
	Dark	4.687	2.828	1.859	1.654	-2.677 to 6.395	No
SlGGPS	Light	2.650	9.245	-6.595	1.219	-9.940 to -3.250	Yes
	Dark	0.3500	0.1003	0.2497	1.219	-3.095 to 3.594	No
SlPSY1	Light	1.019	2.986	-1.967	0.1217	-2.301 to -1.633	Yes
	Dark	0.4098	0.07063	0.3392	0.1217	0.005456 to 0.6729	Yes
SlPDS	Light	1.081	4.354	-3.273	0.2980	-4.091 to -2.456	Yes
	Dark	0.6288	0.5170	0.1118	0.2980	-0.7056 to 0.9293	No

Fig. 2B										
		lycopene			β-carotene			lutein		
Light	WT	37.012	39.023	41	18.812	18.807	18.864	4.723	4.794	4.71
	Slpif1a-1	48.358	51.266	50.106	17.078	17.321	16.937	2.948	3.246	3.174
	Slpif1a-2	49.702	52.448	51.369	21.875	22.455	21.928	5.294	5.592	5.51
	OESlPIF1a-1	34.137	39.658	36.231	15.484	15.518	15.322	4.045	4.033	3.967
	OESlPIF1a-2	41.905	37.9	41.844	12.022	11.954	12.364	2.142	1.995	1.994
Dark	WT	15.257	15.835	14.877	13.429	13.782	12.883	3.658	3.736	3.228
	Slpif1a-1	18.778	17.783	19.615	12.948	13.043	12.945	3.611	3.687	3.39
	Slpif1a-2	23.357	23.608	23.492	12.257	12.563	12.261	3.599	3.671	3.826
	OESlPIF1a-1	1.89	1.73	1.502	7.546	7.505	7.119	3.655	3.664	3.385
	OESlPIF1a-2	0.99	0.946	0.868	4.813	4.847	4.637	4.362	4.35	4.31

Fig. 2B-Light							
	Test details	Mean 1	Mean 2	Mean Diff.	SE of diff.	95% CI of diff.	Significant?
lycopene	WT vs. *Slpif1a-1*	39.02	49.91	-10.9	0.9741	-13.72 to -8.070	Yes
	WT vs. *Slpif1a-2*	39.02	51.17	-12.16	0.9741	-14.98 to -9.333	Yes
	WT vs. OESlPIF1a-1	39.02	36.68	2.34	0.9741	-0.4857 to 5.165	No
	WT vs. OESlPIF1a-2	39.02	40.55	-1.535	0.9741	-4.360 to 1.291	No
	Slpif1a-1 vs. *Slpif1a-2*	49.91	51.17	-1.263	0.9741	-4.088 to 1.562	No
	Slpif1a-1 vs. OESlPIF1a-1	49.91	36.68	13.23	0.9741	10.41 to 16.06	Yes
	Slpif1a-1 vs. OESlPIF1a-2	49.91	40.55	9.36	0.9741	6.535 to 12.19	Yes
	Slpif1a-2 vs. OESlPIF1a-1	51.17	36.68	14.5	0.9741	11.67 to 17.32	Yes
	Slpif1a-2 vs. OESlPIF1a-2	51.17	40.55	10.62	0.9741	7.798 to 13.45	Yes
	OESlPIF1a-1 vs. OESlPIF1a-2	36.68	40.55	-3.874	0.9741	-6.700 to -1.049	Yes
β-carotene	WT vs. *Slpif1a-1*	18.83	17.11	1.716	0.9741	-1.110 to 4.541	No
	WT vs. *Slpif1a-2*	18.83	22.09	-3.258	0.9741	-6.084 to -0.4330	Yes
	WT vs. OESlPIF1a-1	18.83	15.44	3.386	0.9741	0.5610 to 6.212	Yes
	WT vs. OESlPIF1a-2	18.83	12.11	6.714	0.9741	3.889 to 9.540	Yes
	Slpif1a-1 vs. *Slpif1a-2*	17.11	22.09	-4.974	0.9741	-7.799 to -2.149	Yes
	Slpif1a-1 vs. OESlPIF1a-1	17.11	15.44	1.671	0.9741	-1.155 to 4.496	No
	Slpif1a-1 vs. OESlPIF1a-2	17.11	12.11	4.999	0.9741	2.173 to 7.824	Yes
	Slpif1a-2 vs. OESlPIF1a-1	22.09	15.44	6.645	0.9741	3.819 to 9.470	Yes
	Slpif1a-2 vs. OESlPIF1a-2	22.09	12.11	9.973	0.9741	7.147 to 12.80	Yes
	OESlPIF1a-1 vs. OESlPIF1a-2	15.44	12.11	3.328	0.9741	0.5027 to 6.153	Yes
lutein	WT vs. *Slpif1a-1*	4.742	3.123	1.62	0.9741	-1.206 to 4.445	No
	WT vs. *Slpif1a-2*	4.742	5.465	-0.723	0.9741	-3.548 to 2.102	No
	WT vs. OESlPIF1a-1	4.742	4.015	0.7273	0.9741	-2.098 to 3.553	No
	WT vs. OESlPIF1a-2	4.742	2.044	2.699	0.9741	-0.1267 to 5.524	No
	Slpif1a-1 vs. *Slpif1a-2*	3.123	5.465	-2.343	0.9741	-5.168 to 0.4827	No

	Test details	Mean 1	Mean 2	Mean Diff.	SE of diff.	95% CI of diff.	Significant?
	Slpif1a-1 vs. OESlPIF1a-1	3.123	4.015	-0.8923	0.9741	-3.718 to 1.933	No
	Slpif1a-1 vs. OESlPIF1a-2	3.123	2.044	1.079	0.9741	-1.746 to 3.904	No
	Slpif1a-2 vs. OESlPIF1a-1	5.465	4.015	1.45	0.9741	-1.375 to 4.276	No
	Slpif1a-2 vs. OESlPIF1a-2	5.465	2.044	3.422	0.9741	0.5963 to 6.247	Yes
	OESlPIF1a-1 vs. OESlPIF1a-2	4.015	2.044	1.971	0.9741	-0.8540 to 4.797	No

Fig. 2B-Dark							
	Test details	Mean 1	Mean 2	Mean Diff.	SE of diff.	95% CI of diff.	Significant?
lycopene	WT vs. *Slpif1a-1*	15.32	18.73	-3.402	0.2647	-4.170 to -2.634	Yes
	WT vs. *Slpif1a-2*	15.32	23.49	-8.163	0.2647	-8.931 to -7.395	Yes
	WT vs. OESlPIF1a-1	15.32	1.707	13.62	0.2647	12.85 to 14.38	Yes
	WT vs. OESlPIF1a-2	15.32	0.9347	14.39	0.2647	13.62 to 15.16	Yes
	Slpif1a-1 vs. *Slpif1a-2*	18.73	23.49	-4.76	0.2647	-5.528 to -3.992	Yes
	Slpif1a-1 vs. OESlPIF1a-1	18.73	1.707	17.02	0.2647	16.25 to 17.79	Yes
	Slpif1a-1 vs. OESlPIF1a-2	18.73	0.9347	17.79	0.2647	17.02 to 18.56	Yes
	Slpif1a-2 vs. OESlPIF1a-1	23.49	1.707	21.78	0.2647	21.01 to 22.55	Yes
	Slpif1a-2 vs. OESlPIF1a-2	23.49	0.9347	22.55	0.2647	21.78 to 23.32	Yes
	OESlPIF1a-1 vs. OESlPIF1a-2	1.707	0.9347	0.7727	0.2647	0.004819 to 1.541	Yes
β-carotene	WT vs. *Slpif1a-1*	13.36	12.98	0.386	0.2647	-0.3818 to 1.154	No
	WT vs. *Slpif1a-2*	13.36	12.36	1.004	0.2647	0.2365 to 1.772	Yes
	WT vs. OESlPIF1a-1	13.36	7.39	5.975	0.2647	5.207 to 6.743	Yes
	WT vs. OESlPIF1a-2	13.36	4.766	8.599	0.2647	7.831 to 9.367	Yes
	Slpif1a-1 vs. *Slpif1a-2*	12.98	12.36	0.6183	0.2647	-0.1495 to 1.386	No
	Slpif1a-1 vs. OESlPIF1a-1	12.98	7.39	5.589	0.2647	4.821 to 6.357	Yes
	Slpif1a-1 vs. OESlPIF1a-2	12.98	4.766	8.213	0.2647	7.445 to 8.981	Yes
	Slpif1a-2 vs. OESlPIF1a-1	12.36	7.39	4.97	0.2647	4.202 to 5.738	Yes
	Slpif1a-2 vs. OESlPIF1a-2	12.36	4.766	7.595	0.2647	6.827 to 8.363	Yes

	OESlPIF1a-1 vs. OESlPIF1a-2	7.39	4.766	2.624	0.2647	1.856 to 3.392	Yes
lutein	WT vs. *Slpif1a-1*	3.541	3.563	-0.022	0.2647	-0.7898 to 0.7458	No
	WT vs. *Slpif1a-2*	3.541	3.699	-0.158	0.2647	-0.9258 to 0.6098	No
	WT vs. OESlPIF1a-1	3.541	3.568	- 0.02733	0.2647	-0.7952 to 0.7405	No
	WT vs. OESlPIF1a-2	3.541	4.341	-0.8	0.2647	-1.568 to - 0.03215	Yes
	Slpif1a-1 vs. *Slpif1a-2*	3.563	3.699	-0.136	0.2647	-0.9038 to 0.6318	No
	Slpif1a-1 vs. OESlPIF1a-1	3.563	3.568	- 0.005333	0.2647	-0.7732 to 0.7625	No
	Slpif1a-1 vs. OESlPIF1a-2	3.563	4.341	-0.778	0.2647	-1.546 to - 0.01015	Yes
	Slpif1a-2 vs. OESlPIF1a-1	3.699	3.568	0.1307	0.2647	-0.6372 to 0.8985	No
	Slpif1a-2 vs. OESlPIF1a-2	3.699	4.341	-0.642	0.2647	-1.410 to 0.1258	No
	OESlPIF1a-1 vs. OESlPIF1a-2	3.568	4.341	-0.7727	0.2647	-1.541 to - 0.004819	Yes

Fig. 2C-Light				
WT	*Slpif1a-1*	*Slpif1a-2*	OESlPIF1a-1	OESlPIF1a-2
1.582055	2.125492	2.059839	1.215456	1.2205
1.582795	2.50547	2.348964	1.321878	1.781073
1.693432	2.578102	2.249822	1.539154	1.729308

Fig. 2C-Light							
Test details	Mean 1	Mean 2	Mean Diff.	SE of diff.	95% CI of diff.	Significant?	Summary
WT vs. *Slpif1a-1*	1.619	2.403	-0.7836	0.1665	-1.332 to -0.2356	Yes	**
WT vs. *Slpif1a-2*	1.619	2.22	-0.6001	0.1665	-1.148 to -0.05208	Yes	*
WT vs. OESlPIF1a-1	1.619	1.359	0.2606	0.1665	-0.2874 to 0.8086	No	ns
WT vs. OESlPIF1a-2	1.619	1.577	0.04247	0.1665	-0.5056 to 0.5905	No	ns
Slpif1a-1 vs. *Slpif1a-2*	2.403	2.22	0.1835	0.1665	-0.3646 to 0.7315	No	ns
Slpif1a-1 vs. OESlPIF1a-1	2.403	1.359	1.044	0.1665	0.4962 to 1.592	Yes	***
Slpif1a-1 vs. OESlPIF1a-2	2.403	1.577	0.8261	0.1665	0.2780 to 1.374	Yes	**
Slpif1a-2 vs. OESlPIF1a-1	2.22	1.359	0.8607	0.1665	0.3127 to 1.409	Yes	**
Slpif1a-2 vs. OESlPIF1a-2	2.22	1.577	0.6426	0.1665	0.09455 to 1.191	Yes	*
OESlPIF1a-1 vs. OESlPIF1a-2	1.359	1.577	-0.2181	0.1665	-0.7662 to 0.3299	No	ns

Fig. 2C-Dark				
WT	*Slpif1a-1*	*Slpif1a-2*	OESlPIF1a-1	OESlPIF1a-2
1.1152	1.6571	1.4958	0.6894	0.3208
0.8071	1.8956	1.8242	0.4363	0.3075
1.111	1.9701	1.9179	0.5558	0.2397

Fig. 2C-Dark							
Test details	Mean 1	Mean 2	Mean Diff.	SE of diff.	95% CI of diff.	Significant?	Summary
WT vs. *Slpif1a-1*	1.011	1.841	-0.8298	0.1291	-1.255 to -0.4049	Yes	***
WT vs. *Slpif1a-2*	1.011	1.746	-0.7349	0.1291	-1.160 to -0.3100	Yes	**
WT vs. OESlPIF1a-1	1.011	0.5605	0.4506	0.1291	0.02571 to 0.8755	Yes	*
WT vs. OESlPIF1a-2	1.011	0.2893	0.7218	0.1291	0.2969 to 1.147	Yes	**
Slpif1a-1 vs. *Slpif1a-2*	1.841	1.746	0.09497	0.1291	-0.3299 to 0.5199	No	ns
Slpif1a-1 vs. OESlPIF1a-1	1.841	0.5605	1.28	0.1291	0.8555 to 1.705	Yes	****
Slpif1a-1 vs. OESlPIF1a-2	1.841	0.2893	1.552	0.1291	1.127 to 1.976	Yes	****
Slpif1a-2 vs. OESlPIF1a-1	1.746	0.5605	1.185	0.1291	0.7606 to 1.610	Yes	****
Slpif1a-2 vs. OESlPIF1a-2	1.746	0.2893	1.457	0.1291	1.032 to 1.882	Yes	****
OESlPIF1a-1 vs. OESlPIF1a-2	0.5605	0.2893	0.2712	0.1291	-0.1537 to 0.6961	No	ns

Fig. 3A	
WT	MeJA
1.098436	2.975585
0.906918	2.874077
1.003823	2.990034

Unpaired *t* test
P value < 0.0001
P value summary ****
Significantly different? (*P* < 0.05) Yes
One- or two-tailed P value? Two-tailed

Fig. 3B						
		CK			JA	
WT	1.321216	0.907861	0.833694	0.546872	0.584048	0.48938
myc2	1.352648	1.225744	1.533264	1.253827	1.426036	1.508185

Fig. 3B							
Test details	Mean 1	Mean 2	Mean Diff.	SE of diff.	95% CI of diff.	Significant?	Summary
WT:CK vs. WT:JA	1.021	0.5401	0.4808	0.1366	0.04327 to 0.9184	Yes	*
WT:CK vs. myc2:CK	1.021	1.371	-0.3496	0.1366	-0.7872 to 0.08792	No	ns
WT:CK vs. myc2:JA	1.021	1.396	-0.3751	0.1366	-0.8126 to 0.06246	No	ns
WT:JA vs. myc2:CK	0.5401	1.371	-0.8305	0.1366	-1.268 to -0.3929	Yes	**
WT:JA vs. myc2:JA	0.5401	1.396	-0.8559	0.1366	-1.293 to -0.4184	Yes	**
myc2:CK vs. myc2:JA	1.371	1.396	-0.02546	0.1366	-0.4630 to 0.4121	No	ns

Fig. 3E-P1	
+62SK	+SlMYC2-62SK
1	0.857143
1.428571	1.285714
1	0.714286
1.285714	1.142857
1.142857	1

Unpaired *t* test
P value 0.2268
P value summary ns
Significantly different? (*P* < 0.05) No

Fig. 3E-P2	
+62SK	+SlMYC2-62SK
1.026316	0.5075188
0.9473684	0.498365
0.9924812	0.4849624
0.9924812	0.4398496
0.981203	0.6203008
1.06015	0.4285714

Unpaired *t* test	
P value	< 0.0001
P value summary	****
Significantly different? (*P* < 0.05)	Yes

Fig. 3I-3K										
		lycopene			β-carotene			lutein		
Control	WT	37.012	39.023	41	18.812	18.807	18.864	4.723	4.794	4.71
	Slpif1a-1	48.358	51.266	50.106	17.078	17.321	16.937	2.948	3.246	3.174
	Slpif1a-2	49.702	52.448	51.369	21.875	22.455	21.928	5.294	5.592	5.51
	OESlPIF1a-1	34.137	39.658	36.231	15.484	15.518	15.322	4.045	4.033	3.967
	OESlPIF1a-2	41.905	37.9	41.844	12.022	11.954	12.364	2.142	1.995	1.994
MeJA	WT	45.468	44.976	46.274	19.911	20.859	19.971	1.997	2.087	2.095
	Slpif1a-1	62.556	61.358	60.033	19.386	19.08	18.72	3.041	2.88	2.768
	Slpif1a-2	57.15	56.173	57.443	22.596	21.376	20.901	3.025	4.86	2.993
	OESlPIF1a-1	19.072	19.792	20.302	13.704	14.086	14.362	3.652	3.759	3.606
	OESlPIF1a-2	14.897	15.392	15.222	11.478	14.085	13.978	3.317	3.32	3.424

Fig. 3I						
Control - MeJA	Mean 1	Mean 2	Mean Diff.	SE of diff.	95% CI of diff.	Significant?
WT	39.01	45.57	-6.561	1.264	-10.15 to -2.975	Yes
Slpif1a-1	49.91	61.32	-11.41	1.264	-14.99 to -7.820	Yes
Slpif1a-2	51.17	56.92	-5.749	1.264	-9.335 to -2.163	Yes
OESlPIF1a-1	36.68	19.72	16.95	1.264	13.37 to 20.54	Yes
OESlPIF1a-2	40.55	15.17	25.38	1.264	21.79 to 28.97	Yes

Fig. 3J						
Control - MeJA	Mean 1	Mean 2	Mean Diff.	SE of diff.	95% CI of diff.	Significant?
WT	18.83	20.25	-1.419	0.4929	-2.817 to -0.02149	Yes
Slpif1a-1	17.11	19.06	-1.95	0.4929	-3.348 to -0.5522	Yes
Slpif1a-2	22.09	21.62	0.4617	0.4929	-0.9362 to 1.860	No
OESlPIF1a-1	15.44	14.05	1.391	0.4929	-0.007175 to 2.789	No
OESlPIF1a-2	12.11	13.18	-1.067	0.4929	-2.465 to 0.3308	No

Fig. 3K						
Control - MeJA	Mean 1	Mean 2	Mean Diff.	SE of diff.	95% CI of diff.	Significant?
WT	4.742	2.06	2.683	0.2867	1.870 to 3.496	Yes
Slpif1a-1	3.123	2.896	0.2263	0.2867	-0.5868 to 1.039	No
Slpif1a-2	5.465	3.626	1.839	0.2867	1.026 to 2.652	Yes
OESlPIF1a-1	4.015	3.672	0.3427	0.2867	-0.4704 to 1.156	No
OESlPIF1a-2	2.044	3.354	-1.31	0.2867	-2.123 to -0.4969	Yes

Fig. 3L-3N										
		lycopene			β-carotene			lutein		
Control	WT	15.257	15.835	14.877	13.429	13.782	12.883	3.658	3.736	3.228
	Slpif1a-1	18.778	17.783	19.615	12.948	13.043	12.945	3.611	3.687	3.39
	Slpif1a-2	23.357	23.608	23.492	12.257	12.563	12.261	3.599	3.671	3.826
	OESlPIF1a-1	1.89	1.73	1.502	7.546	7.505	7.119	3.655	3.664	3.385
	OESlPIF1a-2	0.99	0.946	0.868	4.813	4.847	4.637	4.362	4.35	4.31
MeJA	WT	0.577	0.582	0.697	2.797	2.836	2.781	3.746	3.806	3.71
	Slpif1a-1	3.609	3.57	3.834	6.607	6.538	6.541	3.041	3.88	2.768
	Slpif1a-2	13.845	14.099	14.826	6.898	6.909	7.173	4.025	2.86	2.993
	OESlPIF1a-1	0.182	0.213	0.193	2.258	2.152	2.226	3.652	3.759	3.606
	OESlPIF1a-2	0	0	0	2.049	1.958	1.886	3.317	3.32	3.424

Fig. 3L						
Control - MeJA	Mean 1	Mean 2	Mean Diff.	SE of diff.	95% CI of diff.	Significant?
WT	15.32	0.6187	14.7	0.3072	13.83 to 15.58	Yes
Slpif1a-1	18.73	3.671	15.05	0.3072	14.18 to 15.93	Yes
Slpif1a-2	23.49	14.26	9.229	0.3072	8.358 to 10.10	Yes
OESlPIF1a-1	1.707	0.196	1.511	0.3072	0.6400 to 2.383	Yes
OESlPIF1a-2	0.9347	0	0.9347	0.3072	0.06331 to 1.806	Yes

Fig. 3M						
Control - MeJA	Mean 1	Mean 2	Mean Diff.	SE of diff.	95% CI of diff.	Significant?
WT	13.36	2.805	10.56	0.1513	10.13 to 10.99	Yes
Slpif1a-1	12.98	6.562	6.417	0.1513	5.988 to 6.846	Yes
Slpif1a-2	12.36	6.993	5.367	0.1513	4.938 to 5.796	Yes
OESlPIF1a-1	7.39	2.212	5.178	0.1513	4.749 to 5.607	Yes
OESlPIF1a-2	4.766	1.964	2.801	0.1513	2.372 to 3.231	Yes

Fig. 3N						
Control - MeJA	Mean 1	Mean 2	Mean Diff.	SE of diff.	95% CI of diff.	Significant?
WT	3.541	3.754	-0.2133	0.244	-0.9052 to 0.4786	No
Slpif1a-1	3.563	3.23	0.333	0.244	-0.3589 to 1.025	No
Slpif1a-2	3.699	3.293	0.406	0.244	-0.2859 to 1.098	No
OESlPIF1a-1	3.568	3.672	-0.1043	0.244	-0.7962 to 0.5876	No
OESlPIF1a-2	4.341	3.354	0.987	0.244	0.2951 to 1.679	Yes

Fig. 4E			
62SK	1aSK	MYC2SK	1aSK+MYC2SK
0.974359	1.435897	3.897436	1.230769
1.025641	0.7692308	4.153846	1.025641
1.025641	1.076923	3.692308	1.384615
1.076923	1.025641	4.461538	1.179487
0.7179487	1.282051	3.589744	1.025641
1.179487	1.025641	4.102564	1.384615

Fig. 4E							
Test details	Mean 1	Mean 2	Mean Diff.	SE of diff.	95% CI of diff.	Significant?	Summary
62SK vs. 1aSK	1	1.103	-0.1026	0.1314	-0.4703 to 0.2652	No	ns
62SK vs. MYC2SK	1	3.983	-2.983	0.1314	-3.351 to -2.615	Yes	****
62SK vs. 1aSK+MYC2SK	1	1.205	-0.2051	0.1314	-0.5729 to 0.1626	No	ns
1aSK vs. MYC2SK	1.103	3.983	-2.88	0.1314	-3.248 to -2.513	Yes	****
1aSK vs. 1aSK+MYC2SK	1.103	1.205	-0.1026	0.1314	-0.4703 to 0.2652	No	ns
MYC2SK vs. 1aSK+MYC2SK	3.983	1.205	2.778	0.1314	2.410 to 3.146	Yes	****

Fig. 5E			
62SK	1aSK	MYC2SK	1aSK+MYC2SK
1.090218	3.193166	1.02786	3.071356
1.007196	3.156014	1.23692	2.52144
0.9104318	3.553441	0.79761	2.936547
1.027369	3.494767	0.91458	3.311294
0.7772322	2.973932	1.03679	3.093524
1.187552	3.414439	1.19876	3.175821

Fig. 5E							
Test details	Mean 1	Mean 2	Mean Diff.	SE of diff.	95% CI of diff.	Significant?	Summary
62SK vs. 1aSK	1	3.298	-2.298	0.1202	-2.634 to -1.961	Yes	****
62SK vs. MYC2SK	1	1.035	-0.03542	0.1202	-0.3718 to 0.3010	No	ns
62SK vs. 1aSK+MYC2SK	1	3.018	-2.018	0.1202	-2.355 to -1.682	Yes	****
1aSK vs. MYC2SK	3.298	1.035	2.262	0.1202	1.926 to 2.599	Yes	****
1aSK vs. 1aSK+MYC2SK	3.298	3.018	0.2793	0.1202	-0.05709 to 0.6157	No	ns
MYC2SK vs. 1aSK+MYC2SK	1.035	3.018	-1.983	0.1202	-2.319 to -1.647	Yes	****

Fig. 5G									
	lycopene			β-carotene			lutein		
WT	16.5	22.5	22.3	10.8	13.2	10.4	4.57	5.64	3.67
Slpif1a-3	34	29.4	44	11.2	9.95	8.25	7.51	7.35	4.05

Fig. 5G							
Test details	Mean 1	Mean 2	Mean Diff.	SE of diff.	95% CI of diff.	Significant?	Summary
lycopene	20.43	35.8	-15.37	2.918	-23.45 to -7.284	Yes	***
β-carotene	11.47	9.8	1.667	2.918	-6.416 to 9.749	No	ns
lutein	4.627	6.303	-1.677	2.918	-9.759 to 6.406	No	ns

Fig. 6A	
MG	BR
0.9286	0.0919
1.1843	0.0851
0.9093	0.1126

Unpaired t test	
P value	0.0005
P value summary	***
Significantly different? (P < 0.05)	Yes

Fig. 6B	
CK	JA
1.7995	8.4432
1.7535	9.6187
2.7087	9.4606

Unpaired t test	
P value	0.0001
P value summary	***
Significantly different? (P < 0.05)	Yes

Fig. 6D				
62SK	1aSK	1aSK+FLAG	1aSK+NATA1-FLAG	NATA-FLAG
1.099868	0.7830927	1.021795	0.3481461	0.8854277
1.017785	1.046561	0.7948863	0.262053	0.9779764
0.9116426	0.8439475	0.7613926	0.4203227	0.9842283
0.9831116	0.9640061	1.068497	0.3771582	0.9710711
0.9875932	0.9069252	0.9496179	0.3198415	0.8366355

Fig. 6D							
Test details	Mean 1	Mean 2	Mean Diff.	SE of diff.	95% CI of diff.	Significant?	Summary
62SK vs. 1aSK	1	0.9089	0.09109	0.05771	-0.08160 to 0.2638	No	ns
62SK vs. 1aSK+FLAG	1	0.9192	0.08076	0.05771	-0.09193 to 0.2535	No	ns
62SK vs. 1aSK+NATA1-FLAG	1	0.3455	0.6545	0.05771	0.4818 to 0.8272	Yes	****
62SK vs. NATA-FLAG	1	0.9311	0.06893	0.05771	-0.1038 to 0.2416	No	ns
1aSK vs. 1aSK+FLAG	0.9089	0.9192	-0.01033	0.05771	-0.1830 to 0.1624	No	ns
1aSK vs. 1aSK+NATA1-FLAG	0.9089	0.3455	0.5634	0.05771	0.3907 to 0.7361	Yes	****
1aSK vs. NATA-FLAG	0.9089	0.9311	-0.02216	0.05771	-0.1949 to 0.1505	No	ns
1aSK+FLAG vs. 1aSK+NATA1-FLAG	0.9192	0.3455	0.5737	0.05771	0.4010 to 0.7464	Yes	****
1aSK+FLAG vs. NATA-FLAG	0.9192	0.9311	-0.01183	0.05771	-0.1845 to 0.1609	No	ns
1aSK+NATA1-FLAG vs. NATA-FLAG	0.3455	0.9311	-0.5856	0.05771	-0.7583 to -0.4129	Yes	****

Fig. S1							
		Control			MeJA		
SlDXR	Light	1.158206	0.864428	0.998816	2.482282	2.469931	2.196598
	Dark	0.670066	0.588583	0.387883	2.356896	1.74388	1.735641
SlMCT	Light	0.851163	0.960614	1.223033	2.465493	1.27886	1.177151
	Dark	1.140296	1.093756	0.816761	0.687649	1.443845	1.301978
SlCMK	Light	0.878818	0.801203	1.42023	1.241922	1.392194	2.687036
	Dark	1.267344	1.528351	0.847468	0.414001	1.484254	0.868344
SlMDS	Light	0.804617	0.750064	1.656963	1.602411	1.941592	1.967566
	Dark	1.126622	1.571219	0.475726	0.768555	1.2784	1.346329
SlZDS	Light	1.099511	0.957976	0.675895	1.417974	1.274204	1.830913
	Dark	1.28698	1.134142	1.747033	0.35666	0.532284	0.640672
SlZISO	Light	0.93973	0.853564	1.246697	0.839777	1.210885	1.231737
	Dark	0.736017	0.852829	0.769097	0.398828	0.595683	0.750899
SlCRTISO	Light	0.825451	0.951642	1.273019	0.879167	1.288853	1.297581
	Dark	0.599388	0.747941	0.718538	0.344363	0.397088	0.763114
SlLCYB	Light	0.831701	0.827281	1.453383	0.84262	1.058075	1.004459
	Dark	0.331375	0.51964	0.436278	0.488072	0.356582	0.451907
SlOR	Light	0.969544	0.909167	1.134459	1.177759	2.00594	1.458406
	Dark	0.544223	0.316726	0.50747	0.70068	0.727879	0.84287

Fig. S1							
		Mean 1	Mean 2	Mean Diff.	SE of diff.	95% CI of diff.	Significant?
SlDXR	Light	1.007	2.383	-1.376	0.1806	-1.871 to -0.8803	Yes
	Dark	0.5488	1.945	-1.397	0.1806	-1.892 to -0.9011	Yes
SlMCT	Light	1.012	1.641	-0.6289	0.3516	-1.593 to 0.3356	No
	Dark	1.017	1.144	-0.1276	0.3516	-1.092 to 0.8370	No
SlCMK	Light	1.033	1.774	-0.7403	0.4381	-1.942 to 0.4615	No
	Dark	1.214	0.9222	0.2922	0.4381	-0.9096 to 1.494	No
SlMDS	Light	1.071	1.837	-0.7666	0.3424	-1.706 to 0.1727	No
	Dark	1.058	1.131	-0.07324	0.3424	-1.013 to 0.8661	No
SlZDS	Light	0.9111	1.508	-0.5966	0.2051	-1.159 to -0.03399	Yes
	Dark	1.389	0.5099	0.8795	0.2051	0.3169 to 1.442	Yes
SlZISO	Light	1.013	1.094	-0.0808	0.145	-0.4785 to 0.3169	No
	Dark	0.786	0.5818	0.2042	0.145	-0.1935 to 0.6019	No
SlCRTISO	Light	1.017	1.155	-0.1385	0.1676	-0.5984 to 0.3214	No
	Dark	0.6886	0.5015	0.1871	0.1676	-0.2728 to 0.6470	No
SlLCYB	Light	1.037	0.9684	0.06907	0.1612	-0.3730 to 0.5112	No
	Dark	0.4291	0.4322	-0.00309	0.1612	-0.4452 to 0.4390	No
SlOR	Light	1.004	1.547	-0.543	0.1878	-1.058 to -0.02779	Yes
	Dark	0.4561	0.7571	-0.301	0.1878	-0.8162 to 0.2142	No

Fig. S2A-S2B							
		Control			MeJA		
SlACS2	Light	0.8432	0.5041	0.7216	2.8707	2.0024	3.6835
	Dark	0.7169	1.0986	1.2697	2.3543	2.2665	2.1935
SlACO1	Light	0.800372	0.509103	0.521076	3.303127	1.914278	2.124797
	Dark	0.798683	1.104299	1.133806	1.703926	1.918339	1.6883

Fig. S2A

Test details	Mean 1	Mean 2	Mean Diff.	SE of diff.	Mean Diff.	Significant?	Summary
Light:Control vs. Light:MeJA	0.6896	2.852	-2.163	0.3703	-2.163	Yes	***
Light:Control vs. Dark:Control	0.6896	1.028	-0.3388	0.3703	-0.3388	No	ns
Light:Control vs. Dark:MeJA	0.6896	2.271	-1.582	0.3703	-1.582	Yes	**
Light:MeJA vs. Dark:Control	2.852	1.028	1.824	0.3703	1.824	Yes	**
Light:MeJA vs. Dark:MeJA	2.852	2.271	0.5808	0.3703	0.5808	No	ns
Dark:Control vs. Dark:MeJA	1.028	2.271	-1.243	0.3703	-1.243	Yes	**

Fig. S2B

Test details	Mean 1	Mean 2	Mean Diff.	SE of diff.	Mean Diff.	Significant?	Summary
Light:Control vs. Light:MeJA	0.6102	2.447	-1.837	0.3262	-1.837	Yes	***
Light:Control vs. Dark:Control	0.6102	1.012	-0.4021	0.3262	-0.4021	No	ns
Light:Control vs. Dark:MeJA	0.6102	1.77	-1.16	0.3262	-1.16	Yes	**
Light:MeJA vs. Dark:Control	2.447	1.012	1.435	0.3262	1.435	Yes	**
Light:MeJA vs. Dark:MeJA	2.447	1.77	0.6772	0.3262	0.6772	No	ns
Dark:Control vs. Dark:MeJA	1.012	1.77	-0.7579	0.3262	-0.7579	Yes	*

Fig. S3F		
WT	OESlPIF1a-1	OESlPIF1a-2
1.227061	9.624254	5.075562
1.180401	6.150263	6.224267
1.69254	6.269997	5.970765

Unpaired *t* test

WT vs OE-SlPIF1a-1		WT vs OE-SlPIF1a-2	
P value	0.0065	*P* value	0.0003
P value summary	**	*P* value summary	***
Significantly different? ($P < 0.05$)	Yes	Significantly different? ($P < 0.05$)	Yes
Two-tailed		Two-tailed	

Fig. S4A-S4D					
	WT	*Slpif1a-1*	*Slpif1a-2*	OESlPIF1a-1	OESlPIF1a-2
SlDXS	0.823	1.2195	1.218	1.4524	1.2378
	1.2366	1.2563	0.9077	1.4267	0.8537
	0.9826	1.2283	1.3476	1.132	0.8096
SlGGPS	0.905201	1.379173	1.706382	1.144606	1.300446
	1.042506	1.279181	1.439151	0.832401	1.114606
	1.059683	1.412356	1.433425	1.365189	0.990641
SlPDS	1.034561	2.385143	3.282461	0.552357	0.493181
	0.879623	2.228112	3.007594	0.478071	0.464801
	1.098872	2.835813	2.79575	0.445745	0.551693
SlOR	1.35379	0.920819	1.704496	0.880416	0.992204
	0.769402	0.962058	1.190949	1.094885	0.634265
	0.960053	0.855278	0.994174	0.92952	1.274772

Fig. S4A							
Test details	Mean 1	Mean 2	Mean Diff.	SE of diff.	95% CI of diff.	Significant?	Summary
WT vs. *Slpif1a-1*	1.014	1.235	-0.2206	0.1558	-0.7335 to 0.2922	No	ns
WT vs. *Slpif1a-2*	1.014	1.158	-0.1437	0.1558	-0.6566 to 0.3692	No	ns
WT vs. OESlPIF1a-1	1.014	1.337	-0.323	0.1558	-0.8358 to 0.1899	No	ns
WT vs. OESlPIF1a-2	1.014	0.967	0.04703	0.1558	-0.4658 to 0.5599	No	ns
Slpif1a-1 vs. *Slpif1a-2*	1.235	1.158	0.07693	0.1558	-0.4359 to 0.5898	No	ns
Slpif1a-1 vs. OESlPIF1a-1	1.235	1.337	-0.1023	0.1558	-0.6152 to 0.4105	No	ns
Slpif1a-1 vs. OESlPIF1a-2	1.235	0.967	0.2677	0.1558	-0.2452 to 0.7805	No	ns
Slpif1a-2 vs. OESlPIF1a-1	1.158	1.337	-0.1793	0.1558	-0.6921 to 0.3336	No	ns
Slpif1a-2 vs. OESlPIF1a-2	1.158	0.967	0.1907	0.1558	-0.3221 to 0.7036	No	ns
OESlPIF1a-1 vs. OESlPIF1a-2	1.337	0.967	0.37	0.1558	-0.1429 to 0.8829	No	ns

Fig. S4B							
Test details	Mean 1	Mean 2	Mean Diff.	SE of diff.	95% CI of diff.	Significant?	Summary
WT vs. *Slpif1a-1*	1.002	1.357	-0.3544	0.145	-0.8315 to 0.1226	No	ns
WT vs. *Slpif1a-2*	1.002	1.308	-0.3052	0.145	-0.7823 to 0.1719	No	ns
WT vs. OESlPIF1a-1	1.002	1.114	-0.1116	0.145	-0.5887 to 0.3655	No	ns
WT vs. OESlPIF1a-2	1.002	1.135	-0.1328	0.145	-0.6098 to 0.3443	No	ns
Slpif1a-1 vs. *Slpif1a-2*	1.357	1.308	0.04924	0.145	-0.4278 to 0.5263	No	ns
Slpif1a-1 vs. OESlPIF1a-1	1.357	1.114	0.2428	0.145	-0.2342 to 0.7199	No	ns
Slpif1a-1 vs. OESlPIF1a-2	1.357	1.135	0.2217	0.145	-0.2554 to 0.6987	No	ns
Slpif1a-2 vs. OESlPIF1a-1	1.308	1.114	0.1936	0.145	-0.2835 to 0.6707	No	ns
Slpif1a-2 vs. OESlPIF1a-2	1.308	1.135	0.1724	0.145	-0.3046 to 0.6495	No	ns
OESlPIF1a-1 vs. OESlPIF1a-2	1.114	1.135	-0.02117	0.145	-0.4982 to 0.4559	No	ns

Fig. S4C							
Test details	Mean 1	Mean 2	Mean Diff.	SE of diff.	95% CI of diff.	Significant?	Summary
WT vs. *Slpif1a-1*	1.004	2.483	-1.479	0.1535	-1.984 to -0.9735	Yes	****
WT vs. *Slpif1a-2*	1.004	3.029	-2.024	0.1535	-2.529 to -1.519	Yes	****
WT vs. OESlPIF1a-1	1.004	0.4921	0.5123	0.1535	0.007113 to 1.017	Yes	*
WT vs. OESlPIF1a-2	1.004	0.5032	0.5011	0.1535	-0.004054 to 1.006	No	ns
Slpif1a-1 vs. *Slpif1a-2*	2.483	3.029	-0.5456	0.1535	-1.051 to -0.04040	Yes	*
Slpif1a-1 vs. OESlPIF1a-1	2.483	0.4921	1.991	0.1535	1.486 to 2.496	Yes	****
Slpif1a-1 vs. OESlPIF1a-2	2.483	0.5032	1.98	0.1535	1.475 to 2.485	Yes	****
Slpif1a-2 vs. OESlPIF1a-1	3.029	0.4921	2.537	0.1535	2.031 to 3.042	Yes	****
Slpif1a-2 vs. OESlPIF1a-2	3.029	0.5032	2.525	0.1535	2.020 to 3.031	Yes	****
OESlPIF1a-1 vs. OESlPIF1a-2	0.4921	0.5032	-0.01117	0.1535	-0.5163 to 0.4940	No	ns

Fig. S4D							
Test details	Mean 1	Mean 2	Mean Diff.	SE of diff.	95% CI of diff.	Significant?	Summary
WT vs. *Slpif1a-1*	1.028	0.9127	0.115	0.2135	-0.5876 to 0.8177	No	ns
WT vs. *Slpif1a-2*	1.028	1.297	-0.2688	0.2135	-0.9714 to 0.4339	No	ns
WT vs. OESlPIF1a-1	1.028	0.9683	0.05947	0.2135	-0.6432 to 0.7621	No	ns
WT vs. OESlPIF1a-2	1.028	0.9671	0.06067	0.2135	-0.6420 to 0.7633	No	ns
Slpif1a-1 vs. *Slpif1a-2*	0.9127	1.297	-0.3838	0.2135	-1.086 to 0.3188	No	ns
Slpif1a-1 vs. OESlPIF1a-1	0.9127	0.9683	-0.05556	0.2135	-0.7582 to 0.6471	No	ns
Slpif1a-1 vs. OESlPIF1a-2	0.9127	0.9671	-0.05436	0.2135	-0.7570 to 0.6483	No	ns
Slpif1a-2 vs. OESlPIF1a-1	1.297	0.9683	0.3283	0.2135	-0.3744 to 1.031	No	ns
Slpif1a-2 vs. OESlPIF1a-2	1.297	0.9671	0.3295	0.2135	-0.3732 to 1.032	No	ns
OESlPIF1a-1 vs. OESlPIF1a-2	0.9683	0.9671	0.001193	0.2135	-0.7015 to 0.7038	No	ns

Fig. S5						
		WT			*jai-1*	
0h	0.25538	0.312102	0.314315	0.47669	0.39283	0.492042
1h	0.111184	0.126928	0.106917	0.298734	0.234759	0.205002
3h	0.149875	0.125126	0.136446	0.405006	0.360213	0.313621
6h	0.14091	0.162809	0.014028	0.370811	0.329397	0.247983
12h	0.015596	0.006238	0.063521	0.254499	0.257204	0.24311

Fig. S5							
Test details	Mean 1	Mean 2	Mean Diff.	SE of diff.	95% CI of diff.	Significant ?	Summary
0h:WT vs. 0h:jai	0.2939	0.4539	-0.1599	0.03649	-0.2891 to -0.03071	Yes	**
0h:WT vs. 1h:WT	0.2939	0.115	0.1789	0.03649	0.04971 to 0.3081	Yes	**
0h:WT vs. 1h:jai	0.2939	0.2462	0.04777	0.03649	-0.08145 to 0.1770	No	ns
0h:WT vs. 3h:WT	0.2939	0.1371	0.1568	0.03649	0.02757 to 0.2860	Yes	*
0h:WT vs. 3h:jai	0.2939	0.3596	-0.06568	0.03649	-0.1949 to 0.06353	No	ns
0h:WT vs. 6h:WT	0.2939	0.1059	0.188	0.03649	0.05880 to 0.3172	Yes	**
0h:WT vs. 6h:jai	0.2939	0.3161	-0.02213	0.03649	-0.1513 to 0.1071	No	ns
0h:WT vs. 12h:WT	0.2939	0.02845	0.2655	0.03649	0.1363 to 0.3947	Yes	****
0h:WT vs. 12h:jai	0.2939	0.2516	0.04233	0.03649	-0.08688 to 0.1715	No	ns
0h:jai vs. 1h:WT	0.4539	0.115	0.3388	0.03649	0.2096 to 0.4681	Yes	****
0h:jai vs. 1h:jai	0.4539	0.2462	0.2077	0.03649	0.07848 to 0.3369	Yes	***
0h:jai vs. 3h:WT	0.4539	0.1371	0.3167	0.03649	0.1875 to 0.4459	Yes	****
0h:jai vs. 3h:jai	0.4539	0.3596	0.09424	0.03649	-0.03497 to 0.2235	No	ns
0h:jai vs. 6h:WT	0.4539	0.1059	0.3479	0.03649	0.2187 to 0.4772	Yes	****
0h:jai vs. 6h:jai	0.4539	0.3161	0.1378	0.03649	0.008578 to 0.2670	Yes	*
0h:jai vs. 12h:WT	0.4539	0.02845	0.4254	0.03649	0.2962 to 0.5546	Yes	****
0h:jai vs. 12h:jai	0.4539	0.2516	0.2022	0.03649	0.07304 to 0.3315	Yes	***
1h:WT vs. 1h:jai	0.115	0.2462	-0.1312	0.03649	-0.2604 to -0.001943	Yes	*
1h:WT vs. 3h:WT	0.115	0.1371	-0.02214	0.03649	-0.1514 to 0.1071	No	ns
1h:WT vs. 3h:jai	0.115	0.3596	-0.2446	0.03649	-0.3738 to -0.1154	Yes	****
1h:WT vs. 6h:WT	0.115	0.1059	0.009094	0.03649	-0.1201 to 0.1383	No	ns
1h:WT vs. 6h:jai	0.115	0.3161	-0.2011	0.03649	-0.3303 to -0.07184	Yes	***
1h:WT vs. 12h:WT	0.115	0.02845	0.08656	0.03649	-0.04265 to 0.2158	No	ns
1h:WT vs. 12h:jai	0.115	0.2516	-0.1366	0.03649	-0.2658 to -0.007382	Yes	*
1h:jai vs. 3h:WT	0.2462	0.1371	0.109	0.03649	-0.02020 to 0.2382	No	ns
1h:jai vs. 3h:jai	0.2462	0.3596	-0.1134	0.03649	-0.2427 to 0.01576	No	ns
1h:jai vs. 6h:WT	0.2462	0.1059	0.1402	0.03649	0.01104 to 0.2695	Yes	*
1h:jai vs. 6h:jai	0.2462	0.3161	-0.0699	0.03649	-0.1991 to 0.05931	No	ns
1h:jai vs. 12h:WT	0.2462	0.02845	0.2177	0.03649	0.08850 to 0.3469	Yes	***
1h:jai vs. 12h:jai	0.2462	0.2516	-0.005439	0.03649	-0.1347 to 0.1238	No	ns
3h:WT vs. 3h:jai	0.1371	0.3596	-0.2225	0.03649	-0.3517 to -0.09325	Yes	***

3h:WT vs. 6h:WT	0.1371	0.1059	0.03123	0.03649	-0.09798 to 0.1604	No	ns
3h:WT vs. 6h:jai	0.1371	0.3161	-0.1789	0.03649	-0.3081 to -0.04970	Yes	**
3h:WT vs. 12h:WT	0.1371	0.02845	0.1087	0.03649	-0.02052 to 0.2379	No	ns
3h:WT vs. 12h:jai	0.1371	0.2516	-0.1145	0.03649	-0.2437 to 0.01476	No	ns
3h:jai vs. 6h:WT	0.3596	0.1059	0.2537	0.03649	0.1245 to 0.3829	Yes	****
3h:jai vs. 6h:jai	0.3596	0.3161	0.04355	0.03649	-0.08566 to 0.1728	No	ns
3h:jai vs. 12h:WT	0.3596	0.02845	0.3312	0.03649	0.2019 to 0.4604	Yes	****
3h:jai vs. 12h:jai	0.3596	0.2516	0.108	0.03649	-0.02120 to 0.2372	No	ns
6h:WT vs. 6h:jai	0.1059	0.3161	-0.2101	0.03649	-0.3394 to -0.08094	Yes	***
6h:WT vs. 12h:WT	0.1059	0.02845	0.07746	0.03649	-0.05175 to 0.2067	No	ns
6h:WT vs. 12h:jai	0.1059	0.2516	-0.1457	0.03649	-0.2749 to -0.01648	Yes	*
6h:jai vs. 12h:WT	0.3161	0.02845	0.2876	0.03649	0.1584 to 0.4168	Yes	****
6h:jai vs. 12h:jai	0.3161	0.2516	0.06446	0.03649	-0.06475 to 0.1937	No	ns
12h:WT vs. 12h:jai	0.02845	0.2516	-0.2232	0.03649	-0.3524 to -0.09394	Yes	***

Fig. S6B										
			lycopene			β-carotene			lutein	
WT	Light	39.022	39.003	40	18.812	18.807	18.864	4.723	4.794	4.71
	Dark	15.257	15.835	14.877	13.429	13.782	12.883	3.658	3.736	3.228
Slmyc2	Light	19.496	21.904	21.352	13.844	13.94	14.218	2.383	2.555	2.725
	Dark	15.153	14.937	14.336	10.672	10.775	10.866	2.504	2.592	2.627

Fig. S6B								
	Test details	Mean 1	Mean 2	Mean Diff.	SE of diff.	95% CI of diff.	Significant?	Summary
Light	lycopene	39.34	20.92	18.42	0.4698	17.12 to 19.73	Yes	****
	β-carotene	18.83	14	4.827	0.4698	3.525 to 6.129	Yes	****
	lutein	4.742	2.554	2.188	0.4698	0.8865 to 3.490	Yes	**
Dark	lycopene	15.32	14.81	0.5143	0.28	-0.2612 to 1.290	No	ns
	β-carotene	13.36	10.77	2.594	0.28	1.818 to 3.369	Yes	****
	lutein	3.541	2.574	0.9663	0.28	0.1908 to 1.742	Yes	*

Fig. S7B	
62SK	1aSK
1	1.105263
0.8421053	1.210526
1.052632	1.157895
1.105263	1.105263
0.9473684	0.8947368
1.052632	1.052632

Unpaired *t* test	
P value	0.166
P value summary	ns
Significantly different? ($P < 0.05$)	No
Two-tailed	

Fig. S9B									
	lycopene			β-carotene			lutein		
WT	26	35.4	27.5	11.4	13.1	12.4	5.18	3.84	5.14
Slpif1a-3	18.7	18.2	16.8	7.83	9.85	8.89	3.47	6.33	5.45

Fig. S9B							
Test details	Mean 1	Mean 2	Mean Diff.	SE of diff.	95% CI of diff.	Significant?	Summary
lycopene	29.63	17.9	11.73	1.854	6.596 to 16.87	Yes	***
β-carotene	12.3	8.857	3.443	1.854	-1.694 to 8.581	No	ns
lutein	4.72	5.083	-0.3633	1.854	-5.501 to 4.774	No	ns

Fig. S11B		
WT	OE-NATA1-1	OE-NATA1-2
0.8888	11.7887	45.1335
1.0441	13.1119	38.5005
1.0776	15.4018	51.1681

Unpaired t test

WT vs OE-NATA1-1		WT vs OE-NATA1-2	
P value	0.0003	P value	0.0003
P value summary	***	P value summary	***
Significantly different? (P < 0.05)	Yes	Significantly different? (P < 0.05)	Yes

Fig. S14B

62SK	MYC2SK	PIF1aSK	NATA+SK	MYC2+PIF1a	MYC2+NATA1	PIF1a+NATA1	MYC2+PIF1a+NATA1
1	2.173913043	1.217391304	1.043478261	1.086956522	2.173913043	0.739130435	0.565217391
1.043478261	2.217391304	1.173913043	0.913043478	1.173913043	2.086956522	0.652173913	0.782608696
1.130434783	1.826086957	0.869565217	0.695652174	0.695652174	1.52173913	0.260869565	0.434782609
1.086956522	2.043478261	1.086956522	0.913043478	0.956521739	1.913043478	0.52173913	0.608695652
0.913043478	2.434782609	1	1.260869565	0.826086957	1.826086957	0.304347826	0.347826087

Fig. S14B

Test details	Mean 1	Mean 2	Mean diff.	SE of diff.	95.00% CI of diff.	Below threshold?	Summary
62SK vs. MYC2SK	1.035	2.139	-1.104	0.1212	-1.497 to -0.7118	Yes	****
62SK vs. PIF1aSK	1.035	1.07	-0.03478	0.1212	-0.4274 to 0.3578	No	ns
62SK vs. NATA+SK	1.035	0.9652	0.06957	0.1212	-0.3230 to 0.4622	No	ns
62SK vs. MYC2+PIF1a	1.035	0.9478	0.08696	0.1212	-0.3056 to 0.4795	No	ns
62SK vs. MYC2+NATA1	1.035	1.904	-0.8696	0.1212	-1.262 to -0.4770	Yes	****
62SK vs. PIF1a+NATA1	1.035	0.4957	0.5391	0.1212	0.1465 to 0.9317	Yes	**
62SK vs. MYC2+PIF1a+NATA1	1.035	0.5478	0.487	0.1212	0.09437 to 0.8795	Yes	**
MYC2SK vs. PIF1aSK	2.139	1.07	1.07	0.1212	0.6770 to 1.462	Yes	****
MYC2SK vs. NATA+SK	2.139	0.9652	1.174	0.1212	0.7813 to 1.566	Yes	****
MYC2SK vs. MYC2+PIF1a	2.139	0.9478	1.191	0.1212	0.7987 to 1.584	Yes	****
MYC2SK vs. MYC2+NATA1	2.139	1.904	0.2348	0.1212	-0.1578 to 0.6274	No	ns
MYC2SK vs. PIF1a+NATA1	2.139	0.4957	1.643	0.1212	1.251 to 2.036	Yes	****
MYC2SK vs. MYC2+PIF1a+NATA1	2.139	0.5478	1.591	0.1212	1.199 to 1.984	Yes	****
PIF1aSK vs. NATA+SK	1.07	0.9652	0.1043	0.1212	-0.2882 to 0.4969	No	ns
PIF1aSK vs. MYC2+PIF1a	1.07	0.9478	0.1217	0.1212	-0.2708 to 0.5143	No	ns
PIF1aSK vs. MYC2+NATA1	1.07	1.904	-0.8348	0.1212	-1.227 to -0.4422	Yes	****
PIF1aSK vs. PIF1a+NATA1	1.07	0.4957	0.5739	0.1212	0.1813 to 0.9665	Yes	**
PIF1aSK vs. MYC2+PIF1a+NATA1	1.07	0.5478	0.5217	0.1212	0.1292 to 0.9143	Yes	**
NATA+SK vs.	0.9652	0.9478	0.01739	0.1212	-0.3752 to	No	ns

MYC2+PIF1a					0.4100		
NATA+SK vs. MYC2+NATA1	0.9652	1.904	-0.9391	0.1212	-1.332 to -0.5465	Yes	****
NATA+SK vs. PIF1a+NATA1	0.9652	0.4957	0.4696	0.1212	0.07698 to 0.8622	Yes	*
NATA+SK vs. MYC2+PIF1a+NATA1	0.9652	0.5478	0.4174	0.1212	0.02481 to 0.8100	Yes	*
MYC2+PIF1a vs. MYC2+NATA1	0.9478	1.904	-0.9565	0.1212	-1.349 to -0.5639	Yes	****
MYC2+PIF1a vs. PIF1a+NATA1	0.9478	0.4957	0.4522	0.1212	0.05959 to 0.8448	Yes	*
MYC2+PIF1a vs. MYC2+PIF1a+NATA1	0.9478	0.5478	0.4	0.1212	0.007415 to 0.7926	Yes	*
MYC2+NATA1 vs. PIF1a+NATA1	1.904	0.4957	1.409	0.1212	1.016 to 1.801	Yes	****
MYC2+NATA1 vs. MYC2+PIF1a+NATA1	1.904	0.5478	1.357	0.1212	0.9639 to 1.749	Yes	****
PIF1a+NATA1 vs. MYC2+PIF1a+NATA1	0.4957	0.5478	-0.05217	0.1212	-0.4448 to 0.3404	No	ns

SI References

1. Z. Lin, S. Zhong, D. Grierson, Recent advances in ethylene research. *J Exp Bot* **60**, 3311-3336 (2009).
2. H. L. Xing, L. Dong, Z. P. Wang, H. Y. Zhang, C. Y. Han, B. Liu, X. C. Wang, Q. J. Chen, A CRISPR/Cas9 toolkit for multiplex genome editing in plants. *Bmc Plant Biol* **14**, 327 (2014).
3. S. H. Park, J. L. Morris, J. E. Park, K. D. Hirschi, R. H. Smith, Efficient and genotype-independent Agrobacterium--mediated tomato transformation. *J Plant Physiol* **160**, 1253-1257 (2003).
4. L. Liu, J. Zhang, J. Xu, Y. Li, H. Lv, F. Wang, J. Guo, T. Lin, B. Zhao, X. X. Li, Y. D. Guo, N. Zhang, SlMYC2 promotes SlLBD40-mediated cell expansion in tomato fruit development. *Plant J* **118**, 1872-1888 (2024).
